# Association of Smoking, Alcohol Consumption, Blood Pressure, Body Mass Index, and Glycemic Risk Factors With Age-Related Macular Degeneration: A Mendelian Randomization Study

**DOI:** 10.1001/jamaophthalmol.2021.4601

**Published:** 2021-11-04

**Authors:** Valerie Kuan, Alasdair Warwick, Aroon Hingorani, Adnan Tufail, Valentina Cipriani, Stephen Burgess, Reecha Sofat

**Affiliations:** 1 Institute of Health Informatics, University College London, London, United Kingdom; 2 Health Data Research UK London, University College London, London, United Kingdom; 3 University College London British Heart Foundation Research Accelerator, London, United Kingdom; 4 Institute of Cardiovascular Science, University College London, London, United Kingdom; 5 Moorfields Eye Hospital, London, United Kingdom; 6 UCL Institute of Ophthalmology, University College London, London, United Kingdom; 7 Clinical Pharmacology, William Harvey Research Institute, Queen Mary University of London, London, United Kingdom; 8 UCL Genetics Institute, University College London, London, United Kingdom; 9 Department of Public Health and Primary Care, University of Cambridge, Cambridge, United Kingdom; 10 MRC Biostatistics Unit, University of Cambridge, Cambridge, United Kingdom

## Abstract

**Importance:**

Advanced age-related macular degeneration (AMD) is a leading cause of blindness in Western countries. Causal, modifiable risk factors need to be identified to develop preventive measures for advanced AMD.

**Objective:**

To assess whether smoking, alcohol consumption, blood pressure, body mass index, and glycemic traits are associated with increased risk of advanced AMD.

**Design, Setting, Participants:**

This study used 2-sample mendelian randomization. Genetic instruments composed of variants associated with risk factors at genome-wide significance (*P* < 5 × 10^−8^) were obtained from published genome-wide association studies. Summary-level statistics for these instruments were obtained for advanced AMD from the International AMD Genomics Consortium 2016 data set, which consisted of 16 144 individuals with AMD and 17 832 control individuals. Data were analyzed from July 2020 to September 2021.

**Exposures:**

Smoking initiation, smoking cessation, lifetime smoking, age at smoking initiation, alcoholic drinks per week, body mass index, systolic and diastolic blood pressure, type 2 diabetes, glycated hemoglobin, fasting glucose, and fasting insulin.

**Main Outcomes and Measures:**

Advanced AMD and its subtypes, geographic atrophy (GA), and neovascular AMD.

**Results:**

A 1-SD increase in logodds of genetically predicted smoking initiation was associated with higher risk of advanced AMD (odds ratio [OR], 1.26; 95% CI, 1.13-1.40; *P* < .001), while a 1-SD increase in logodds of genetically predicted smoking cessation (former vs current smoking) was associated with lower risk of advanced AMD (OR, 0.66; 95% CI, 0.50-0.87; *P* = .003). Genetically predicted increased lifetime smoking was associated with increased risk of advanced AMD (OR per 1-SD increase in lifetime smoking behavior, 1.32; 95% CI, 1.09-1.59; *P* = .004). Genetically predicted alcohol consumption was associated with higher risk of GA (OR per 1-SD increase of log-transformed alcoholic drinks per week, 2.70; 95% CI, 1.48-4.94; *P* = .001). There was insufficient evidence to suggest that genetically predicted blood pressure, body mass index, and glycemic traits were associated with advanced AMD.

**Conclusions and Relevance:**

This study provides genetic evidence that increased alcohol intake may be a causal risk factor for GA. As there are currently no known treatments for GA, this finding has important public health implications. These results also support previous observational studies associating smoking behavior with risk of advanced AMD, thus reinforcing existing public health messages regarding the risk of blindness associated with smoking.

## Key Points

**Question:**

Are smoking, alcohol intake, blood pressure, body mass index, and glycemic traits associated with age-related macular degeneration (AMD)?

**Findings:**

In this mendelian randomization study, genetically predicted smoking initiation and lifetime smoking were associated with elevated risk of advanced AMD, genetically predicted smoking cessation was associated with decreased risk of advanced AMD, and genetically predicted alcohol intake was associated with increased risk of geographic atrophy.

**Meaning:**

These findings support a potential causal association of alcohol consumption with an increased risk of geographic atrophy, smoking initiation and lifetime smoking with an increased risk of advanced AMD, and smoking cessation with a decreased risk of advanced AMD.

## Teaser

This observational analysis assesses the effects of certain lifestyle risk factors on age-related macular degeneration.

## Introduction

Age-related macular degeneration (AMD) is a leading cause of blindness in Western countries,^1,2,3,4^ accounting for 8.7% of blindness worldwide.^5^ The prevalence of AMD is projected to increase by 47% in the next 20 years because of population aging, posing a major burden to health care systems across the world.^4^ Advanced AMD consists of geographic atrophy (GA) and neovascular AMD (nAMD).^6^ Treatment is currently only available for nAMD and comes in the form of intravitreal injections of anti–vascular endothelial growth factor.^3^ This treatment is invasive, expensive, of limited effectiveness, and poses a considerable burden on patients.^7,8,9^ Therefore, increased public health efforts need to be directed toward prevention of advanced AMD. Identifying causal, modifiable risk factors for advanced AMD is critical to implementing interventions for prevention.

Observational epidemiological studies have investigated the associations of lifestyle and metabolic risk factors (smoking,^10,11,12^ alcohol consumption,^13,14,15,16^ obesity,^8,17,18,19^ blood pressure,^20,21,22^ glycemic traits,^21,23,24,25^ and dyslipidemia^2,26,27^) with AMD, but the findings are inconsistent and cannot be established as causal owing to limitations introduced by confounding and reverse causality. Randomized clinical trials (RCTs) allow reliable causal inferences to be drawn and have shown that dietary intake of specific high-dose combinations of zinc and antioxidants reduces progression from intermediate to advanced AMD.^28,29,30^ However, for diseases of aging, such as AMD, where there may be a long lag time between exposure to risk factors and clinical manifestation of disease, RCTs may be prohibitively expensive, time consuming, and infeasible, particularly for exposures such as smoking, alcohol intake, obesity, hypertension, glycemic traits, and blood lipid levels.

Mendelian randomization (MR) is a technique that has been used to assess potential causal associations across a wide range of diseases. MR is based on the principle that if a genetic variant causes a change in an exposure (eg, smoking or alcohol intake), and if this exposure is causal for a disease (eg, advanced AMD), then the genetic variant should also be associated with risk of the disease. This method exploits the random allocation of genetic variants at meiosis,^31^ rendering MR studies similar to RCTs in that they are less prone to confounding than classical observational epidemiological studies. MR studies also mitigate the problem of reverse causation because the genotype is invariant and not modified by disease.^32^ MR techniques based on multiple genetic variants selected from across the genome have found a causal role for increased levels of high-density lipoprotein cholesterol, but not low-density lipoprotein cholesterol or triglycerides, in advanced AMD risk.^33,34^

Here we use MR to assess the potential causal role of other exposures that are amenable to intervention (ie, smoking, alcohol intake, body mass index [BMI], blood pressure, and glycemic traits) on the risk of advanced AMD and its subtypes, GA and nAMD, using publicly available data.

## Methods

### Study Design

Two-sample MR was performed, whereby summary-level data of genetic variants associated with smoking initiation (ever having smoked regularly), smoking cessation (former vs current smoking), lifetime smoking (represented by an index which captures smoking status, duration, heaviness, and cessation), age at smoking initiation, weekly alcohol intake, BMI, systolic and diastolic blood pressure, type 2 diabetes, glycated hemoglobin (HbA_1c_), fasting glucose level, and fasting insulin level were obtained from study samples that did not overlap with those for advanced AMD and its subtypes (eMethods in Supplement 1). Ethical approval for each data set had been obtained in the original studies.

### Data Sources

Summary-level statistics for smoking traits, alcohol intake, BMI, blood pressure, and glycemic traits were obtained from, to our knowledge, the largest genome-wide association studies (GWAS) for these exposures to date (eMethods in Supplement 1). Summary-level genetic association data for advanced AMD, GA, and nAMD were obtained from the International AMD Genomics Consortium (IAMDGC) 2016 data set.^35^ Data were analyzed from July 2020 to September 2021. The number of participants in each study, the race and ethnicity of the participants, and the number of single-nucleotide variants (SNVs; formerly SNPs) in the genetic instruments for each exposure are provided in the Table. The SNVs used as instrumental variables for the exposures in this study (eTables 1-12 in Supplement 1) were obtained from the studies listed in the Table. SNVs were selected according to criteria outlined in the eMethods in Supplement 1.

**Table.** Details of the Summary-Level Data

Trait or disease	Source	No. of participants and participant race or ethnicity	No. of SNVs included in the instrumental variable
Ever smoked regularly	GSCAN	1 232 091 European individuals	336
Smoking cessation (former vs current smoking)	GSCAN	547 219 European individuals	23
Lifetime smoking index	UKB	462 690 European individuals	119
Age at initiation of regular smoking	GSCAN	341 427 European individuals	7
Alcohol intake per wk	GSCAN	941 280 European individuals	85
Body mass index^a^	GIANT, UKB	694 649 European individuals	289
Systolic blood pressure	GERA, ICBP, UKB	321 262 (2% African individuals, 2% East Asian individuals, 92% European individuals, 3% Latinx individuals, <1% South Asian individuals, <1% individuals of mixed/other race or ethnicity)	89
Diastolic blood pressure	GERA, ICBP, UKB	321 262 (2% African individuals, 2% East Asian individuals, 92% European individuals, 3% Latinx individuals, <1% South Asian individuals, <1% individuals of mixed/other race or ethnicity)	103
Type 2 diabetes	DIAGRAM, GERA, UKB	62 892 Individuals with AMD and 596 424 control individuals, all European	134
HbA_1c_	MAGIC	159 940 (5% African individuals, 13% East Asian individuals, 77% European individuals, 6% South Asian individuals)	37
Fasting glucose	MAGIC	133 010 European individuals	34
Fasting insulin	MAGIC	108 557 European individuals	14
Advanced AMD	IAMDGC	16 144 Individuals with advanced AMD (10 749 with neovascular AMD, 3325 with geographic atrophy, and 2070 with mixed neovascular AMD and geographic atrophy) and 17 832 control individuals, all European	NA

Abbreviations: AMD, age-related macular degeneration; DIAGRAM, Diabetes Genetics Replication and Meta-analysis; GERA, Genetic Epidemiology Research on Adult Health and Aging; GIANT, Genetic Investigation of Anthropometric Traits; GSCAN, GWAS and Sequencing Consortium of Alcohol and Nicotine Use; IAMDGC, International Age-Related Macular Degeneration Genomics Consortium; ICBP, International Consortium for Blood Pressure; MAGIC, Meta-Analyses of Glucose and Insulin-Related Traits Consortium; NA, not applicable; SNVs, single-nucleotide variants; UKB, UK Biobank.

^a^ Calculated as weight in kilograms divided by height in meters squared.

### Statistical Analysis

We performed univariable inverse-variance–weighted (IVW) 2-sample MR analyses under a multiplicative random-effects model to examine the potential causal associations of smoking, alcohol intake, BMI, blood pressure, and glycemic risk factors with the risk of advanced AMD and its subtypes, GA and nAMD (eMethods in Supplement 1).

Additionally, we conducted sensitivity analyses using the weighted median,^36^ MR-Egger,^37^ and MR pleiotropy residual sum and outlier (MR-PRESSO)^38^ methods, as well as multivariable MR (MVMR) with smoking traits adjusted for alcohol intake and vice versa (eMethods in Supplement 1).

We analyzed 12 potentially modifiable lifestyle and metabolic exposures (Table). Wald test was used to calculate 2-tailed *P* values. Using a conservative approach, we applied a Bonferroni-corrected significance level of .004 (.05 divided by 12). Analyses were conducted using the MR, MR-PRESSO, and 2-sample MR packages in R version 3.5.0 (the R Foundation).

## Results

### Association of Smoking With Risk of Advanced AMD

Genetic predisposition to smoking initiation was associated with overall risk of advanced AMD under the IVW method (OR [odds ratio], 1.26; 95% CI, 1.13-1.40; *P* < .001) (Figure; eFigure 1 and eTable 13 in Supplement 1). This was supported by the MVMR analysis (OR, 1.19; 95% CI, 1.05-1.36; *P* = .007) and weighted median analysis (OR, 1.20; 95% CI, 1.02-1.40; *P* = .03) (Figure; eFigure 1 and eTable 13 in Supplement 1). The results were similar for nAMD (IVW: OR, 1.26; 95% CI, 1.11-1.43; *P* < .001; MVMR: OR, 1.18; 95% CI, 1.02-1.37; *P* = .02; weighted median: OR, 1.29; 95% CI, 1.08-1.54; *P* = .005). Smoking initiation was not associated with GA (IVW: OR, 1.24; 95% CI, 1.03-1.49; *P* = .02; weighted median: OR, 1.31; 95% CI, 1.00-1.72; *P* = .05) (Figure; eTable 13 in Supplement 1). There was no indication of heterogeneity using the Cochran *Q* statistic (Figure) or directional pleiotropy using MR-Egger and MR-PRESSO methods (eTable 13 in Supplement 1).

**Figure.**
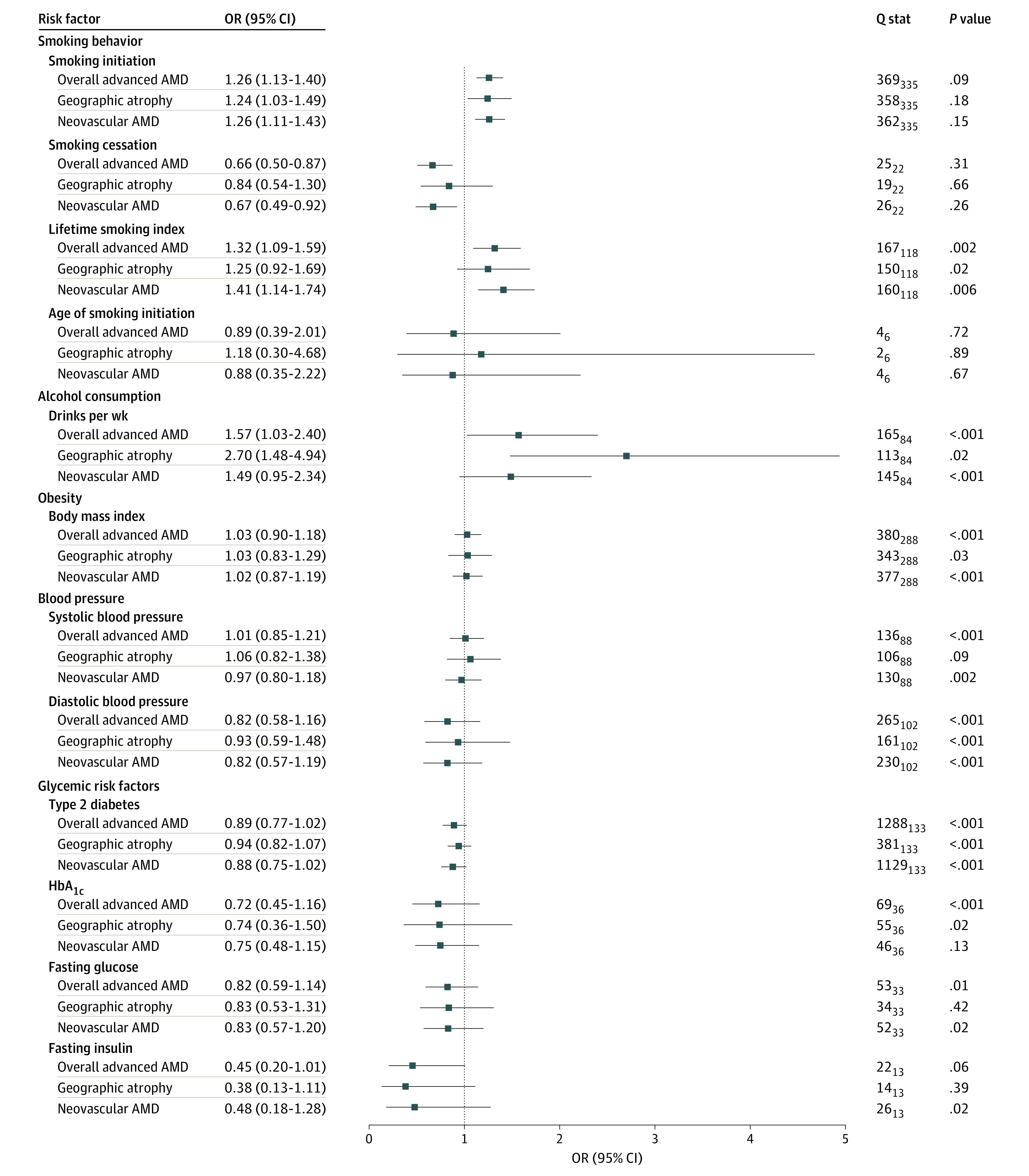
Association of Genetically Predicted Modifiable Risk Factors With Advanced Age-Related Macular Degeneration (AMD) and its Subtypes Odds ratios under the inverse-variance weighted mendelian randomization method are shown for a 1-SD increase in the logodds of ever having smoked regularly, 1-SD increase in the logodds of smoking cessation (former vs current smoking), 1-SD increase in the lifetime smoking index, 1-SD increase of the age at which an individual started smoking regularly, 1-SD increase of log-transformed alcoholic drinks per week, 1-SD increase in body mass index (calculated as 4.8 kg/m^2^); 10-mm Hg increase in systolic and diastolic pressure; 1-SD increase in the logodds of having type 2 diabetes; 1% increase in glycated hemoglobin (HbA_1c_); 18.02-mg/dL (to convert to mmol/L, multiply by 0.0555) increase in fasting glucose; and 1 log-transformed (pmol/L) increase in fasting insulin.

Genetically predicted smoking cessation was protective for advanced AMD compared with persistent smoking (IVW: OR, 0.66; 95% CI, 0.50-0.87; *P* = .003; MVMR: OR, 0.71; 95% CI, 0.57-0.88; *P* = .002; MR-Egger: OR, 0.43; 95% CI, 0.21-0.87; *P* = .02) (Figure; eFigure 2 and eTable 13 in Supplement 1). There was no association between smoking cessation and nAMD or GA (Figure; eTable 13 in Supplement 1). No evidence was found of heterogeneity using the Cochran *Q* statistic (Figure) or directional pleiotropy using MR-Egger and MR-PRESSO (eTable 13 in Supplement 1).

Genetically predicted lifetime smoking (represented by a composite index taking into account smoking status, duration, heaviness, and cessation)^39^ was associated with higher odds of advanced AMD (IVW: OR, 1.32; 95% CI, 1.09-1.59; *P* = .004; MVMR: OR, 1.48; 95% CI, 1.14-1.94; *P* = .004; weighted median: OR, 1.42; 95% CI, 1.11-1.81; *P* = .005) (Figure; eFigure 3 and eTable 13 in Supplement 1). The results were similar for nAMD (IVW: OR, 1.41; 95% CI, 1.14-1.74; *P* = .001; MVMR: OR, 1.62; 95% CI, 1.19-2.19; *P* = .002; weighted median: OR, 1.42; 95% CI, 1.08-1.88; *P* = .01) (Figure; eTable 13 in Supplement 1). The association with GA was not statistically significant. Heterogeneity was detected in the variant-specific estimates for lifetime smoking (Figure). However, the multiplicative random-effects IVW method accounts for heterogeneity and provides valid estimates under the assumption of balanced pleiotropy.^40^ There was no indication of directional (unbalanced) pleiotropy using the MR-Egger method. The MR-PRESSO test detected no outliers for advanced AMD or nAMD and 1 outlier for GA, with no significant difference in the estimates before and after adjusting for the outlier (eTable 13 in Supplement 1). The age at which smoking was initiated was not found to be associated with risk of advanced AMD and its subtypes (Figure; eFigure 4 and eTable 13 in Supplement 1).

### Association of Alcohol Consumption With Risk of Advanced AMD

We found no association between increased genetically predicted alcohol consumption and risk of advanced AMD using IVW (OR, 1.57; 95% CI, 1.03-2.40; *P* = .04), MVMR (OR, 1.65; 95% CI, 1.21-2.26; *P* = .002), weighted median (OR, 2.04; 95% CI, 1.23-3.39; *P* = .006), or MR-PRESSO (OR, 1.47; 95% CI, 1.02-2.10; *P* = .04) (Figure; eFigure 5 and eTable 13 in Supplement 1). Examination of advanced AMD subtypes showed a strong association between higher alcohol intake and GA (IVW: OR, 2.70; 95% CI, 1.48-4.94; *P* = .001; MVMR: OR, 2.83; 95% CI, 1.64-4.88; *P* < .001; and MR-PRESSO: OR, 2.50; 95% CI, 1.42-4.42; *P* = .002), but not nAMD (Figure; eTable 13 in Supplement 1). There was evidence of heterogeneity (Figure), but this was accounted for under the multiplicative random-effects IVW method.^40^ There was no indication of directional (unbalanced) pleiotropy using MR-Egger. The MR-PRESSO test detected 3 outliers for advanced AMD—1 for GA and 2 for nAMD—but there was no significant difference between the estimates before and after correction for outliers (eTable 13 in Supplement 1).

### Association of BMI, Blood Pressure, and Glycemic Risk Factors With Risk of Advanced AMD

There was no evidence that BMI, blood pressure, type 2 diabetes, HbA_1c_, fasting glucose level, or fasting insulin level had a causal association with the risk of AMD using MR (Figure; eFigures 6-12 and eTable 13 in Supplement 1).

## Discussion

### Main Findings

This study used an MR framework to explore potential causal associations between the risk of advanced AMD and the following modifiable risk factors: smoking, alcohol consumption, BMI, blood pressure, and glycemic traits. We found genetic evidence supporting a potential causal association between smoking initiation and advanced AMD risk consistent with previous observational studies. This association was stronger for nAMD than for GA. Similar results were found for lifetime smoking behavior. Additionally, smoking cessation was associated with a decreased risk of advanced AMD, specifically nAMD, compared with persistent smoking. We also found suggestive evidence for a possible causal association between increased alcohol consumption and risk of advanced AMD that was likely driven by a strong association with GA. There was insufficient evidence to suggest a potential causal association with the other exposures, namely BMI, blood pressure, or glycemic risk factors, on advanced AMD risk.

### Results in Context With the Published Literature

Conventional observational studies have consistently implicated smoking as a risk factor for developing AMD (OR, 1.7-4.6),^10,11,41,42,43^ with 1 study reporting the risk of AMD reduced to baseline 20 years after smoking cessation.^44^ Using MR techniques, we found genetic evidence to support that smoking initiation and lifetime smoking behavior may be causally associated with risk of advanced AMD and that smoking cessation is protective. Several theories have been proposed to explain the association between smoking and AMD. Oxidative stress is thought to play a major role in AMD pathogenesis.^45^ Smoking is known to decrease levels of antioxidants,^46^ resulting in the disruption of the retinal pigment epithelium barrier and leading to the formation of drusen and neovascularization.^47,48,49,50^ Smoking can also upregulate endothelial smooth muscle cell proliferation, leading to choroidal neovascularization.^51^ Atherosclerosis or vasoconstriction secondary to smoking could cause hypoxic conditions in the retina,^52^ stimulating production of vascular endothelial growth factor and resulting in retinal endothelial cell proliferation and neovascularization.^53^ Smoking has also been found to be associated with the production of inflammatory mediators and activation of the complement cascade involved in the pathogenesis of AMD.^54,55,56^ While these hypotheses may explain how smoking can cause AMD generally, it has been unclear whether and how smoking affects GA and nAMD differently. Our finding that smoking behavior has a greater potential association with nAMD compared with GA may be a reflection of how the pathogenesis of nAMD diverges from what is assumed to be the underlying default pathway of early AMD to GA.^57,58^ Further investigation is required to provide insights into how these pathways differ.

The evidence in the literature for an association between alcohol consumption and AMD risk is less robust than for smoking. While high alcohol intake has been reported to be associated with an increased risk of AMD, a systematic review concluded that residual confounding effects from smoking could not be ruled out.^13,59,60^ One study has conversely reported a protective effect with moderate alcohol consumption.^61^ Our results provide genetic evidence of a potential causal association between increased weekly alcohol consumption and risk of GA. While there are no studies, to our knowledge, looking at the role of alcohol consumption in the pathophysiology of GA specifically, the mechanism of alcohol as a risk factor for AMD is thought to be related to oxidative damage. Alcohol depletes antioxidant levels and results in the production of reactive oxygen species.^62,63^ Further studies are required to investigate why increased alcohol intake appears to have a causal association with GA but not with nAMD.

The effects of BMI and blood pressure on AMD are uncertain. Some observational studies have found increased BMI and blood pressure to be associated with increased AMD risk, whereas others showed no association.^17,26^ A meta-analysis of cohort studies found an increased risk of advanced AMD in individuals with obesity.^17^ One cohort study found antihypertensive medication to be associated with increased AMD risk.^64^ However, cohort studies are prone to confounding, and meta-analyses are prone to publication bias. Our MR estimates did not indicate potential causal associations for either BMI or blood pressure traits with risk of advanced AMD.

Reports on the association between diabetes and AMD are conflicting. A recent meta-analysis found that diabetes was associated with increased risk of AMD.^25^ However, the authors stated that it was not possible to rule out underlying confounders, as most of the included studies only adjusted for age and sex. Other studies have reported a protective effect for diabetic retinopathy on AMD risk.^65^ While both AMD and diabetic retinopathy are inflammatory retinal conditions, AMD is characterized by alterations in the outer blood-retinal barrier (BRB) and diabetic retinopathy by changes in the inner BRB. Damage to the inner BRB in diabetes is associated with upregulation of outer BRB activity,^66^ which could potentially provide a protective mechanism for AMD. The MR estimates for the 4 glycemic traits analyzed in this study did not reach statistical significance.

### Implications for Clinical Practice and Health Policy

This study provides genetic evidence that smoking initiation and lifetime smoking behavior are potential causal risk factors for advanced AMD, and that stopping smoking may be protective against the risk of advanced AMD. The detrimental effects of smoking on multiple conditions, such as cardiovascular disease, cancers, and chronic obstructive pulmonary disease, are well known. Public health campaigns should disseminate information that smoking can also lead to blindness as an additional deterrent against smoking. We also found genetic evidence that increased alcohol consumption has a potential causal association with GA risk. Here again, public health messages and clinical advice regarding the harms of excessive alcohol intake should include the risk of blindness, especially given that there are currently no effective treatments for GA. Given the deterioration in quality of life and the cost to health and social care systems of managing advanced AMD, increased funding should be allocated to smoking cessation and alcohol reduction programs to minimize the health burden of advanced AMD.

### Strengths and Limitations

This study has several strengths. The use of MR mitigates bias from reverse causation and confounding that can affect findings from observational studies. The 2-sample MR approach increases statistical power as it allows independent large GWAS summary data sets to be used for both exposures and outcomes.^67,68^ The use of multiple genetic variants as instruments for each of the risk factors enables SNVs across the whole genome to be used to assess the association of each of the modifiable exposures with risk of advanced AMD. This allowed us to perform sensitivity analyses to detect and correct for directional pleiotropy. Most of the participants in the GWAS data sets were of European descent, thus minimizing bias due to population stratification.

This study also has several limitations. While we used summary statistics from the largest known advanced AMD GWAS to date, the number of advanced AMD cases, especially for GA, was still relatively small compared with other outcomes used in MR studies, such as cardiovascular disease. This might have resulted in insufficient statistical power to detect associations with some of the exposures analyzed. Additionally, effect sizes from MR analyses should be interpreted with caution. This is because the MR estimate is better interpreted as a test statistic for a causal hypothesis rather than the expected impact of a clinical intervention at a specific point in time.^69,70,71^ Another potential limitation in MR analyses is directional horizontal pleiotropy, which can occur if genetic variants affect advanced AMD independently of the risk factors investigated. To identify and adjust for pleiotropy, we performed sensitivity analyses using MR-Egger, weighted median, MVMR, and MR-PRESSO methods (eFigures 1-12 in Supplement 1). These tests can provide reliable inferences when some genetic variants are pleiotropic.^72,73,74^ There was no evidence for directional pleiotropy using these tests. However, it is not possible to completely rule out the presence of residual pleiotropy. Additionally, we were unable to test the nonlinear association for nonbinary exposures, such as alcohol consumption, blood pressure, BMI, and quantitative glycemic traits, with advanced AMD risk, as MR analysis assumes a linear association between exposure and outcome.

## Conclusions

We found genetic evidence that increased alcohol consumption has a potential causal association with risk of GA. We also present genetic evidence that smoking initiation and lifetime smoking behavior may be casually associated with risk of advanced AMD, while smoking cessation results in a lower risk of advanced AMD than persistent smoking. These associations were stronger for nAMD than for GA. To reduce the prevalence of advanced AMD in aging populations, public health campaigns and programs to support smoking abstention, smoking cessation, and reduced alcohol intake should incorporate the evidence that these activities can lead to blindness. The finding that smoking and alcohol have differential effects on nAMD and GA may prompt future studies examining the different pathologies of these 2 forms of advanced AMD.

## Notes

Supplement 1 eMethods. Study design, data sources, SNV selection, and statistical analysis eTable 1. Smoking initiation exposure SNVs and their association with advanced AMD eTable 2. Smoking cessation exposure SNVs and their association with advanced AMD eTable 3. Lifetime smoking exposure SNVs and their association with advanced AMD eTable 4. Age at smoking initiation exposure SNVs and their association with advanced AMD eTable 5. Alcohol intake per week exposure SNVs and their association with advanced AMD eTable 6. Body mass index exposure SNVs and their association with advanced AMD eTable 7. Systolic blood pressure exposure SNVs and their association with advanced AMD eTable 8. Diastolic blood pressure exposure SNVs and their association with advanced AMD eTable 9. Type 2 diabetes exposure SNVs and their association with advanced AMD eTable10. HbA_1c_ exposure SNVs and their association with advanced AMD eTable 11. Fasting glucose exposure SNVs and their association with advanced AMD eTable 12. Fasting insulin exposure SNVs and their association with advanced AMD eTable 13. Mendelian randomization analysis of modifiable risk factors for advanced AMD, geographic atrophy, and neovascular AMD eFigure 1. Scatter plot of SNV-smoking initiation and SNV-AMD association estimates for the smoking initiation instrument eFigure 2. Scatter plot of SNV-smoking cessation and SNV-AMD association estimates for the smoking cessation instrument eFigure 3. Scatter plot of SNV-lifetime smoking and SNV-AMD association estimates for the lifetime smoking instrument eFigure 4. Scatter plot of SNV-age at smoking initiation and SNV-AMD association estimates for the age at smoking initiation instrument eFigure 5. Scatter plot of SNV-alcoholic drinks per week and SNV-AMD association estimates for the alcohol intake per week instrument eFigure 6. Scatter plot of SNV-BMI and SNV-AMD association estimates for the body mass index instrument eFigure 7. Scatter plot of SNV-systolic blood pressure and SNV-AMD association estimates for the systolic blood pressure instrument eFigure 8. Scatter plot of SNV-diastolic blood pressure and SNV-AMD association estimates for the diastolic blood pressure instrument eFigure 9. Scatter plot of SNV–type 2 diabetes and SNV-AMD association estimates for the type 2 diabetes instrument eFigure 10. Scatter plot of SNV-HbA_1c_ and SNV-AMD association estimates for the HbA_1c_ instrument eFigure 11. Scatter plot of SNV-fasting glucose and SNV-AMD association estimates for the fasting glucose instrument eFigure12. Scatter plot of SNV-fasting insulin and SNV-AMD association estimates for the fasting insulin instrument eReferences

Supplement 2. Nonauthor collaborators

## References

[1] Li JQ, Welchowski T, Schmid M, Mauschitz MM, Holz FG, Finger RP. Prevalence and incidence of age-related macular degeneration in Europe: a systematic review and meta-analysis. Br J Ophthalmol. 2020;104(8):1077-1084. doi:10.1136/bjophthalmol-2019-314422 31712255

[2] Pennington KL, DeAngelis MM. Epidemiology of age-related macular degeneration (AMD): associations with cardiovascular disease phenotypes and lipid factors. Eye Vis (Lond). 2016;3:34. doi:10.1186/s40662-016-0063-5 28032115 PMC5178091

[3] Mitchell P, Liew G, Gopinath B, Wong TY. Age-related macular degeneration. Lancet. 2018;392(10153):1147-1159. doi:10.1016/S0140-6736(18)31550-2 30303083

[4] Wong WL, Su X, Li X, . Global prevalence of age-related macular degeneration and disease burden projection for 2020 and 2040: a systematic review and meta-analysis. Lancet Glob Health. 2014;2(2):e106-e116. doi:10.1016/S2214-109X(13)70145-1 25104651

[5] Resnikoff S, Pascolini D, Etya’ale D, . Global data on visual impairment in the year 2002. Bull World Health Organ. 2004;82(11):844-851. 15640920 PMC2623053

[6] Ferris FL III, Wilkinson CP, Bird A, ; Beckman Initiative for Macular Research Classification Committee. Clinical classification of age-related macular degeneration. Ophthalmology. 2013;120(4):844-851. doi:10.1016/j.ophtha.2012.10.036 23332590 PMC11551519

[7] Finger RP, Daien V, Eldem BM, . Anti-vascular endothelial growth factor in neovascular age-related macular degeneration—a systematic review of the impact of anti-VEGF on patient outcomes and healthcare systems. BMC Ophthalmol. 2020;20(1):294. doi:10.1186/s12886-020-01554-2 32680477 PMC7368708

[8] Rofagha S, Bhisitkul RB, Boyer DS, Sadda SR, Zhang K; SEVEN-UP Study Group. Seven-year outcomes in ranibizumab-treated patients in ANCHOR, MARINA, and HORIZON: a multicenter cohort study (SEVEN-UP). Ophthalmology. 2013;120(11):2292-2299. doi:10.1016/j.ophtha.2013.03.046 23642856

[9] Lanzetta P, Loewenstein A; Vision Academy Steering Committee. Fundamental principles of an anti-VEGF treatment regimen: optimal application of intravitreal anti-vascular endothelial growth factor therapy of macular diseases. Graefes Arch Clin Exp Ophthalmol. 2017;255(7):1259-1273. doi:10.1007/s00417-017-3647-4 28527040 PMC5486551

[10] Smith W, Assink J, Klein R, . Risk factors for age-related macular degeneration: pooled findings from three continents. Ophthalmology. 2001;108(4):697-704. doi:10.1016/S0161-6420(00)00580-7 11297486

[11] Mitchell P, Wang JJ, Smith W, Leeder SR. Smoking and the 5-year incidence of age-related maculopathy: the Blue Mountains Eye Study. Arch Ophthalmol. 2002;120(10):1357-1363. doi:10.1001/archopht.120.10.1357 12365915

[12] Lambert NG, ElShelmani H, Singh MK, . Risk factors and biomarkers of age-related macular degeneration. Prog Retin Eye Res. 2016;54:64-102. doi:10.1016/j.preteyeres.2016.04.003 27156982 PMC4992630

[13] Adams MK, Chong EW, Williamson E, . 20/20—Alcohol and age-related macular degeneration: the Melbourne Collaborative Cohort Study. Am J Epidemiol. 2012;176(4):289-298. doi:10.1093/aje/kws004 22847604

[14] Boekhoorn SS, Vingerling JR, Hofman A, de Jong PT. Alcohol consumption and risk of aging macula disorder in a general population: the Rotterdam Study. Arch Ophthalmol. 2008;126(6):834-839. doi:10.1001/archopht.126.6.834 18541849

[15] Fraser-Bell S, Wu J, Klein R, Azen SP, Varma R. Smoking, alcohol intake, estrogen use, and age-related macular degeneration in Latinos: the Los Angeles Latino Eye Study. Am J Ophthalmol. 2006;141(1):79-87. doi:10.1016/j.ajo.2005.08.024 16386980

[16] Knudtson MD, Klein R, Klein BE. Alcohol consumption and the 15-year cumulative incidence of age-related macular degeneration. Am J Ophthalmol. 2007;143(6):1026-1029. doi:10.1016/j.ajo.2007.01.036 17524768 PMC1950733

[17] Zhang QY, Tie LJ, Wu SS, . Overweight, obesity, and risk of age-related macular degeneration. Invest Ophthalmol Vis Sci. 2016;57(3):1276-1283. doi:10.1167/iovs.15-18637 26990164

[18] Howard KP, Klein BE, Lee KE, Klein R. Measures of body shape and adiposity as related to incidence of age-related eye diseases: observations from the Beaver Dam Eye Study. Invest Ophthalmol Vis Sci. 2014;55(4):2592-2598. doi:10.1167/iovs.15-18637 24667857 PMC3995678

[19] Chakravarthy U, Wong TY, Fletcher A, . Clinical risk factors for age-related macular degeneration: a systematic review and meta-analysis. BMC Ophthalmol. 2010;10:31. doi:10.1186/1471-2415-10-31 21144031 PMC3009619

[20] Katsi VK, Marketou ME, Vrachatis DA, . Essential hypertension in the pathogenesis of age-related macular degeneration: a review of the current evidence. J Hypertens. 2015;33(12):2382-2388. doi:10.1097/HJH.0000000000000766 26536087

[21] Ghaem Maralani H, Tai BC, Wong TY, . Metabolic syndrome and risk of age-related macular degeneration. Retina. 2015;35(3):459-466. doi:10.1097/IAE.0000000000000338 25207946

[22] Cougnard-Grégoire A, Delyfer MN, Korobelnik JF, . Long-term blood pressure and age-related macular degeneration: the ALIENOR study. Invest Ophthalmol Vis Sci. 2013;54(3):1905-1912. doi:10.1167/iovs.12-10192 23404120

[23] Chakravarthy U, Bailey CC, Scanlon PH, . Progression from early/intermediate to advanced forms of age-related macular degeneration in a large UK cohort: rates and risk factors. Ophthalmol Retina. 2020;4(7):662-672. doi:10.1016/j.oret.2020.01.012 32144084

[24] Bikbov MM, Zainullin RM, Gilmanshin TR, . Prevalence and associated factors of age-related macular degeneration in a Russian population: the Ural Eye and Medical Study. Am J Ophthalmol. 2020;210:146-157. doi:10.1016/j.ajo.2019.10.004 31606441

[25] Chen X, Rong SS, Xu Q, . Diabetes mellitus and risk of age-related macular degeneration: a systematic review and meta-analysis. PLoS One. 2014;9(9):e108196. doi:10.1371/journal.pone.0108196 25238063 PMC4169602

[26] Heesterbeek TJ, Lorés-Motta L, Hoyng CB, Lechanteur YTE, den Hollander AI. Risk factors for progression of age-related macular degeneration. Ophthalmic Physiol Opt. 2020;40(2):140-170. doi:10.1111/opo.12675 32100327 PMC7155063

[27] Colijn JM, den Hollander AI, Demirkan A, ; European Eye Epidemiology Consortium; EYE-RISK Consortium. Increased high-density lipoprotein levels associated with age-related macular degeneration: evidence from the EYE-RISK and European Eye Epidemiology Consortia. Ophthalmology. 2019;126(3):393-406. doi:10.1016/j.ophtha.2018.09.045 30315903

[28] Age-Related Eye Disease Study Research Group. A randomized, placebo-controlled, clinical trial of high-dose supplementation with vitamins C and E, beta carotene, and zinc for age-related macular degeneration and vision loss: AREDS report no. 8. Arch Ophthalmol. 2001;119(10):1417-1436. doi:10.1001/archopht.119.10.1417 11594942 PMC1462955

[29] Age-Related Eye Disease Study 2 Research Group. Lutein + zeaxanthin and omega-3 fatty acids for age-related macular degeneration: the Age-Related Eye Disease Study 2 (AREDS2) randomized clinical trial. JAMA. 2013;309(19):2005-2015. doi:10.1001/jama.2013.4997 23644932

[30] Chew EY, Clemons TE, Sangiovanni JP, ; Age-Related Eye Disease Study 2 (AREDS2) Research Group. Secondary analyses of the effects of lutein/zeaxanthin on age-related macular degeneration progression: AREDS2 report no. 3. JAMA Ophthalmol. 2014;132(2):142-149. doi:10.1001/jamaophthalmol.2013.7376 24310343 PMC4636082

[31] Smith GD, Timpson N, Ebrahim S. Strengthening causal inference in cardiovascular epidemiology through mendelian randomization. Ann Med. 2008;40(7):524-541. doi:10.1080/07853890802010709 18608114

[32] Hingorani A, Humphries S. Nature’s randomised trials. Lancet. 2005;366(9501):1906-1908. doi:10.1016/S0140-6736(05)67767-7 16325682

[33] Burgess S, Davey Smith G. Mendelian randomization implicates high-density lipoprotein cholesterol-associated mechanisms in etiology of age-related macular degeneration. Ophthalmology. 2017;124(8):1165-1174. doi:10.1016/j.ophtha.2017.03.042 28456421 PMC5526457

[34] Fan Q, Maranville JC, Fritsche L, . HDL-cholesterol levels and risk of age-related macular degeneration: a multiethnic genetic study using mendelian randomization. Int J Epidemiol. 2017;46(6):1891-1902. doi:10.1093/ije/dyx189 29025108 PMC5837540

[35] Fritsche LG, Igl W, Bailey JN, . A large genome-wide association study of age-related macular degeneration highlights contributions of rare and common variants. Nat Genet. 2016;48(2):134-143. doi:10.1038/ng.3448 26691988 PMC4745342

[36] Bowden J, Davey Smith G, Haycock PC, Burgess S. Consistent estimation in mendelian randomization with some invalid instruments using a weighted median estimator. Genet Epidemiol. 2016;40(4):304-314. doi:10.1002/gepi.21965 27061298 PMC4849733

[37] Bowden J, Davey Smith G, Burgess S. Mendelian randomization with invalid instruments: effect estimation and bias detection through Egger regression. Int J Epidemiol. 2015;44(2):512-525. doi:10.1093/ije/dyv080 26050253 PMC4469799

[38] Verbanck M, Chen CY, Neale B, Do R. Detection of widespread horizontal pleiotropy in causal relationships inferred from mendelian randomization between complex traits and diseases. Nat Genet. 2018;50(5):693-698. doi:10.1038/s41588-018-0099-7 29686387 PMC6083837

[39] Wootton RE, Richmond RC, Stuijfzand BG, . Evidence for causal effects of lifetime smoking on risk for depression and schizophrenia: a mendelian randomisation study. Psychol Med. 2020;50(14):2435-2443. doi:10.1017/S0033291719002678 31689377 PMC7610182

[40] Bowden J, Holmes MV. Meta-analysis and mendelian randomization: a review. Res Synth Methods. 2019;10(4):486-496. Published online April 23, 2019. doi:10.1002/jrsm.1346 30861319 PMC6973275

[41] Seddon JM, Silver RE, Kwong M, Rosner B. Risk prediction for progression of macular degeneration: 10 common and rare genetic variants, demographic, environmental, and macular covariates. Invest Ophthalmol Vis Sci. 2015;56(4):2192-2202. doi:10.1167/iovs.14-15841 25655794 PMC4405097

[42] Wang JJ, Rochtchina E, Smith W, . Combined effects of complement factor H genotypes, fish consumption, and inflammatory markers on long-term risk for age-related macular degeneration in a cohort. Am J Epidemiol. 2009;169(5):633-641. doi:10.1093/aje/kwn358 19074778 PMC2732972

[43] Jonasson F, Fisher DE, Eiriksdottir G, . Five-year incidence, progression, and risk factors for age-related macular degeneration: the age, gene/environment susceptibility study. Ophthalmology. 2014;121(9):1766-1772. doi:10.1016/j.ophtha.2014.03.013 24768241 PMC4145014

[44] Vingerling JR, Hofman A, Grobbee DE, de Jong PT. Age-related macular degeneration and smoking. the Rotterdam Study. Arch Ophthalmol. 1996;114(10):1193-1196. doi:10.1001/archopht.1996.01100140393005 8859077

[45] Khandhadia S, Lotery A. Oxidation and age-related macular degeneration: insights from molecular biology. Expert Rev Mol Med. 2010;12:e34. doi:10.1017/S146239941000164X 20959033

[46] Alberg A. The influence of cigarette smoking on circulating concentrations of antioxidant micronutrients. Toxicology. 2002;180(2):121-137. doi:10.1016/S0300-483X(02)00386-4 12324189

[47] Chang MA, Bressler SB, Munoz B, West SK. Racial differences and other risk factors for incidence and progression of age-related macular degeneration: Salisbury Eye Evaluation (SEE) project. Invest Ophthalmol Vis Sci. 2008;49(6):2395-2402. doi:10.1167/iovs.07-1584 18263809

[48] Khan JC, Thurlby DA, Shahid H, ; Genetic Factors in AMD Study. Smoking and age related macular degeneration: the number of pack years of cigarette smoking is a major determinant of risk for both geographic atrophy and choroidal neovascularisation. Br J Ophthalmol. 2006;90(1):75-80. doi:10.1136/bjo.2005.073643 16361672 PMC1856879

[49] Thornton J, Edwards R, Mitchell P, Harrison RA, Buchan I, Kelly SP. Smoking and age-related macular degeneration: a review of association. Eye (Lond). 2005;19(9):935-944. doi:10.1038/sj.eye.6701978 16151432

[50] Ciulla TA, Harris A, Martin BJ. Ocular perfusion and age-related macular degeneration. Acta Ophthalmol Scand. 2001;79(2):108-115. doi:10.1034/j.1600-0420.2001.079002108.x 11284745

[51] Suñer IJ, Espinosa-Heidmann DG, Marin-Castano ME, Hernandez EP, Pereira-Simon S, Cousins SW. Nicotine increases size and severity of experimental choroidal neovascularization. Invest Ophthalmol Vis Sci. 2004;45(1):311-317. doi:10.1167/iovs.03-0733 14691189

[52] Akishima S, Matsushita S, Sato F, . Cigarette-smoke-induced vasoconstriction of peripheral arteries: evaluation by synchrotron radiation microangiography. Circ J. 2007;71(3):418-422. doi:10.1253/circj.71.418 17322645

[53] Aiello LP, Northrup JM, Keyt BA, Takagi H, Iwamoto MA. Hypoxic regulation of vascular endothelial growth factor in retinal cells. Arch Ophthalmol. 1995;113(12):1538-1544. doi:10.1001/archopht.1995.01100120068012 7487623

[54] Kunchithapautham K, Atkinson C, Rohrer B. Smoke exposure causes endoplasmic reticulum stress and lipid accumulation in retinal pigment epithelium through oxidative stress and complement activation. J Biol Chem. 2014;289(21):14534-14546. doi:10.1074/jbc.M114.564674 24711457 PMC4031511

[55] Wang L, Kondo N, Cano M, . Nrf2 signaling modulates cigarette smoke-induced complement activation in retinal pigmented epithelial cells. Free Radic Biol Med. 2014;70:155-166. doi:10.1016/j.freeradbiomed.2014.01.015 24440594 PMC4006310

[56] Gibson J, Hakobyan S, Cree AJ, . Variation in complement component C1 inhibitor in age-related macular degeneration. Immunobiology. 2012;217(2):251-255. doi:10.1016/j.imbio.2011.07.015 21852020

[57] Bird AC. Therapeutic targets in age-related macular disease. J Clin Invest. 2010;120(9):3033-3041. doi:10.1172/JCI42437 20811159 PMC2929720

[58] Ambati J, Atkinson JP, Gelfand BD. Immunology of age-related macular degeneration. Nat Rev Immunol. 2013;13(6):438-451. doi:10.1038/nri3459 23702979 PMC3941009

[59] Chong EW, Kreis AJ, Wong TY, Simpson JA, Guymer RH. Alcohol consumption and the risk of age-related macular degeneration: a systematic review and meta-analysis. Am J Ophthalmol. 2008;145(4):707-715. doi:10.1016/j.ajo.2007.12.005 18242575

[60] Dinu M, Pagliai G, Casini A, Sofi F. Food groups and risk of age-related macular degeneration: a systematic review with meta-analysis. Eur J Nutr. 2019;58(5):2123-2143. doi:10.1007/s00394-018-1771-5 29978377

[61] Obisesan TO, Hirsch R, Kosoko O, Carlson L, Parrott M. Moderate wine consumption is associated with decreased odds of developing age-related macular degeneration in NHANES-1. J Am Geriatr Soc. 1998;46(1):1-7. doi:10.1111/j.1532-5415.1998.tb01005.x 9434658

[62] Das SK, Vasudevan DM. Alcohol-induced oxidative stress. Life Sci. 2007;81(3):177-187. doi:10.1016/j.lfs.2007.05.005 17570440

[63] Cederbaum AI. Role of lipid peroxidation and oxidative stress in alcohol toxicity*.* Free Radic Biol Med. 1989;7(5):537-539. doi:10.1016/0891-5849(89)90029-4 2693222

[64] Klein R, Myers CE, Klein BE. Vasodilators, blood pressure-lowering medications, and age-related macular degeneration: the Beaver Dam Eye Study. Ophthalmology. 2014;121(8):1604-1611. doi:10.1016/j.ophtha.2014.03.005 24793737 PMC4122609

[65] Cummings M, Cunha-Vaz J. Treatment of neovascular age-related macular degeneration in patients with diabetes. Clin Ophthalmol. 2008;2(2):369-375. doi:10.2147/OPTH.S2560 19668728 PMC2693968

[66] Sander B, Larsen M, Moldow B, Lund-Andersen H. Diabetic macular edema: passive and active transport of fluorescein through the blood-retina barrier. Invest Ophthalmol Vis Sci. 2001;42(2):433-438. 11157879

[67] Burgess S, Scott RA, Timpson NJ, Davey Smith G, Thompson SG; EPIC- InterAct Consortium. Using published data in mendelian randomization: a blueprint for efficient identification of causal risk factors. Eur J Epidemiol. 2015;30(7):543-552. doi:10.1007/s10654-015-0011-z 25773750 PMC4516908

[68] Lawlor DA. Commentary: two-sample mendelian randomization: opportunities and challenges. Int J Epidemiol. 2016;45(3):908-915. doi:10.1093/ije/dyw127 27427429 PMC5005949

[69] Burgess S, Davey Smith G, Davies NM, . Guidelines for performing mendelian randomization investigations. Wellcome Open Res. 2020;4:186. doi:10.12688/wellcomeopenres.15555.2 32760811 PMC7384151

[70] Didelez V, Sheehan N. Mendelian randomization as an instrumental variable approach to causal inference. Stat Methods Med Res. 2007;16(4):309-330. doi:10.1177/0962280206077743 17715159

[71] VanderWeele TJ, Tchetgen Tchetgen EJ, Cornelis M, Kraft P. Methodological challenges in mendelian randomization. Epidemiology. 2014;25(3):427-435. doi:10.1097/EDE.0000000000000081 24681576 PMC3981897

[72] Davies NM, Holmes MV, Davey Smith G. Reading mendelian randomisation studies: a guide, glossary, and checklist for clinicians. BMJ. 2018;362:k601. doi:10.1136/bmj.k601 30002074 PMC6041728

[73] Burgess S, Bowden J, Fall T, Ingelsson E, Thompson SG. Sensitivity analyses for robust causal inference from mendelian randomization analyses with multiple genetic variants. Epidemiology. 2017;28(1):30-42. doi:10.1097/EDE.0000000000000559 27749700 PMC5133381

[74] Slob EAW, Burgess S. A comparison of robust mendelian randomization methods using summary data. Genet Epidemiol. 2020;44(4):313-329. doi:10.1002/gepi.22295 32249995 PMC7317850

