# Supplementary material for: Association of Smoking, Alcohol Consumption, Blood Pressure, Body Mass Index, and Glycemic Risk Factors With Age-Related Macular Degeneration: A Mendelian Randomization Study
Source: JAMA Ophthalmol. 2021 Nov 4;139(12):1299–306. doi: 10.1001/jamaophthalmol.2021.4601 (PMC8569599; doi:10.1001/jamaophthalmol.2021.4601)
Supplement: Supplement 1 — eMethods. Study design, data sources, SNV selection, and statistical analysis eTable 1. Smoking initiation exposure SNVs and their association with advanced AMD eTable 2. Smoking cessation exposure SNVs and their association with advanced AMD eTable 3. Lifetime smoking exposure SNVs and their association with advanced AMD eTable 4. Age at smoking initiation exposure SNVs and their association with advanced AMD eTable 5. Alcohol intake per week exposure SNVs and their association with advanced AMD eTable 6. Body mass index exposure SNVs and their association with advanced AMD eTable 7. Systolic blood pressure exposure SNVs and their association with advanced AMD eTable 8. Diastolic blood pressure exposure SNVs and their association with advanced AMD eTable 9. Type 2 diabetes exposure SNVs and their association with advanced AMD eTable10. HbA1c exposure SNVs and their association with advanced AMD eTable 11. Fasting glucose exposure SNVs and their association with advanced AMD eTable 12. Fasting insulin exposure SNVs and their association with advanced AMD eTable 13. Mendelian randomization analysis of modifiable risk factors for advanced AMD, geographic atrophy, and neovascular AMD eFigure 1. Scatter plot of SNV-smoking initiation and SNV-AMD association estimates for the smoking initiation instrument eFigure 2. Scatter plot of SNV-smoking cessation and SNV-AMD association estimates for the smoking cessation instrument eFigure 3. Scatter plot of SNV-lifetime smoking and SNV-AMD association estimates for the lifetime smoking instrument eFigure 4. Scatter plot of SNV-age at smoking initiation and SNV-AMD association estimates for the age at smoking initiation instrument eFigure 5. Scatter plot of SNV-alcoholic drinks per week and SNV-AMD association estimates for the alcohol intake per week instrument eFigure 6. Scatter plot of SNV-BMI and SNV-AMD association estimates for the body mass index instrument eFigure 7. Scatter plot of SNV-systolic blood pressure and SNV-AMD [file jamaophthalmol-e214601-s001.pdf]

Supplemental Online Content

Kuan V, Warwick A, Hingorani A, et al; International AMD Genomics Consortium (IAMDGC). Association of smoking, alcohol consumption, blood pressure, body mass index, and glycemic risk factors with age-related macular degeneration: a mendelian randomization study. *JAMA Ophthalmol*. Published online November 4, 2021. doi:10.1001/jamaophthalmol.2021.4601

eMethods. Study design, data sources, SNV selection, and statistical analysis

eTable 1. Smoking initiation exposure SNVs and their association with advanced AMD

eTable 2. Smoking cessation exposure SNVs and their association with advanced AMD

eTable 3. Lifetime smoking exposure SNVs and their association with advanced AMD

eTable 4. Age at smoking initiation exposure SNVs and their association with advanced AMD

eTable 5. Alcohol intake per week exposure SNVs and their association with advanced AMD

eTable 6. Body mass index exposure SNVs and their association with advanced AMD

eTable 7. Systolic blood pressure exposure SNVs and their association with advanced AMD

eTable 8. Diastolic blood pressure exposure SNVs and their association with advanced AMD

eTable 9. Type 2 diabetes exposure SNVs and their association with advanced AMD

eTable 10. HbA1c exposure SNVs and their association with advanced AMD

eTable 11. Fasting glucose exposure SNVs and their association with advanced AMD

eTable 12. Fasting insulin exposure SNVs and their association with advanced AMD

eTable 13. Mendelian randomization analysis of modifiable risk factors for advanced AMD, geographic atrophy, and neovascular AMD

eFigure 1. Scatter plot of SNV-smoking initiation and SNV-AMD association estimates for the smoking initiation instrument

eFigure 2. Scatter plot of SNV-smoking cessation and SNV-AMD association estimates for the smoking cessation instrument

eFigure 3. Scatter plot of SNV-lifetime smoking and SNV-AMD association estimates for the lifetime smoking instrument

eFigure 4. Scatter plot of SNV-age at smoking initiation and SNV-AMD association estimates for the age at smoking initiation instrument

eFigure 5. Scatter plot of SNV-alcoholic drinks per week and SNV-AMD association estimates for the alcohol intake per week instrument

eFigure 6. Scatter plot of SNV-BMI and SNV-AMD association estimates for the body mass index instrument

eFigure 7. Scatter plot of SNV-systolic blood pressure and SNV-AMD association estimates for the systolic blood pressure instrument

eFigure 8. Scatter plot of SNV-diastolic blood pressure and SNV-AMD association estimates for the diastolic blood pressure instrument

© 2021 Kuav V et al. *JAMA Opthalmology.*

eFigure 9. Scatter plot of SNV–type 2 diabetes and SNV-AMD association estimates for the type 2 diabetes instrument
eFigure 10. Scatter plot of SNV-HbA1c and SNV-AMD association estimates for the HbA1c instrument
eFigure 11. Scatter plot of SNV-fasting glucose and SNV-AMD association estimates for the fasting glucose instrument
eFigure12. Scatter plot of SNV-fasting insulin and SNV-AMD association estimates for the fasting insulin instrument
eReferences

This supplemental material has been provided by the authors to give readers additional information about their work.

eMethods

Study Design

Two-sample Mendelian randomization (MR) allows the use of publicly available summary-level data from multiple sources, including large consortia, and is particularly useful given that genetic association studies for risk factors and disease outcomes are often conducted in different study samples. Single nucleotide variants (SNVs) strongly associated with a risk factor were selected as genetic instruments for that risk factor. The association estimates and standard errors for these SNVs were then extracted from the advanced AMD dataset, and that of its subtypes, geographic atrophy and neovascular AMD. The SNV-outcome (advanced AMD, GA and nAMD) association estimates were regressed on the SNV-exposure (smoking traits, alcohol intake, BMI, blood pressure and glycemic traits) association estimates to provide causal estimates of the exposures on the outcomes.

Data sources

Summary-level statistics for lifestyle and metabolic risk factors were obtained from the following sources: smoking initiation (ever having smoked regularly) , smoking cessation (a binary phenotype of former versus current smokers), age of smoking initiation and alcoholic drinks per week from the GWAS and Sequencing Consortium of Alcohol and Nicotine use (GSCAN)[1]; lifetime smoking (represented by an index which captures smoking status, duration, heaviness and cessation)[2] from UK Biobank (UKB)[3]; body mass index (BMI) from a meta-analysis[4] of the Genetic Investigation of ANthropometric Traits (GIANT) consortium[5] and UKB[3]; systolic blood pressure (SBP) and diastolic blood pressure (DBP) from a meta-analysis[6] of the Genetic Epidemiology Research on Adult Health and Aging (GERA) cohort[7,8], the International Consortium for Blood Pressure (ICBP)[9] and UKB[3]; type 2 diabetes mellitus from a meta-analysis[10] of the DIAbetes Genetics Replication and Meta-analysis (DIAGRAM)[11], GERA[7,8], and UKB[3] datasets; glycated hemoglobin (HbA1c) [12], fasting glucose[13] and fasting insulin[10] from the Meta-Analyses of Glucose and Insulin-related traits Consortium (MAGIC) (https://www.magicinvestigators.org).

Summary-level genetic association data for advanced AMD and its subtypes geographic atrophy and neovascular AMD were obtained from the International Age-related Macular Degeneration Genomics Consortium (IAMDGC)[14].

© 2021 Kuav V et al. *JAMA Opthalmology.*

SNV Selection

SNVs were selected as instrumental variables for the modifiable risk factors in the study if they demonstrated genome-wide significant association ($P < 5 \times 10^{-8}$) with the respective risk factors. At loci with multiple genome-wide significant SNVs and linkage disequilibrium $R^2 > 0.1$ within 10,000 kb, the SNV with the lowest p-value for each exposure trait was selected. Palindromic SNVs for which allele frequencies were unknown in the source datasets, or with minor allele frequencies between 0.4 and 0.5 were excluded. Effect alleles for exposure SNVs were harmonized with the variant alleles for the effect estimates from the IAMDGC dataset (eFigures 1-12).

Statistical Analysis

Under inverse-variance weighted (IVW) MR analysis, the association estimates of the SNVs for the outcomes were regressed on the association estimates of the instrumental variables for each of the risk factors, weighted by the inverse variances of the association estimates of the SNVs with the outcomes, with the intercept of the regression line forced through zero. The Cochran Q statistic was assessed as a measure of heterogeneity between variant-specific causal estimates, and $P<0.05$ was considered to indicate the presence of heterogeneity[15,16]. The IVW method with multiplicative random-effects was performed as it takes into account heterogeneity in the variant-specific causal estimates, and provides valid causal estimates when horizontal pleiotropy is "balanced" (i.e. when pleiotropic effects on the outcome are equally likely to be positive as negative)[15,17,18].

Sensitivity analyses to assess the robustness of the IVW results to the assumption of balanced pleiotropy were conducted using the weighted median[19], MR-Egger[20] and Mendelian randomization pleiotropy residual sum and outlier (MR-PRESSO)[21] methods. The weighted median method gives consistent estimates if over 50% of the weight in the analysis arises from valid instruments. The weighted median estimate is the median of an empirical distribution in which each instrumental variable estimate is ordered and weighted by the inverse of its variance. MR-Egger and MR-PRESSO can test and correct for directional pleiotropy. In MR-Egger, the intercept of the regression line is allowed to deviate from zero and provides an estimate of directional pleiotropy. MR-PRESSO consists of 1) the global test, which detects the presence of directional pleiotropy; 2) the outlier test, which produces a corrected causal estimate by removing pleiotropic outlying variants; and 3) the distortion test, which evaluates whether there is a significant difference in the causal estimate before and after adjusting for outliers. The outlier adjusted causal estimates from MR-PRESSO were reported if the global test

© 2021 Kuav V et al. *JAMA Opthalmology.*

was significant and pleiotropic outliers were identified. Additionally, we performed multivariable MR[22] by adjusting for genetic predisposition to smoking traits in the analysis of alcohol intake, and for genetically-predicted alcohol intake in the analysis of smoking traits.

MR estimates are presented as odds ratios (OR) of advanced AMD and its subtypes for the effect of the risk factors as follows: one standard deviation increase in the log-odds of ever having smoked regularly; one standard deviation increase in the log-odds of smoking cessation (former versus current smoking); one standard deviation increase in the lifetime smoking index; one standard deviation increase of the age at which an individual started smoking cigarettes regularly; one standard deviation increase of log-transformed alcoholic drinks per week; one standard deviation (4.8kg/m^2) increase in BMI; ten millimeters of mercury (10 mmHg) increase in systolic and diastolic pressure; one SD increase in the log-odds of having type 2 diabetes; one percentage point increase in glycated hemoglobin (HbA1c); one mmol/l increase in fasting glucose; and one log-transformed (pmol/l) increase in fasting insulin.

© 2021 Kuav V et al. *JAMA Opthalmology.*

eTable 1. Smoking initiation exposure SNVs and their association with advanced AMD. Chr = chromosome; BP = base position (build 37); EA = effect allele; NEA = non-effect allele; EAF = effect allele frequency; SE = standard error.

SNV	Chr	BP	EA	NEA	EAF	Ever smoked regularly		Advanced AMD	
						**Beta**	**SE**	**Beta**	**SE**
rs12130857	1	7791461	A	G	0.325	-0.018	0.003	0.007	0.018
rs301807	1	8484823	G	A	0.57	0.018	0.003	-0.017	0.017
rs3820277	1	18436657	T	G	0.526	-0.019	0.003	0.007	0.017
rs1889571	1	32195819	G	T	0.131	0.022	0.004	0.025	0.025
rs10914684	1	33795572	A	G	0.324	-0.016	0.003	-0.018	0.018
rs2637869	1	38757237	A	G	0.297	0.018	0.003	0.008	0.018
rs12755632	1	41776623	G	A	0.316	-0.015	0.003	0.001	0.018
rs951740	1	44011737	A	G	0.625	0.03	0.003	0.001	0.017
rs925524	1	46496709	G	A	0.71	0.016	0.003	-0.006	0.018
rs12022778	1	50603995	C	A	0.202	0.027	0.003	0.016	0.021
rs11587399	1	50861071	T	A	0.221	-0.018	0.003	-0.02	0.02
rs4912332	1	58815243	T	C	0.491	0.014	0.003	0.014	0.016
rs1022528	1	71490122	A	G	0.344	0.017	0.003	0.003	0.017
rs12740789	1	72752073	A	G	0.178	-0.028	0.003	0.034	0.022
rs10789369	1	73824909	G	A	0.615	-0.023	0.003	-0.026	0.017
rs1514176	1	74991596	A	G	0.58	-0.019	0.003	-0.021	0.017
rs10873871	1	76689019	G	A	0.207	0.017	0.003	-0.022	0.023
rs11162019	1	87913176	T	C	0.363	-0.015	0.003	0.001	0.017
rs1008078	1	91189731	T	C	0.402	0.023	0.003	0.009	0.017
rs1935571	1	96414335	G	T	0.48	-0.016	0.003	-0.007	0.017
rs12027999	1	154206358	C	T	0.12	-0.024	0.004	-0.008	0.027
rs45444697	1	155034632	G	C	0.212	0.02	0.003	0.014	0.02
rs2901785	1	174104743	A	G	0.446	-0.017	0.003	0.008	0.017
rs147052174	1	179783167	T	G	0.017	0.062	0.01	-0.018	0.065
rs35656245	1	190957480	A	G	0.276	0.016	0.003	-0.023	0.018
rs12739243	1	210302043	C	T	0.221	-0.021	0.003	-0.028	0.02
rs12563365	1	236872829	A	G	0.556	0.017	0.003	-0.001	0.017
rs876793	1	237852083	C	T	0.349	-0.018	0.003	-0.001	0.018
rs114976176	2	264621	C	A	0.352	-0.016	0.003	0.024	0.017
rs62106258	2	417167	C	T	0.047	-0.045	0.006	-0.037	0.053
rs6731872	2	624205	G	T	0.826	0.032	0.003	0.011	0.021
rs1022376	2	22067213	C	T	0.516	-0.015	0.003	0.031	0.017
rs61533748	2	22582968	C	T	0.384	0.017	0.003	0.004	0.017
rs72790288	2	29513404	A	G	0.028	-0.046	0.008	0.027	0.053
rs2710634	2	32808804	C	T	0.521	-0.018	0.003	0.007	0.017
rs62137126	2	44250149	G	A	0.121	-0.024	0.004	0.026	0.025
rs1004787	2	45159091	A	G	0.552	0.028	0.003	-0.005	0.017
rs10490159	2	51341259	T	C	0.394	0.017	0.003	-0.021	0.017
rs1518393	2	58171220	C	A	0.619	0.017	0.003	-0.025	0.017
rs17616642	2	59022210	G	A	0.247	-0.017	0.003	0.023	0.019
rs6730325	2	59315828	A	G	0.61	-0.015	0.003	-0.037	0.017
rs2539706	2	59819545	A	G	0.53	0.016	0.003	-0.003	0.017
rs1863161	2	60139524	A	G	0.561	0.015	0.003	-0.036	0.017
rs359247	2	60477052	T	A	0.639	0.022	0.003	-0.006	0.017
rs62180324	2	63416606	A	G	0.212	-0.02	0.003	0.011	0.02
rs6750107	2	80748807	A	G	0.387	0.015	0.003	-0.001	0.017
rs12714017	2	80999398	C	T	0.511	0.015	0.003	-0.001	0.017
rs56208390	2	83247997	G	A	0.123	0.022	0.004	0.036	0.026
rs11692435	2	98275354	A	G	0.085	0.025	0.005	0.023	0.029
rs13392222	2	100672408	C	A	0.139	-0.023	0.004	0.001	0.024

© 2021 Kuav V et al. *JAMA Opthalmology.*

rs1901477	2	104126983	G	A	0.511	0.03	0.003	-0.004	0.016
rs11889814	2	104432494	C	A	0.128	-0.021	0.004	-0.013	0.025
rs3811038	2	113240183	C	T	0.279	0.019	0.003	-0.011	0.02
rs75210106	2	113246436	T	C	0.177	-0.019	0.003	-0.042	0.022
rs34399632	2	137571174	G	A	0.232	0.019	0.003	0.004	0.02
rs74697736	2	145412271	A	G	0.287	0.022	0.003	0.016	0.019
rs6756212	2	146140132	T	C	0.535	-0.034	0.003	-0.031	0.017
rs16826827	2	147825689	C	T	0.124	-0.022	0.004	-0.013	0.026
rs1445649	2	155682556	C	T	0.538	0.021	0.003	-0.019	0.016
rs1722666	2	161816880	T	C	0.732	0.016	0.003	0.002	0.019
rs11678980	2	162101261	A	G	0.45	0.018	0.003	0.004	0.018
rs12474587	2	162802993	T	G	0.429	0.024	0.003	0.004	0.017
rs357304	2	164862639	C	T	0.727	0.017	0.003	0.026	0.019
rs13007361	2	166250244	A	G	0.208	0.018	0.003	-0.002	0.02
rs7600835	2	172521827	A	G	0.342	-0.015	0.003	0.002	0.019
rs6750529	2	182027603	T	C	0.744	0.02	0.003	0.001	0.019
rs17229285	2	199523122	T	C	0.505	-0.015	0.003	0.015	0.017
rs3115418	2	200936399	C	T	0.454	-0.014	0.003	-0.018	0.016
rs62193862	2	202843875	A	G	0.1	0.024	0.004	0	0.035
rs4674916	2	225365635	A	C	0.328	-0.018	0.003	-0.032	0.017
rs4674993	2	226332033	G	A	0.2	-0.024	0.003	0.003	0.021
rs11713899	3	2365026	C	A	0.171	0.019	0.003	0.044	0.022
rs748832	3	16851202	G	A	0.371	0.017	0.003	0	0.017
rs10446419	3	25725501	G	A	0.207	-0.02	0.003	-0.01	0.021
rs13319205	3	47800216	A	T	0.29	0.017	0.003	-0.013	0.018
rs3172494	3	48731487	T	G	0.115	-0.029	0.004	-0.006	0.029
rs2526390	3	50192760	T	C	0.334	0.02	0.003	0.001	0.018
rs2276825	3	52886605	C	T	0.245	0.019	0.003	-0.003	0.019
rs2306866	3	53766212	T	A	0.614	-0.017	0.003	-0.012	0.018
rs73831818	3	55988394	G	A	0.057	0.032	0.005	0.005	0.036
rs1910236	3	59434420	A	G	0.469	0.015	0.003	0.013	0.019
rs7640107	3	59966156	T	C	0.431	-0.014	0.003	-0.033	0.017
rs2734390	3	60459291	G	A	0.372	0.015	0.003	0.016	0.017
rs221988	3	64234307	C	A	0.384	-0.015	0.003	0.01	0.019
rs62246017	3	71483084	A	G	0.323	-0.016	0.003	-0.033	0.018
rs4543050	3	74954560	T	A	0.816	0.022	0.003	0.001	0.022
rs6782116	3	77176032	T	C	0.415	-0.015	0.003	-0.035	0.017
rs13066050	3	81325861	T	C	0.208	0.019	0.003	0.012	0.02
rs12633090	3	83241365	C	G	0.182	-0.023	0.003	-0.019	0.021
rs1549979	3	85460131	T	C	0.615	-0.025	0.003	0.007	0.017
rs57153235	3	85902536	G	T	0.318	-0.019	0.003	-0.013	0.018
rs6437769	3	107997514	T	C	0.581	0.014	0.003	0.019	0.017
rs9288999	3	114147927	A	G	0.735	0.017	0.003	-0.007	0.019
rs6438436	3	117822149	T	C	0.816	0.025	0.003	0.014	0.022
rs12053870	3	118302515	G	T	0.542	0.016	0.003	-0.003	0.017
rs9826984	3	131945722	A	G	0.542	-0.014	0.003	-0.023	0.017
rs2279829	3	147106319	T	C	0.216	-0.017	0.003	-0.012	0.02
rs2319545	3	147719648	A	C	0.149	0.023	0.004	0.014	0.024
rs10935779	3	149543102	T	C	0.415	-0.014	0.003	0.015	0.017
rs963354	3	157393770	A	C	0.687	0.015	0.003	0.001	0.018
rs1714521	3	158284861	C	A	0.411	-0.016	0.003	0.005	0.017
rs1449012	3	159048333	T	C	0.463	-0.015	0.003	0.022	0.017
rs9850597	3	161761866	A	G	0.816	-0.019	0.003	-0.018	0.021
rs1187820	3	173072584	T	C	0.439	-0.014	0.003	0.024	0.018
rs16828799	3	173353739	T	G	0.156	0.02	0.004	-0.042	0.024
rs9841807	3	175718927	T	C	0.273	0.016	0.003	0.015	0.018
rs7631379	3	181409057	C	T	0.206	0.021	0.003	-0.024	0.023
rs12642744	4	28027176	T	G	0.744	-0.017	0.003	-0.003	0.019

© 2021 Kuav V et al. *JAMA Opthalmology.*

rs59537158	4	28246049	T	C	0.214	0.022	0.003	0.009	0.022
rs1389171	4	28822284	A	T	0.241	-0.017	0.003	0.024	0.019
rs55944129	4	29082156	C	T	0.267	-0.018	0.003	-0.023	0.019
rs58400863	4	31184484	A	G	0.347	-0.02	0.003	-0.007	0.018
rs7657022	4	35501032	G	A	0.489	0.018	0.003	0.022	0.017
rs112725451	4	68017710	T	C	0.169	0.026	0.003	-0.017	0.022
rs1435479	4	94550450	T	G	0.287	0.016	0.003	-0.026	0.018
rs3934797	4	112467612	A	G	0.182	-0.021	0.003	0.025	0.024
rs71602617	4	136406155	T	C	0.216	-0.018	0.003	-0.009	0.02
rs7696257	4	137474783	A	G	0.366	0.015	0.003	-0.009	0.017
rs13109980	4	140886963	A	G	0.326	-0.022	0.003	0.018	0.018
rs1116690	4	143510148	G	A	0.742	0.016	0.003	-0.007	0.019
rs13110073	4	147797913	C	T	0.395	-0.025	0.003	-0.018	0.017
rs62340589	4	176875795	C	G	0.201	0.017	0.003	0.003	0.021
rs12517438	5	30842054	G	T	0.538	0.015	0.003	0.023	0.016
rs35375873	5	43190647	C	G	0.11	-0.027	0.004	-0.026	0.03
rs71592686	5	60121271	C	T	0.274	0.021	0.003	0.028	0.019
rs2028269	5	79308315	A	G	0.399	0.016	0.003	0.018	0.017
rs6874731	5	80263865	G	T	0.484	0.015	0.003	0.007	0.017
rs6452785	5	87685500	T	C	0.474	-0.027	0.003	-0.015	0.017
rs10805858	5	88873832	T	A	0.335	0.018	0.003	-0.003	0.018
rs42417	5	94198290	T	C	0.691	0.017	0.003	0.012	0.021
rs72780746	5	103929588	C	T	0.173	-0.026	0.003	0.014	0.021
rs10060196	5	106455988	A	C	0.581	0.018	0.003	0	0.017
rs72789626	5	106825618	A	T	0.136	-0.026	0.004	0.038	0.025
rs17165769	5	107365642	G	A	0.395	0.016	0.003	0.002	0.017
rs329124	5	133865452	G	A	0.428	-0.016	0.003	-0.003	0.017
rs1385108	5	154839646	T	C	0.239	0.019	0.003	0.003	0.019
rs1173461	5	157707571	T	C	0.327	0.017	0.003	0.039	0.018
rs11956866	5	161018271	G	T	0.567	-0.015	0.003	-0.019	0.017
rs3909281	5	165096435	G	T	0.536	0.021	0.003	0.034	0.017
rs3843905	5	165427280	T	C	0.403	-0.015	0.003	0.006	0.017
rs79476395	5	166063680	G	A	0.073	0.033	0.005	0.115	0.034
rs6890961	5	166778503	T	C	0.624	-0.019	0.003	0.012	0.019
rs4044321	5	166989513	G	A	0.644	-0.023	0.003	-0.004	0.017
rs2173019	5	167614971	A	T	0.177	0.028	0.003	0.049	0.022
rs10042827	5	170299916	C	T	0.681	0.017	0.003	0.022	0.018
rs359431	5	173288534	T	C	0.56	-0.014	0.003	0.003	0.017
rs1059490	6	26171250	C	T	0.367	-0.019	0.003	0.015	0.017
rs1150668	6	28129789	G	T	0.419	-0.019	0.003	0.019	0.017
rs3218116	6	41901763	T	C	0.256	-0.02	0.003	0.002	0.019
rs160631	6	52895230	G	T	0.731	-0.017	0.003	-0.019	0.019
rs7743165	6	67521222	G	T	0.495	0.019	0.003	-0.018	0.017
rs10945141	6	69470709	A	G	0.263	0.018	0.003	0.014	0.019
rs619087	6	94175279	G	A	0.422	0.014	0.003	-0.022	0.017
rs6568832	6	97702876	A	G	0.754	0.019	0.003	0.009	0.019
rs12195240	6	98636905	A	G	0.285	0.025	0.003	-0.011	0.018
rs6936160	6	100347745	T	C	0.698	0.02	0.003	0.022	0.018
rs12530388	6	101329173	C	A	0.511	-0.018	0.003	-0.003	0.017
rs3800227	6	108994161	G	A	0.742	0.017	0.003	0.015	0.019
rs118202	6	111658371	T	G	0.812	-0.037	0.003	0.01	0.021
rs73008357	6	156431856	C	A	0.121	-0.022	0.004	-0.023	0.026
rs9331343	6	157738258	C	T	0.568	-0.014	0.003	0.006	0.026
rs1737329	6	163807748	G	C	0.742	0.017	0.003	-0.025	0.019
rs10272990	7	1703675	C	T	0.328	-0.021	0.003	0.01	0.018
rs6948707	7	1870794	G	T	0.419	0.024	0.003	-0.002	0.017
rs7809303	7	69484366	A	G	0.325	-0.021	0.003	-0.005	0.018
rs7802996	7	77771983	T	C	0.166	-0.021	0.003	-0.034	0.022

© 2021 Kuav V et al. *JAMA Opthalmology.*

rs1030015	7	78139581	T	G	0.52	0.014	0.003	-0.004	0.016
rs4727189	7	88442568	C	T	0.344	0.015	0.003	-0.006	0.017
rs76841737	7	91281409	G	C	0.103	-0.023	0.004	-0.045	0.031
rs11768481	7	96629103	A	C	0.34	-0.019	0.003	0.004	0.02
rs1799068	7	97707069	T	G	0.379	0.017	0.003	0.013	0.017
rs13437771	7	99071478	G	A	0.155	-0.027	0.004	0.046	0.023
rs11766326	7	111100585	C	T	0.506	-0.018	0.003	-0.006	0.017
rs6968380	7	114940159	A	G	0.681	-0.023	0.003	-0.026	0.018
rs10233018	7	117523709	G	A	0.516	0.025	0.003	-0.054	0.017
rs10953957	7	121954709	A	G	0.386	0.014	0.003	0.021	0.017
rs77283305	7	132593831	A	G	0.306	-0.015	0.003	0.024	0.018
rs10279261	7	133589846	A	G	0.618	-0.019	0.003	-0.004	0.017
rs1561112	7	133840652	C	T	0.413	-0.015	0.003	-0.003	0.017
rs2952251	8	10143164	G	A	0.744	0.016	0.003	-0.016	0.019
rs11783093	8	27425349	T	C	0.158	-0.047	0.003	0.007	0.023
rs7836565	8	52569449	T	C	0.718	-0.016	0.003	-0.021	0.018
rs13261666	8	59814666	T	G	0.517	-0.02	0.003	0	0.016
rs2063976	8	91096366	T	C	0.665	-0.02	0.003	0.015	0.018
rs6993429	8	92733282	A	C	0.453	-0.019	0.003	-0.017	0.017
rs6986430	8	93048104	C	T	0.222	-0.024	0.003	-0.013	0.019
rs9987376	8	93190014	G	T	0.574	-0.02	0.003	0.015	0.017
rs290601	8	115374642	T	C	0.274	0.016	0.003	-0.005	0.018
rs3847244	9	3025368	T	C	0.47	0.019	0.003	0.02	0.017
rs11791671	9	3398679	T	C	0.067	0.028	0.005	-0.07	0.038
rs7024924	9	8282399	C	T	0.174	0.019	0.003	0.012	0.022
rs7867822	9	20676454	G	A	0.673	-0.015	0.003	-0.009	0.017
rs10966092	9	23831658	C	T	0.267	-0.02	0.003	-0.037	0.019
rs4877285	9	81354129	A	G	0.668	-0.018	0.003	-0.01	0.019
rs1930371	9	81444104	T	C	0.241	-0.017	0.003	0.002	0.019
rs2378662	9	86707289	A	G	0.541	0.015	0.003	-0.005	0.017
rs1927901	9	120519111	C	T	0.553	-0.014	0.003	0.011	0.016
rs4837631	9	122061948	T	C	0.446	-0.015	0.003	-0.017	0.017
rs1759433	9	128073097	A	G	0.48	0.015	0.003	-0.01	0.016
rs34553878	9	134334588	G	A	0.111	0.025	0.004	0.048	0.029
rs7026534	9	134907263	G	T	0.704	-0.017	0.003	-0.022	0.018
rs10858334	9	137989785	G	C	0.14	0.023	0.004	0.044	0.027
rs10905461	10	8803551	C	T	0.748	-0.016	0.003	-0.014	0.019
rs1291821	10	11133823	G	A	0.534	0.014	0.003	0.005	0.017
rs11258417	10	13533053	T	C	0.391	-0.015	0.003	-0.024	0.017
rs7072776	10	22032942	G	A	0.712	-0.022	0.003	-0.028	0.019
rs2796793	10	36634124	A	G	0.452	0.014	0.003	-0.01	0.017
rs1733760	10	56698174	C	T	0.51	0.015	0.003	0.004	0.017
rs7901883	10	103186838	A	G	0.23	-0.019	0.003	-0.009	0.02
rs11594623	10	103960351	C	T	0.234	0.027	0.003	0.014	0.02
rs11191269	10	104120522	G	C	0.193	0.018	0.003	-0.032	0.021
rs28408682	10	104403310	G	A	0.6	0.017	0.003	-0.019	0.018
rs12244388	10	104640052	A	G	0.35	0.026	0.003	0.045	0.017
rs34970111	10	106078937	T	C	0.458	-0.015	0.003	-0.001	0.017
rs9787523	10	106460460	C	T	0.418	-0.016	0.003	-0.019	0.017
rs11192347	10	106929313	A	G	0.104	-0.026	0.004	0.013	0.028
rs10885480	10	115378364	C	T	0.284	-0.019	0.003	0.021	0.019
rs4752018	10	118678712	A	C	0.231	0.019	0.003	0.035	0.02
rs9423279	10	125680419	G	C	0.645	-0.019	0.003	-0.019	0.022
rs6265	11	27679916	T	C	0.188	-0.029	0.003	-0.038	0.021
rs4275621	11	28652996	G	A	0.382	-0.021	0.003	-0.021	0.017
rs62618693	11	32956492	T	C	0.043	-0.035	0.006	-0.046	0.041
rs2939756	11	41436297	A	G	0.48	-0.016	0.003	-0.021	0.017
rs1381775	11	42442826	C	T	0.712	-0.016	0.003	-0.015	0.018

© 2021 Kuav V et al. *JAMA Opthalmology.*

rs2959084	11	46078656	A	G	0.705	0.017	0.003	0.03	0.019
rs3740977	11	46393574	C	T	0.167	0.019	0.003	-0.047	0.022
rs61886926	11	64133552	T	C	0.384	-0.018	0.003	-0.003	0.017
rs61884449	11	64485193	T	C	0.149	0.02	0.004	-0.003	0.023
rs644740	11	65561468	T	C	0.457	-0.014	0.003	0.025	0.016
rs7943721	11	73309393	A	G	0.829	-0.021	0.003	-0.038	0.031
rs7929518	11	85980958	G	A	0.773	0.019	0.003	-0.01	0.019
rs586699	11	92289734	A	G	0.543	-0.015	0.003	0.028	0.016
rs76460663	11	111979741	G	C	0.041	-0.042	0.006	0.052	0.056
rs2155646	11	112912811	C	T	0.4	0.038	0.003	0.024	0.017
rs1713676	11	113660576	G	A	0.523	-0.017	0.003	-0.009	0.016
rs238896	11	113994505	A	G	0.49	-0.017	0.003	0.009	0.018
rs540860	11	121530888	G	A	0.543	0.018	0.003	-0.003	0.017
rs1944689	11	121634334	T	G	0.786	0.018	0.003	-0.011	0.02
rs1834306	11	122023187	G	A	0.579	-0.014	0.003	-0.003	0.019
rs1106363	11	131966264	T	C	0.345	0.017	0.003	0.016	0.017
rs2010921	11	132098205	A	G	0.311	0.017	0.003	-0.001	0.019
rs11057005	12	16748721	G	A	0.441	-0.016	0.003	-0.011	0.017
rs13906	12	49952394	T	C	0.109	-0.025	0.004	0.006	0.026
rs4759229	12	56474480	G	A	0.656	0.016	0.003	0.008	0.017
rs7969559	12	69655167	G	A	0.713	-0.017	0.003	-0.028	0.018
rs7134009	12	75263193	C	T	0.287	-0.016	0.003	-0.029	0.018
rs77215829	12	112618346	C	A	0.131	-0.024	0.004	-0.057	0.028
rs1109480	12	121083279	A	G	0.384	-0.017	0.003	0.028	0.018
rs11611651	12	133380790	A	G	0.087	0.027	0.005	-0.009	0.031
rs17197663	13	38172867	A	G	0.125	-0.022	0.004	-0.005	0.025
rs4264267	13	38359676	T	C	0.527	0.015	0.003	0.037	0.019
rs61959481	13	55834929	A	G	0.21	-0.02	0.003	-0.019	0.02
rs3098272	13	55931424	C	A	0.799	-0.018	0.003	-0.02	0.021
rs9538162	13	59265043	C	T	0.416	0.017	0.003	-0.001	0.017
rs1413119	13	59339281	T	C	0.396	-0.015	0.003	-0.005	0.017
rs56367474	13	59454139	T	C	0.304	-0.017	0.003	-0.002	0.019
rs55786907	13	59871584	G	A	0.162	0.019	0.003	-0.01	0.023
rs4886207	13	60705792	C	T	0.637	-0.016	0.003	0.028	0.017
rs9540731	13	66949370	T	C	0.509	-0.018	0.003	0.008	0.016
rs9545155	13	80191873	C	T	0.478	-0.016	0.003	0.005	0.016
rs1772572	13	81191176	A	C	0.324	-0.017	0.003	0.012	0.018
rs75674569	13	96823724	A	G	0.1	-0.025	0.004	-0.05	0.028
rs7333559	13	100546450	A	G	0.783	-0.023	0.003	0.001	0.021
rs1108130	13	100648356	A	T	0.212	0.024	0.003	-0.014	0.021
rs12855717	13	101252635	T	C	0.538	0.016	0.003	-0.01	0.017
rs12878369	14	28346502	A	C	0.415	0.017	0.003	0.019	0.017
rs9323328	14	58653514	G	A	0.537	-0.014	0.003	-0.009	0.016
rs1811739	14	77529375	A	G	0.248	0.018	0.003	-0.005	0.019
rs8005334	14	79563654	G	T	0.36	0.017	0.003	-0.012	0.017
rs34940743	14	80102233	G	A	0.346	0.016	0.003	0.016	0.022
rs2925128	14	98362355	T	C	0.385	0.017	0.003	-0.025	0.017
rs1381287	14	98597552	T	C	0.467	0.018	0.003	0.032	0.017
rs55913542	14	99693843	T	G	0.175	0.019	0.003	0.001	0.022
rs1435672	15	36399479	C	T	0.56	0.014	0.003	0.026	0.017
rs281296	15	47685010	A	G	0.357	0.025	0.003	0.002	0.018
rs56902655	15	63898709	G	T	0.136	-0.022	0.004	-0.03	0.024
rs2289791	15	67476952	T	G	0.247	-0.018	0.003	-0.045	0.021
rs60833441	15	74048768	G	A	0.461	-0.014	0.003	-0.021	0.017
rs62007780	15	78025464	T	G	0.416	-0.016	0.003	0.011	0.017
rs4310804	15	96858409	G	C	0.247	-0.018	0.003	0.001	0.02
rs8027457	15	99204101	C	T	0.511	0.015	0.003	0.001	0.017
rs1139897	16	720986	A	G	0.23	-0.024	0.003	0.031	0.019

© 2021 Kuav V et al. *JAMA Opthalmology.*

rs11076962	16	5811367	C	T	0.279	0.018	0.003	-0.002	0.019
rs7192140	16	10173748	C	T	0.498	-0.017	0.003	0.049	0.017
rs9922607	16	17570220	T	C	0.2	-0.022	0.003	-0.03	0.021
rs9941217	16	18050926	G	C	0.352	-0.019	0.003	0.016	0.018
rs7188873	16	24727064	G	A	0.613	0.02	0.003	-0.018	0.017
rs6497840	16	25351633	A	G	0.707	0.023	0.003	0.003	0.019
rs4785187	16	49766772	A	G	0.223	0.02	0.003	-0.012	0.02
rs8050598	16	49891964	T	C	0.254	0.019	0.003	0.029	0.023
rs12918191	16	50945156	G	A	0.243	-0.02	0.003	-0.015	0.019
rs9302604	16	69576894	G	A	0.435	0.019	0.003	0.006	0.017
rs9936784	16	72230694	G	T	0.534	0.014	0.003	0.021	0.017
rs62052916	16	72574550	T	A	0.07	-0.032	0.005	0.006	0.034
rs4788676	16	72950468	C	T	0.229	-0.018	0.003	-0.012	0.019
rs117657830	16	75766873	G	A	0.042	-0.038	0.006	-0.05	0.041
rs1050847	16	87443734	T	C	0.559	-0.015	0.003	-0.004	0.017
rs11642231	16	89608702	A	G	0.369	-0.016	0.003	-0.011	0.017
rs4790874	17	1995177	T	C	0.532	0.017	0.003	0.085	0.017
rs11078713	17	7795972	G	A	0.419	-0.015	0.003	-0.003	0.018
rs28441558	17	7803118	C	T	0.056	-0.036	0.006	-0.014	0.041
rs11651955	17	16235462	A	G	0.499	-0.014	0.003	-0.014	0.017
rs67777803	17	27323322	T	G	0.172	-0.025	0.003	-0.018	0.022
rs2344976	17	30685935	C	T	0.612	-0.015	0.003	0.014	0.017
rs3764351	17	37824339	A	G	0.657	-0.015	0.003	0.025	0.017
rs17692129	17	44793283	T	C	0.331	0.02	0.003	-0.012	0.029
rs75919030	17	50193197	C	T	0.267	-0.021	0.003	-0.014	0.019
rs2938134	17	50243397	A	C	0.673	-0.018	0.003	-0.005	0.017
rs2587507	17	77790135	C	T	0.502	-0.015	0.003	-0.017	0.016
rs34342129	18	5872472	C	T	0.509	-0.014	0.003	0.014	0.017
rs4476253	18	25253297	A	G	0.24	-0.018	0.003	-0.005	0.02
rs7505855	18	31696075	T	C	0.586	-0.017	0.003	0.003	0.017
rs8096225	18	36921851	C	A	0.703	0.016	0.003	-0.001	0.018
rs67050670	18	39297254	G	A	0.229	-0.02	0.003	-0.004	0.019
rs72898831	18	42658643	G	A	0.155	-0.024	0.004	0.01	0.024
rs8083764	18	49874515	T	G	0.306	-0.016	0.003	-0.014	0.018
rs1373178	18	49967811	G	T	0.588	-0.02	0.003	-0.017	0.017
rs62098013	18	50863861	A	G	0.365	0.018	0.003	-0.006	0.018
rs72938304	18	53661743	A	G	0.113	-0.027	0.004	-0.028	0.027
rs11872397	18	72535282	A	G	0.253	-0.017	0.003	0.047	0.02
rs71367544	18	77574374	T	C	0.203	0.021	0.003	0.043	0.021
rs76608582	19	4474725	A	C	0.049	-0.035	0.006	-0.009	0.061
rs10853981	19	4965064	A	G	0.33	0.015	0.003	0.003	0.018
rs113230003	19	18460956	A	G	0.255	-0.019	0.003	-0.015	0.019
rs8103660	19	18566395	C	T	0.354	0.016	0.003	0.02	0.017
rs117734003	19	51129745	C	G	0.067	0.03	0.005	0.077	0.042
rs1126757	19	55879872	T	C	0.473	0.014	0.003	0.022	0.016
rs6050446	20	25195509	G	A	0.971	0.054	0.008	-0.025	0.05
rs1555445	20	31175258	T	A	0.318	0.019	0.003	-0.006	0.018
rs6073075	20	42015801	A	T	0.824	-0.019	0.003	-0.025	0.023
rs910912	20	54462393	C	T	0.739	-0.017	0.003	0.011	0.019
rs6011779	20	61984317	T	C	0.806	-0.019	0.003	-0.038	0.033
rs3810496	20	62406886	C	T	0.619	0.016	0.003	0.02	0.018
rs4818005	21	40588819	A	G	0.581	-0.02	0.003	-0.029	0.017
rs139896	22	38397797	C	T	0.648	0.015	0.003	0.023	0.017
rs4822102	22	42698430	T	C	0.618	-0.017	0.003	-0.015	0.017

© 2021 Kuav V et al. *JAMA Opthalmology.*

eTable 2. Smoking cessation exposure SNVs and their association with advanced AMD. Chr = chromosome; BP = base position (build 37); EA = effect allele; NEA = non-effect allele; EAF = effect allele frequency; SE = standard error.

SNV	Chr	BP	EA	NEA	EAF	Smoking cessation		Advanced AMD	
						**Beta**	**SE**	**Beta**	**SE**
rs112187834	2	23953454	A	T	0.14	-0.033	0.006	0.019	0.023
rs7617480	3	49210732	C	A	0.773	0.033	0.005	-0.044	0.019
rs12203592	6	396321	T	C	0.176	0.029	0.005	0.000	0.044
rs707968	6	35058117	G	A	0.681	-0.023	0.004	-0.004	0.018
rs7778443	7	32314690	C	T	0.618	0.023	0.004	-0.03	0.017
rs1565735	8	27426077	A	T	0.199	0.035	0.005	0.000	0.021
rs60749569	8	42602668	T	A	0.08	0.04	0.007	-0.007	0.034
rs12378015	9	127917257	A	G	0.3	0.028	0.004	0.003	0.018
rs9409844	9	136461851	A	G	0.045	0.059	0.009	-0.107	0.039
rs3025327	9	136467344	C	G	0.107	-0.079	0.006	-0.005	0.028
rs10821523	9	136473572	C	A	0.536	-0.026	0.004	0.003	0.017
rs1611124	9	136509275	T	G	0.068	0.045	0.008	-0.014	0.033
rs7109376	11	16372431	A	T	0.279	-0.028	0.004	-0.005	0.018
rs591143	15	47647755	T	C	0.592	0.024	0.004	0.008	0.017
rs3866543	15	76629609	G	T	0.523	-0.022	0.004	-0.015	0.016
rs518425	15	78883813	G	A	0.285	0.031	0.004	-0.028	0.018
rs145580088	19	41342842	G	A	0.024	-0.091	0.013	0.167	0.084
rs56113850	19	41353107	C	T	0.567	0.058	0.004	-0.032	0.02
rs117824460	19	41371480	G	A	0.027	-0.086	0.012	0.164	0.068
rs59586387	19	41375030	G	C	0.068	-0.051	0.008	0.011	0.047
rs6011779	20	61984317	T	C	0.806	0.05	0.005	-0.038	0.033
rs6089904	20	62018289	T	A	0.047	0.064	0.009	0.02	0.052
rs9607805	22	41854446	T	C	0.725	-0.03	0.004	0.005	0.019

© 2021 Kuav V et al. *JAMA Opthalmology.*

eTable 3. Lifetime smoking exposure SNVs and their association with advanced AMD.[a] Chr = chromosome; BP = base position (build 37); EA = effect allele; NEA = non-effect allele; EAF = effect allele frequency; SE = standard error.

SNV	Chr	BP	EA	NEA	EAF	Lifetime smoking index		Advanced AMD	
						**Beta**	**SE**	**Beta**	**SE**
rs4949465	1	32178489	T	C	0.87	-0.017	0.003	-0.021	0.024
rs549845	1	44076469	G	A	0.301	0.016	0.002	0.008	0.018
rs1933270	1	49977965	T	G	0.364	0.013	0.002	0.011	0.017
rs7528604	1	66407352	G	A	0.566	0.014	0.002	0.012	0.017
rs11210229	1	73860028	A	G	0.384	0.017	0.002	0.03	0.017
rs7553348	1	75005067	G	A	0.438	0.014	0.002	0.024	0.017
rs1931263	1	96175101	G	T	0.51	-0.011	0.002	0.01	0.016
rs7519626	1	99514554	C	T	0.324	0.012	0.002	-0.033	0.018
rs9435340	1	107593201	T	A	0.344	0.012	0.002	0.06	0.017
rs10918701	1	162090536	G	A	0.372	0.012	0.002	0.006	0.017
rs2867112	2	651349	T	G	0.835	0.021	0.003	0.013	0.021
rs6741228	2	22548774	T	C	0.433	0.011	0.002	-0.004	0.017
rs62135536	2	44326028	C	T	0.968	0.035	0.006	-0.036	0.05
rs7569203	2	45154418	A	C	0.689	-0.016	0.002	0.016	0.018
rs13016665	2	57995348	C	A	0.577	-0.012	0.002	0.023	0.017
rs4671357	2	60136176	T	C	0.519	-0.014	0.002	0.022	0.017
rs359243	2	60475509	T	C	0.393	-0.013	0.002	0.008	0.017
rs62155874	2	105973094	A	G	0.873	-0.024	0.003	-0.008	0.026
rs3811038	2	113240183	T	C	0.724	-0.014	0.002	0.011	0.02
rs2890772	2	146175106	G	T	0.413	-0.02	0.002	-0.056	0.017
rs62175972	2	161362830	T	C	0.966	0.031	0.006	0.007	0.044
rs13009008	2	174043233	A	G	0.328	0.012	0.002	-0.02	0.017
rs4473348	2	182073742	A	T	0.25	-0.015	0.002	0.005	0.019
rs12623702	2	202885506	A	G	0.613	-0.014	0.002	0.000	0.018
rs6779302	3	16859710	G	T	0.633	-0.013	0.002	-0.001	0.017
rs6778080	3	49317338	T	C	0.267	0.016	0.002	0.049	0.018
rs421983	3	84892866	T	C	0.519	0.013	0.002	0.027	0.016
rs326341	3	107811142	G	A	0.525	0.014	0.002	-0.003	0.017
rs73220544	3	131074511	A	C	0.842	-0.016	0.003	0.031	0.024
rs9842947	3	157412246	C	T	0.326	-0.013	0.002	-0.001	0.018
rs624833	4	2881256	T	G	0.695	0.013	0.002	-0.005	0.018
rs61796681	4	23678196	A	T	0.912	-0.019	0.004	-0.017	0.031
rs317021	4	35418368	T	A	0.814	-0.017	0.003	-0.012	0.022
rs72678864	4	112422145	G	A	0.829	0.018	0.003	-0.035	0.026
rs17576594	4	147952241	G	A	0.724	0.016	0.002	0.026	0.018
rs11948770	5	13246336	T	C	0.768	-0.015	0.002	0.000	0.019
rs71627581	5	43161351	G	A	0.889	0.019	0.003	0.029	0.03
rs10052591	5	50812738	T	C	0.573	0.012	0.002	-0.016	0.017
rs2080870	5	60388313	A	T	0.258	0.012	0.002	0.008	0.019
rs4571506	5	87756918	C	T	0.54	0.011	0.002	0.017	0.016
rs4957528	5	106420589	A	C	0.208	-0.015	0.002	0.027	0.022
rs329120	5	133861756	C	T	0.581	0.014	0.002	0.005	0.017
rs986391	5	166993972	G	A	0.367	0.016	0.002	0.01	0.017
rs13153393	5	167604213	A	G	0.884	-0.02	0.003	-0.063	0.027
rs245774	5	170530930	A	G	0.272	-0.013	0.002	-0.029	0.019
rs6935954	6	26255451	A	G	0.421	0.014	0.002	-0.019	0.017
rs2254710	6	37477000	C	A	0.236	0.013	0.002	-0.012	0.02
rs2894808	6	52861990	T	A	0.922	-0.022	0.004	-0.032	0.03
rs12202536	6	67475273	A	G	0.513	-0.012	0.002	0.016	0.016
rs7766610	6	111707821	C	A	0.183	0.018	0.003	-0.014	0.021
rs1922018	7	3560401	C	T	0.364	0.014	0.002	0.005	0.017

© 2021 Kuav V et al. *JAMA Opthalmology.*

rs10226228	7	32315613	A	G	0.63	-0.016	0.002	-0.032	0.017
rs11768481	7	96629103	C	A	0.666	0.013	0.002	-0.004	0.02
rs6962772	7	99081730	A	G	0.846	0.016	0.003	-0.047	0.023
rs10282292	7	111092478	C	T	0.362	0.013	0.002	0.017	0.017
rs7807019	7	117543063	A	G	0.54	-0.015	0.002	0.046	0.017
rs6957896	7	132309592	C	T	0.503	-0.011	0.002	-0.009	0.017
rs4731925	7	132664757	C	T	0.316	-0.012	0.002	0.022	0.018
rs35169606	8	9604066	T	G	0.612	0.013	0.002	0.05	0.019
rs11783093	8	27425349	C	T	0.839	0.023	0.003	-0.007	0.023
rs2062882	8	91839576	G	A	0.587	-0.012	0.002	0.011	0.017
rs72674867	8	95578201	A	T	0.765	0.013	0.002	-0.007	0.019
rs4543592	9	3014254	T	C	0.52	-0.012	0.002	-0.033	0.017
rs7039819	9	82430418	G	A	0.427	0.013	0.002	0.01	0.017
rs1246265	9	86761745	T	C	0.305	-0.013	0.002	-0.009	0.018
rs13296519	9	128471924	G	T	0.606	-0.014	0.002	-0.01	0.017
rs113382419	9	136463019	C	A	0.889	-0.041	0.003	0.006	0.028
rs11255908	10	8802912	T	G	0.743	-0.015	0.002	-0.018	0.019
rs2675638	10	63576286	G	A	0.581	0.012	0.002	0.004	0.017
rs10823968	10	74738269	A	T	0.633	0.012	0.002	0.014	0.017
rs17553262	10	92912773	A	C	0.885	-0.018	0.003	-0.018	0.034
rs7077678	10	104438565	C	T	0.623	0.012	0.002	-0.015	0.017
rs12244388	10	104640052	G	A	0.661	-0.019	0.002	-0.045	0.017
rs3896224	10	106467853	A	G	0.585	0.014	0.002	0.021	0.017
rs34866095	11	16377356	A	G	0.686	-0.012	0.002	0.006	0.018
rs75742406	11	17070365	G	A	0.739	0.014	0.002	-0.029	0.019
rs17309874	11	27667236	G	A	0.74	-0.016	0.002	-0.007	0.019
rs4391802	11	28674592	A	G	0.707	0.015	0.002	0.031	0.018
rs112282219	11	46632809	G	A	0.959	-0.033	0.005	0.024	0.044
rs9919670	11	112877304	G	A	0.612	-0.022	0.002	-0.026	0.017
rs74086911	12	50015942	G	A	0.925	0.021	0.004	-0.006	0.032
rs7297175	12	56473808	T	C	0.431	-0.012	0.002	0.001	0.017
rs10879871	12	75380511	T	G	0.343	-0.014	0.002	-0.036	0.017
rs12831617	12	84758368	C	T	0.764	-0.013	0.002	-0.03	0.019
rs6562474	13	67332812	C	G	0.651	0.012	0.002	0.007	0.017
rs7333559	13	100546450	G	A	0.212	0.015	0.002	-0.001	0.021
rs860326	14	57342912	C	T	0.428	0.012	0.002	-0.02	0.017
rs7155595	14	77502546	A	C	0.674	-0.013	0.002	-0.007	0.018
rs3742365	14	104198251	T	C	0.595	-0.016	0.002	0.022	0.017
rs35175834	15	47680815	G	A	0.788	-0.024	0.002	0.021	0.02
rs28485305	15	74044197	C	T	0.631	0.012	0.002	0.028	0.017
rs8042849	15	78817929	C	T	0.342	0.028	0.002	0.026	0.017
rs8042134	15	97514404	T	G	0.541	-0.014	0.002	-0.032	0.018
rs6598539	15	99204483	T	C	0.489	-0.012	0.002	-0.001	0.017
rs11861214	16	746611	G	T	0.784	0.014	0.002	-0.032	0.02
rs12708665	16	24728227	A	G	0.285	-0.013	0.002	0.016	0.018
rs57611503	16	31165795	G	A	0.485	0.011	0.002	0.009	0.018
rs889398	16	69556715	C	T	0.588	0.013	0.002	0.002	0.017
rs60952428	16	75640521	T	C	0.909	0.019	0.003	0.001	0.029
rs1050847	16	87443734	C	T	0.426	0.011	0.002	0.004	0.017
rs369230	16	89645437	G	T	0.308	-0.013	0.002	-0.013	0.022
rs8614	17	27588806	C	A	0.817	-0.017	0.003	-0.01	0.023
rs732083	17	37834367	G	A	0.333	0.012	0.002	-0.029	0.017
rs9904288	17	47031973	T	C	0.708	0.012	0.002	0.01	0.018
rs67596067	17	50333733	G	A	0.649	-0.013	0.002	-0.008	0.019
rs12967855	18	35138245	A	G	0.331	0.012	0.002	0.03	0.018
rs62098013	18	50863861	G	A	0.64	-0.012	0.002	0.006	0.018
rs71367545	18	77576337	G	A	0.791	-0.015	0.002	-0.04	0.021
rs76608582	19	4474725	C	A	0.953	0.031	0.005	0.009	0.061

© 2021 Kuav V et al. *JAMA Opthalmology.*

rs35343344	19	18471610	C	A	0.733	0.013	0.002	0.008	0.02
rs4814873	20	19616429	C	T	0.767	0.014	0.002	0.032	0.02
rs6119897	20	31145415	G	A	0.762	-0.018	0.002	0.01	0.019
rs12481282	20	44761377	G	C	0.722	-0.013	0.002	0.028	0.019
rs348809	20	59032097	A	G	0.348	-0.012	0.002	0.019	0.017
rs6011779	20	61984317	C	T	0.191	0.028	0.003	0.038	0.033
rs147412694	21	40702786	G	A	0.85	-0.017	0.003	-0.081	0.025
rs2838834	21	46665208	C	T	0.699	-0.013	0.002	-0.022	0.018
rs136233	22	31212410	A	G	0.809	-0.014	0.003	0.011	0.021
rs202645	22	41798520	A	G	0.203	-0.015	0.002	-0.012	0.02

[a] The variants together explain 1.2% of the variation in the lifetime smoking index

© 2021 Kuav V et al. *JAMA Opthalmology.*

eTable 4. Age at smoking initiation exposure SNVs and their association with advanced AMD.[a]
Chr = chromosome; BP = base position (build 37); EA = effect allele; NEA = non-effect allele; EAF = effect allele frequency; SE = standard error.

SNV	Chr	BP	EA	NEA	EAF	Age of initiation of regular smoking		Advanced AMD	
						**Beta**	**SE**	**Beta**	**SE**
rs72853300	2	145638766	T	C	0.153	0.019	0.003	-0.037	0.027
rs11915747	3	85699040	G	C	0.354	0.02	0.003	-0.012	0.017
rs13136239	4	140908755	A	G	0.342	0.015	0.003	0.015	0.017
rs2471711	4	28589079	T	C	0.152	-0.019	0.003	0.009	0.023
rs624833	4	2881256	G	T	0.302	0.016	0.003	0.005	0.018
rs7682598	4	68000888	G	A	0.771	0.017	0.003	0.013	0.019
rs11780471	8	27344719	A	G	0.06	0.033	0.005	-0.007	0.035

[a] The variants together explain <1% of the variation in the age of initiation of regular smoking

© 2021 Kuav V et al. *JAMA Opthalmology.*

eTable 5. Alcohol intake per week exposure SNVs and their association with advanced AMD.[a]
Chr = chromosome; BP = base position (build 37); EA = effect allele; NEA = non-effect allele; EAF = effect allele frequency; SE = standard error.

SNV	Chr	BP	EA	NEA	EAF	Drinks per week		Advanced AMD	
						**Beta**	**SE**	**Beta**	**SE**
rs705687	1	4548453	G	A	0.785	-0.011	0.002	-0.011	0.02
rs58107686	1	33837334	A	C	0.328	-0.01	0.002	-0.019	0.017
rs12088813	1	66407700	C	A	0.267	-0.009	0.002	-0.021	0.019
rs5024204	1	71491890	T	A	0.278	0.01	0.002	-0.008	0.018
rs10753661	1	165119792	A	G	0.684	-0.009	0.002	0.005	0.018
rs28680958	1	173848808	A	G	0.217	-0.011	0.002	0.013	0.02
rs823114	1	205719532	A	G	0.553	0.009	0.001	-0.004	0.017
rs77165542	2	430975	T	C	0.035	-0.026	0.004	-0.02	0.066
rs1260326	2	27730940	C	T	0.601	0.021	0.001	-0.038	0.017
rs2178197	2	27860551	G	A	0.569	-0.009	0.001	0.007	0.017
rs13383034	2	45155276	T	C	0.329	0.015	0.002	-0.017	0.018
rs13032049	2	63581507	G	A	0.283	0.01	0.002	-0.041	0.018
rs828867	2	74334462	A	G	0.545	0.009	0.001	-0.006	0.019
rs11692435	2	98275354	A	G	0.085	0.017	0.003	0.023	0.029
rs13024996	2	144225215	A	C	0.364	-0.011	0.002	-0.014	0.017
rs72859280	2	147956293	T	G	0.036	0.023	0.004	-0.021	0.05
rs56337305	2	225475560	C	T	0.383	-0.01	0.001	-0.027	0.017
rs13094887	3	70968431	T	A	0.301	-0.01	0.002	-0.011	0.018
rs62250685	3	85457240	G	A	0.614	-0.014	0.002	0.006	0.017
rs13066454	3	93994255	T	C	0.398	-0.009	0.001	0.009	0.017
rs9838144	3	131576287	C	G	0.209	-0.01	0.002	-0.019	0.021
rs2011092	3	141124607	C	T	0.339	-0.009	0.002	0.079	0.017
rs60654199	3	141267295	A	C	0.063	-0.017	0.003	-0.033	0.037
rs6787172	3	158187811	G	T	0.554	-0.008	0.001	-0.004	0.016
rs3748034	4	3446091	T	G	0.143	-0.012	0.002	-0.039	0.023
rs4501255	4	42151306	G	C	0.235	0.011	0.002	0.005	0.02
rs12499107	4	99678691	G	A	0.131	0.013	0.002	-0.007	0.025
rs1229984	4	100239319	C	T	0.963	0.151	0.004	0.112	0.04
rs10028756	4	100254520	A	G	0.129	-0.019	0.002	0.002	0.028
rs36052336	4	100273594	G	A	0.061	-0.018	0.003	0.059	0.043
rs2165670	4	100286085	A	G	0.106	0.023	0.002	0.000	0.028
rs79139602	4	100444363	T	A	0.021	0.06	0.005	0.053	0.058
rs4699791	4	101243023	A	G	0.096	0.019	0.002	-0.002	0.032
rs13107325	4	103188709	T	C	0.072	-0.028	0.003	-0.009	0.031
rs4690727	4	143648579	G	C	0.718	0.011	0.002	0.01	0.018
rs10004020	4	152968372	A	G	0.72	0.009	0.002	0.015	0.018
rs4916723	5	87854395	C	A	0.416	-0.01	0.001	0.011	0.017
rs12655091	5	144412335	A	G	0.53	-0.008	0.001	-0.015	0.016
rs55872084	5	155902003	T	G	0.235	0.01	0.002	0.021	0.02
rs10085696	7	69783020	G	A	0.186	-0.011	0.002	0.009	0.021
rs6460047	7	73042443	C	T	0.208	0.012	0.002	0.05	0.022
rs10236149	7	98977515	G	A	0.123	-0.013	0.002	0.029	0.025
rs35034355	7	103840115	A	G	0.521	-0.008	0.001	-0.005	0.016
rs6951574	7	153489744	C	T	0.458	0.013	0.001	0.000	0.019
rs13250583	8	20949917	T	C	0.213	-0.01	0.002	0.007	0.02
rs1217091	8	64527399	C	T	0.812	0.012	0.002	0.013	0.021
rs55932213	9	108755622	G	A	0.736	0.009	0.002	0.035	0.02
rs10978550	9	109345993	C	T	0.206	-0.012	0.002	0.021	0.02
rs7074871	10	110507806	A	G	0.255	-0.009	0.002	-0.006	0.019
rs17665139	10	125093880	T	C	0.149	-0.012	0.002	0.007	0.023
rs7950166	11	8642218	T	C	0.637	-0.01	0.002	0.032	0.017

© 2021 Kuav V et al. *JAMA Opthalmology.*

rs11030084	11	27643725	T	C	0.184	-0.011	0.002	-0.036	0.021
rs56030824	11	47397353	A	G	0.322	-0.012	0.002	0.029	0.018
rs10750025	11	113424042	T	C	0.686	0.01	0.002	-0.007	0.017
rs1713676	11	113660576	G	A	0.522	-0.008	0.001	-0.009	0.016
rs4938230	11	116075001	A	C	0.842	0.013	0.002	-0.011	0.022
rs682011	11	121544285	C	T	0.559	0.008	0.001	0.013	0.017
rs12795042	11	133658168	C	A	0.623	-0.008	0.002	-0.032	0.018
rs10876188	12	51895882	T	C	0.457	-0.008	0.001	0.034	0.016
rs3809162	12	54674235	G	A	0.397	0.009	0.001	-0.014	0.017
rs10506274	12	81601464	T	G	0.484	-0.009	0.001	-0.009	0.016
rs4842786	12	92170791	A	G	0.584	-0.009	0.001	0.011	0.018
rs500321	13	27124360	T	A	0.736	-0.01	0.002	-0.035	0.019
rs1123285	14	57274519	G	C	0.335	-0.009	0.002	0.003	0.018
rs2180870	14	58782779	C	T	0.135	-0.012	0.002	-0.016	0.024
rs28929474	14	94844947	T	C	0.018	-0.037	0.005	-0.324	0.068
rs11625650	14	104610138	A	G	0.233	-0.01	0.002	0.058	0.023
rs2472297	15	75027880	T	C	0.249	0.011	0.002	0.061	0.02
rs12907323	15	86796012	G	A	0.411	0.008	0.001	-0.007	0.017
rs2764771	16	20013793	A	G	0.307	0.01	0.002	0.006	0.018
rs17177078	16	24810681	T	C	0.063	-0.022	0.003	0.043	0.034
rs378421	16	28754684	A	G	0.404	-0.011	0.001	-0.056	0.027
rs113443718	16	29892184	A	G	0.305	-0.01	0.002	-0.014	0.018
rs62044525	16	64872590	G	C	0.184	-0.012	0.002	-0.009	0.021
rs7185555	16	69131281	C	G	0.153	-0.011	0.002	0.028	0.024
rs79616692	16	72338507	C	G	0.108	0.016	0.002	0.017	0.027
rs4548913	17	2209888	A	G	0.632	-0.008	0.002	-0.051	0.017
rs3803800	17	7462969	G	A	0.786	0.011	0.002	0.025	0.02
rs2854334	17	29715500	G	A	0.615	0.009	0.001	0.02	0.019
rs10438820	17	78524597	T	C	0.702	0.009	0.002	0.037	0.018
rs9950000	18	53052169	T	C	0.395	-0.009	0.001	0.008	0.017
rs4092465	18	55080437	G	A	0.635	-0.008	0.002	0.028	0.024
rs281379	19	49214274	A	G	0.508	0.014	0.001	0.06	0.016
rs4815364	20	25035711	A	G	0.616	0.009	0.001	-0.01	0.017
rs9607814	22	41946519	A	C	0.2	-0.01	0.002	-0.018	0.021

[a] The variants together explain 1% of the variation in the number of alcoholic drinks per week

© 2021 Kuav V et al. *JAMA Opthalmology.*

eTable 6. Body mass index exposure SNVs and their association with advanced AMD.[a] Chr = chromosome; BP = base position (build 37); EA = effect allele; NEA = non-effect allele; EAF = effect allele frequency; SE = standard error.

SNV	Chr	BP	EA	NEA	EAF	BMI		Advanced AMD	
						**Beta**	**SE**	**Beta**	**SE**
rs1884429	1	11112836	T	C	0.235	0.013	0.002	-0.02	0.02
rs561136	1	23345051	T	C	0.869	-0.019	0.002	-0.029	0.024
rs11577094	1	38026600	T	C	0.079	0.019	0.003	0.011	0.032
rs112566467	1	39562627	T	C	0.207	0.018	0.002	-0.007	0.022
rs12121950	1	49710264	T	G	0.336	0.019	0.002	0.017	0.018
rs12140153	1	62579891	T	G	0.091	-0.035	0.003	-0.012	0.033
rs6690398	1	66447394	A	G	0.55	0.013	0.002	0.008	0.017
rs3101336	1	72751185	T	C	0.383	-0.025	0.002	0.023	0.017
rs12049202	1	77967523	T	C	0.188	0.024	0.002	0.002	0.021
rs2968487	1	96887370	T	C	0.277	0.018	0.002	0.001	0.018
rs12072739	1	98315893	A	G	0.776	-0.017	0.002	0.011	0.02
rs1730859	1	107617707	A	G	0.651	-0.012	0.002	-0.058	0.018
rs17024393	1	110154688	T	C	0.969	-0.064	0.005	-0.076	0.049
rs7534091	1	118864616	A	G	0.718	-0.012	0.002	-0.029	0.019
rs61813324	1	156049877	T	C	0.131	0.029	0.003	-0.053	0.028
rs61828641	1	174321997	A	G	0.109	0.022	0.003	-0.024	0.025
rs543874	1	177889480	A	G	0.779	-0.048	0.002	0.022	0.021
rs10920678	1	190239907	A	G	0.425	0.015	0.002	-0.019	0.017
rs2400414	1	194965200	T	C	0.346	-0.013	0.002	-0.003	0.017
rs2820295	1	201800868	A	G	0.327	0.024	0.002	0.049	0.018
rs6661316	1	210095527	T	C	0.589	0.012	0.002	-0.021	0.017
rs946824	1	243684019	T	C	0.136	0.02	0.003	0.019	0.025
rs13021737	2	632348	A	G	0.157	-0.058	0.002	-0.01	0.021
rs10929925	2	6155557	A	C	0.411	-0.014	0.002	-0.009	0.017
rs7607490	2	12851120	A	G	0.11	0.016	0.003	-0.011	0.028
rs3770799	2	36788616	A	G	0.637	-0.011	0.002	-0.036	0.017
rs7561278	2	48954905	T	C	0.767	0.017	0.002	-0.003	0.021
rs930295	2	50233352	A	C	0.155	0.021	0.002	0.029	0.022
rs4671328	2	58935282	T	G	0.45	0.021	0.002	0.008	0.017
rs6545714	2	59307725	A	G	0.611	-0.019	0.002	-0.038	0.017
rs2861685	2	67837553	T	C	0.586	0.017	0.002	-0.012	0.017
rs12714199	2	86812549	T	C	0.615	-0.014	0.002	-0.002	0.017
rs13002946	2	100801959	A	T	0.265	-0.018	0.002	0.025	0.019
rs13033310	2	133523605	A	G	0.252	0.015	0.002	0.001	0.019
rs1451077	2	147901207	A	G	0.578	-0.017	0.002	0.004	0.017
rs12692596	2	161265910	T	C	0.362	0.012	0.002	0.021	0.017
rs7588437	2	181575281	A	G	0.366	-0.017	0.002	-0.015	0.017
rs7575118	2	182653725	T	C	0.156	0.014	0.002	0.007	0.026
rs7570446	2	193801010	A	C	0.54	0.011	0.002	-0.016	0.016
rs1470545	2	205365851	T	C	0.037	0.037	0.004	0.037	0.041
rs11692326	2	208263279	T	C	0.23	0.015	0.002	0.021	0.02
rs7599312	2	213413231	A	G	0.274	-0.018	0.002	0.012	0.019
rs4973618	2	229002620	A	G	0.662	-0.015	0.002	-0.015	0.017
rs6720868	2	230663576	T	C	0.308	0.015	0.002	-0.045	0.018
rs9808302	2	236854450	A	G	0.528	-0.012	0.002	0.007	0.017
rs59302296	3	9507314	A	T	0.099	0.022	0.003	0.001	0.026
rs10510419	3	12426936	T	G	0.147	-0.017	0.002	-0.044	0.023
rs4857968	3	20714580	A	G	0.293	-0.013	0.002	-0.013	0.018
rs6804842	3	25106437	A	G	0.43	-0.014	0.002	0.001	0.017
rs28350	3	42418446	A	G	0.172	0.017	0.002	0.048	0.022
rs1916801	3	61187046	A	T	0.616	0.017	0.002	-0.006	0.017
rs11915371	3	70539559	A	C	0.802	-0.015	0.002	-0.009	0.021
rs9818122	3	85861064	T	C	0.793	-0.023	0.002	0.007	0.02

© 2021 Kuav V et al. *JAMA Opthalmology.*

rs17681451	3	114399296	A	G	0.076	-0.023	0.003	-0.025	0.032
rs6804181	3	116937546	A	T	0.822	0.014	0.002	0.005	0.021
rs16851483	3	141275436	T	G	0.072	0.035	0.003	-0.029	0.034
rs355777	3	154034950	C	G	0.398	0.015	0.002	0.011	0.017
rs9826775	3	156295341	A	G	0.851	0.016	0.002	-0.003	0.023
rs39654	3	173095123	A	G	0.451	-0.016	0.002	0.023	0.017
rs6443750	3	181329682	T	C	0.199	-0.015	0.002	0.065	0.033
rs9816226	3	185834499	A	T	0.176	-0.032	0.002	-0.008	0.021
rs7616009	3	194881756	A	G	0.16	-0.016	0.002	-0.018	0.023
rs2051559	4	3298800	T	C	0.859	-0.017	0.003	0.031	0.025
rs34811474	4	25408838	A	G	0.222	-0.029	0.002	-0.033	0.02
rs10938397	4	45182527	A	G	0.57	-0.032	0.002	-0.023	0.017
rs2192158	4	55505360	A	G	0.449	0.014	0.002	-0.002	0.017
rs1346841	4	65651730	A	G	0.412	-0.013	0.002	0.003	0.017
rs35851183	4	80717182	A	G	0.642	-0.012	0.002	-0.014	0.017
rs1481012	4	89039082	A	G	0.89	0.019	0.003	0.008	0.027
rs7678054	4	95093855	A	G	0.479	-0.01	0.002	-0.005	0.016
rs13107325	4	103188709	T	C	0.082	0.047	0.003	-0.009	0.031
rs4834272	4	113313986	T	C	0.685	-0.011	0.002	0.01	0.018
rs12509234	4	120319434	T	C	0.721	-0.012	0.002	0.032	0.019
rs2391540	4	130726833	A	T	0.672	-0.014	0.002	0.011	0.017
rs1451109	4	137071335	A	G	0.685	-0.016	0.002	-0.024	0.018
rs57800857	4	140863365	A	C	0.634	0.016	0.002	-0.007	0.018
rs17276464	4	153028860	T	C	0.414	0.01	0.002	0.032	0.017
rs13110266	4	162129844	A	G	0.404	-0.012	0.002	0.002	0.017
rs1522569	4	171632637	T	G	0.818	0.014	0.002	-0.007	0.021
rs1437842	4	173597016	A	G	0.491	-0.011	0.002	-0.03	0.017
rs6850421	4	180187034	A	G	0.456	0.011	0.002	-0.012	0.017
rs698147	5	3513485	A	G	0.447	0.012	0.002	0.019	0.016
rs6890310	5	27193573	A	G	0.292	-0.012	0.002	0.002	0.019
rs6451675	5	43110855	C	G	0.324	-0.014	0.002	-0.023	0.018
rs4700608	5	63026280	T	C	0.524	-0.016	0.002	0.006	0.016
rs2112347	5	75015242	T	G	0.63	0.028	0.002	0.041	0.017
rs10942267	5	80841914	A	G	0.693	0.015	0.002	-0.019	0.018
rs1501673	5	87963600	A	G	0.136	0.029	0.003	0.027	0.024
rs7713317	5	95716722	A	G	0.72	-0.017	0.002	0.004	0.018
rs11739877	5	105876806	T	C	0.623	0.012	0.002	-0.01	0.017
rs288230	5	107422067	T	C	0.839	0.024	0.002	-0.002	0.022
rs459552	5	112176756	A	T	0.775	-0.013	0.002	0.004	0.021
rs6864049	5	124330522	A	G	0.489	-0.012	0.002	-0.031	0.017
rs13174863	5	139080745	A	G	0.854	-0.02	0.002	0.019	0.025
rs7734385	5	158460212	A	G	0.445	-0.01	0.002	-0.013	0.017
rs2861089	5	164557954	A	T	0.381	0.011	0.002	0.006	0.017
rs2053682	5	170599327	A	C	0.678	0.017	0.002	0.035	0.018
rs6556301	5	176527577	T	G	0.371	-0.011	0.002	0.042	0.017
rs2228213	6	12124855	A	G	0.337	-0.014	0.002	0.002	0.017
rs11757278	6	13180454	T	C	0.696	0.013	0.002	0.002	0.018
rs3806114	6	20482335	A	G	0.692	-0.012	0.002	-0.006	0.019
rs6932930	6	34677103	A	G	0.785	-0.026	0.002	0.012	0.02
rs2206277	6	50798526	T	C	0.156	0.041	0.002	-0.084	0.021
rs816367	6	53995542	C	G	0.649	-0.011	0.002	0.003	0.017
rs2622274	6	64240516	T	G	0.455	-0.011	0.002	0.027	0.017
rs7769594	6	83447813	T	C	0.16	0.016	0.002	0.025	0.023
rs12206094	6	108906200	T	C	0.29	-0.014	0.002	-0.023	0.018
rs2357760	6	120213880	A	G	0.674	0.014	0.002	-0.003	0.017
rs1269175	6	126040435	A	G	0.511	0.011	0.002	-0.01	0.017
rs2246012	6	131898208	T	C	0.851	-0.016	0.002	-0.053	0.023
rs6922607	6	142703483	A	G	0.808	-0.013	0.002	-0.003	0.021

© 2021 Kuav V et al. *JAMA Opthalmology.*

rs765875	6	143185683	T	C	0.469	-0.013	0.002	-0.007	0.016
rs487152	6	160774486	A	C	0.497	0.011	0.002	0.007	0.016
rs13191362	6	163033350	A	G	0.857	0.024	0.003	0.046	0.026
rs4721089	7	1872921	T	C	0.783	0.017	0.002	0.015	0.02
rs6463489	7	5542513	T	C	0.096	0.017	0.003	0.007	0.029
rs4307239	7	24354300	A	G	0.536	-0.012	0.002	-0.02	0.017
rs215614	7	32347335	A	G	0.625	-0.014	0.002	-0.016	0.017
rs10499694	7	50614173	A	G	0.494	0.013	0.002	0.019	0.016
rs740157	7	77055885	A	G	0.45	0.012	0.002	0.023	0.017
rs1852006	7	77829768	A	G	0.376	-0.015	0.002	-0.039	0.017
rs1030015	7	78139581	T	G	0.542	0.011	0.002	-0.004	0.016
rs1048303	7	100804140	T	C	0.582	-0.011	0.002	-0.014	0.017
rs1899689	7	121964349	T	C	0.394	0.012	0.002	0.021	0.017
rs7802342	7	137435925	T	G	0.705	-0.012	0.002	-0.01	0.021
rs11525873	7	138817193	T	C	0.9	0.023	0.003	-0.062	0.028
rs2907948	7	150638484	A	G	0.244	-0.015	0.002	0.03	0.019
rs1658820	8	4288577	T	G	0.239	0.013	0.002	0.02	0.02
rs4841504	8	11024663	A	C	0.501	-0.017	0.002	-0.001	0.017
rs4123853	8	14091025	T	C	0.373	0.014	0.002	-0.007	0.017
rs354508	8	15535226	T	C	0.162	-0.015	0.002	-0.001	0.022
rs1982441	8	28021769	T	G	0.132	0.017	0.003	-0.027	0.024
rs10954772	8	30863938	T	C	0.319	0.016	0.002	0.008	0.018
rs7826312	8	32400115	T	C	0.432	-0.011	0.002	-0.003	0.017
rs36061954	8	38329650	T	C	0.398	0.012	0.002	-0.005	0.017
rs12681792	8	62054463	A	C	0.202	0.015	0.002	0.002	0.022
rs1431659	8	73439070	A	G	0.274	0.019	0.002	-0.003	0.019
rs17405819	8	76806584	T	C	0.685	0.021	0.002	-0.014	0.018
rs2196618	8	85089437	A	G	0.263	-0.014	0.002	-0.02	0.019
rs12680842	8	95582606	A	G	0.684	0.014	0.002	0.013	0.018
rs1383592	8	106430676	A	G	0.212	0.012	0.002	0.003	0.02
rs3808477	8	116670347	T	C	0.274	-0.018	0.002	0.01	0.019
rs72673947	8	118884379	A	G	0.89	-0.022	0.003	0.043	0.027
rs1106761	8	142619234	A	G	0.375	0.013	0.002	-0.004	0.017
rs10099330	8	143383694	A	G	0.538	-0.012	0.002	-0.006	0.017
rs7037266	9	6942940	A	C	0.358	-0.011	0.002	-0.013	0.017
rs4740619	9	15634326	T	C	0.544	0.019	0.002	-0.027	0.017
rs2183824	9	28412078	T	C	0.314	0.023	0.002	0.002	0.018
rs6476617	9	37200103	A	G	0.38	-0.015	0.002	0.017	0.017
rs2134858	9	73837155	T	C	0.512	-0.012	0.002	0.01	0.016
rs10116186	9	80532374	A	G	0.459	-0.012	0.002	0.006	0.017
rs2777768	9	84186734	A	G	0.723	0.012	0.002	0.02	0.019
rs7357754	9	92207308	A	G	0.499	-0.012	0.002	0.03	0.017
rs3811125	9	94187247	T	C	0.281	-0.015	0.002	0.027	0.02
rs10761247	9	96403367	A	G	0.587	-0.011	0.002	-0.016	0.016
rs6477694	9	111932342	T	C	0.642	-0.013	0.002	-0.021	0.017
rs1928295	9	120378483	T	C	0.571	0.013	0.002	-0.033	0.016
rs10984756	9	122651784	C	G	0.905	-0.018	0.003	-0.006	0.028
rs3739514	9	133783025	A	G	0.338	0.013	0.002	0.008	0.018
rs10858334	9	137989785	C	G	0.848	-0.015	0.003	-0.044	0.027
rs7893571	10	16750129	T	G	0.674	0.013	0.002	0.001	0.018
rs1277733	10	18562538	T	C	0.766	0.012	0.002	-0.003	0.02
rs7084454	10	21821274	A	G	0.309	0.02	0.002	0.012	0.018
rs4097319	10	33860515	T	G	0.564	0.011	0.002	0.007	0.017
rs12765914	10	34013507	T	C	0.08	0.023	0.003	0.035	0.03
rs10823893	10	53677313	A	G	0.407	0.012	0.002	0.03	0.017
rs7070670	10	61842645	T	C	0.323	-0.013	0.002	0.009	0.019
rs12098284	10	76047464	T	C	0.121	0.018	0.003	0.033	0.025
rs7899106	10	87410904	A	G	0.95	-0.033	0.004	0.033	0.039

© 2021 Kuav V et al. *JAMA Opthalmology.*

rs10887584	10	88112323	A	G	0.448	0.013	0.002	-0.007	0.017
rs2439823	10	99778226	A	G	0.451	-0.017	0.002	0.022	0.017
rs34277166	10	102634853	A	G	0.44	0.014	0.002	-0.053	0.019
rs4290163	10	104610926	T	G	0.398	0.013	0.002	0.039	0.017
rs7903146	10	114758349	T	C	0.279	-0.018	0.002	-0.041	0.018
rs845084	10	125220036	A	G	0.267	0.014	0.002	0.025	0.019
rs17636031	10	126594078	T	C	0.724	-0.015	0.002	0.022	0.024
rs4880341	10	133992689	T	C	0.573	-0.013	0.002	-0.022	0.017
rs4256980	11	8673939	C	G	0.331	-0.019	0.002	-0.029	0.017
rs1982350	11	13350131	A	G	0.356	-0.016	0.002	-0.002	0.017
rs5215	11	17408630	T	C	0.631	0.011	0.002	-0.028	0.017
rs6265	11	27679916	T	C	0.184	-0.041	0.002	-0.038	0.021
rs2065418	11	30422068	T	G	0.646	0.014	0.002	-0.015	0.017
rs2862996	11	43653833	T	G	0.701	-0.022	0.002	-0.024	0.018
rs7124681	11	47529947	A	C	0.416	0.026	0.002	0.01	0.017
rs6591407	11	56914157	A	C	0.198	-0.012	0.002	-0.016	0.021
rs592483	11	69445173	T	C	0.593	-0.014	0.002	0.002	0.017
rs12282785	11	76476030	A	C	0.22	-0.016	0.002	-0.038	0.02
rs349088	11	84814393	A	C	0.478	-0.013	0.002	-0.003	0.016
rs2605603	11	93221105	A	G	0.481	-0.01	0.002	0.017	0.017
rs12286929	11	115022404	A	G	0.501	-0.018	0.002	0.01	0.016
rs76942203	11	116973247	A	G	0.059	0.026	0.004	0.035	0.035
rs3825061	11	118944675	T	C	0.386	0.014	0.002	-0.031	0.017
rs11218510	11	121922587	A	G	0.395	-0.014	0.002	0.012	0.017
rs7944782	11	130795698	T	G	0.502	-0.014	0.002	0.015	0.017
rs12364470	11	134601012	T	G	0.854	-0.019	0.002	-0.009	0.026
rs11611246	12	939480	T	G	0.199	0.022	0.002	-0.022	0.021
rs12422552	12	14413931	C	G	0.269	-0.013	0.002	0.042	0.019
rs10744146	12	17212881	A	G	0.536	-0.011	0.002	-0.023	0.016
rs11047132	12	24008435	T	G	0.918	-0.023	0.003	0.029	0.034
rs11170468	12	39430048	A	C	0.783	0.013	0.002	0.006	0.019
rs7138803	12	50247468	A	G	0.387	0.03	0.002	-0.011	0.017
rs7975187	12	60964108	A	G	0.771	-0.014	0.002	-0.012	0.02
rs650198	12	69674595	T	C	0.729	-0.014	0.002	-0.021	0.018
rs11115176	12	82465797	T	C	0.784	0.013	0.002	-0.041	0.02
rs704061	12	89771903	T	C	0.554	-0.014	0.002	0.017	0.016
rs11105839	12	91237920	A	T	0.373	-0.011	0.002	-0.038	0.017
rs6539064	12	103706754	C	G	0.747	0.019	0.002	0.007	0.019
rs12321904	12	109989228	T	G	0.5	0.01	0.002	0.006	0.016
rs12369179	12	122963550	T	C	0.085	-0.034	0.003	-0.025	0.031
rs11836108	12	133391022	A	G	0.296	0.011	0.002	-0.018	0.018
rs1045411	13	31033232	T	C	0.281	-0.014	0.002	0.034	0.021
rs9595908	13	33184288	T	C	0.643	0.015	0.002	0.001	0.017
rs9603697	13	40783323	T	C	0.318	0.013	0.002	0.009	0.018
rs41284828	13	50963685	A	G	0.03	-0.034	0.006	0.016	0.049
rs12429545	13	54102206	A	G	0.122	0.031	0.002	-0.012	0.025
rs8181823	13	65477940	A	C	0.234	-0.013	0.002	0.001	0.019
rs9540493	13	66205704	A	G	0.458	0.013	0.002	0.019	0.017
rs4421883	13	79561962	T	C	0.495	-0.01	0.002	0.008	0.016
rs77432547	13	86494817	A	G	0.723	-0.017	0.002	-0.004	0.019
rs1927790	13	96922191	T	C	0.606	-0.014	0.002	-0.012	0.017
rs1536053	13	111982291	T	C	0.31	-0.012	0.002	0.01	0.02
rs10132280	14	25928179	A	C	0.311	-0.021	0.002	-0.037	0.019
rs872281	14	40834177	T	C	0.176	-0.015	0.002	0.005	0.023
rs1491905	14	47296286	T	C	0.473	0.015	0.002	0.007	0.017
rs217669	14	62360075	T	C	0.722	-0.017	0.002	-0.002	0.019
rs1954494	14	62593297	T	C	0.561	0.01	0.002	0.003	0.016
rs7144011	14	79940383	T	G	0.233	0.026	0.002	-0.025	0.02

© 2021 Kuav V et al. *JAMA Opthalmology.*

rs1951455	14	91512339	T	C	0.281	-0.015	0.002	-0.012	0.018
rs942066	14	94031914	A	G	0.373	-0.02	0.002	-0.03	0.017
rs4906263	14	103249127	C	G	0.656	-0.018	0.002	-0.002	0.018
rs709400	14	104149475	A	G	0.621	0.015	0.002	-0.004	0.017
rs7181610	15	35826859	A	T	0.855	0.015	0.003	0.002	0.024
rs11636611	15	36391965	T	C	0.498	0.01	0.002	0.019	0.016
rs6493498	15	51754451	T	C	0.447	0.014	0.002	-0.036	0.017
rs8024806	15	53473990	T	C	0.937	0.025	0.004	0.028	0.034
rs12438629	15	62122539	C	G	0.968	0.035	0.005	0.021	0.054
rs1996120	15	68181534	A	G	0.423	-0.017	0.002	-0.009	0.017
rs11856579	15	78012688	A	G	0.261	-0.016	0.002	0.009	0.019
rs11633626	15	95271378	A	C	0.631	-0.016	0.002	-0.002	0.017
rs2715423	15	99511873	A	G	0.281	-0.012	0.002	0.005	0.018
rs11866815	16	387867	T	C	0.257	-0.015	0.002	-0.021	0.019
rs2601777	16	4035068	A	G	0.386	-0.014	0.002	0.012	0.017
rs194809	16	23804956	A	G	0.187	0.013	0.002	0.008	0.021
rs2342892	16	24540806	T	G	0.486	0.013	0.002	-0.042	0.017
rs7498665	16	28883241	A	G	0.618	-0.029	0.002	0.026	0.017
rs4609871	16	29932064	T	C	0.54	0.022	0.002	-0.022	0.017
rs9937053	16	53799507	A	G	0.437	0.072	0.002	-0.029	0.016
rs11075489	16	62803841	T	C	0.474	-0.012	0.002	-0.01	0.017
rs889398	16	69556715	T	C	0.415	-0.02	0.002	-0.002	0.017
rs12449219	16	77261943	C	G	0.856	-0.016	0.003	0.033	0.024
rs12922346	16	82438337	C	G	0.264	0.013	0.002	-0.016	0.02
rs7206608	16	82872628	C	G	0.675	-0.013	0.002	0.025	0.018
rs3923783	17	1843189	A	C	0.178	-0.022	0.002	0.046	0.022
rs1320251	17	21264396	T	C	0.457	-0.018	0.002	-0.014	0.017
rs3930349	17	31475545	A	C	0.209	-0.014	0.002	-0.018	0.02
rs1106908	17	34942595	A	G	0.445	-0.016	0.002	-0.009	0.017
rs6607337	17	35057373	T	C	0.298	-0.012	0.002	-0.03	0.018
rs11655587	17	47140794	T	C	0.357	-0.021	0.002	-0.017	0.018
rs8075273	17	61728881	A	C	0.288	-0.014	0.002	-0.011	0.018
rs2619976	17	71754545	T	C	0.401	0.011	0.002	-0.026	0.017
rs3744017	17	73871467	A	G	0.193	0.014	0.002	0.037	0.021
rs12939549	17	78611724	A	G	0.557	0.018	0.002	-0.018	0.017
rs7238896	18	1840658	A	G	0.858	-0.022	0.003	0.002	0.024
rs891387	18	21103909	T	C	0.495	0.021	0.002	-0.034	0.017
rs1356506	18	40708038	T	C	0.629	0.014	0.002	0.02	0.017
rs7239114	18	45921214	A	G	0.541	0.012	0.002	-0.016	0.021
rs6567160	18	57829135	T	C	0.752	-0.055	0.002	0.03	0.02
rs594821	18	76745589	T	C	0.091	-0.019	0.003	-0.027	0.031
rs56356382	19	4064057	T	C	0.805	0.022	0.002	0.008	0.022
rs273505	19	18217147	T	C	0.578	-0.017	0.002	-0.024	0.017
rs12462975	19	30272202	A	G	0.324	0.019	0.002	-0.024	0.018
rs10518269	19	31028666	T	C	0.165	-0.017	0.002	-0.009	0.022
rs11672660	19	46180184	T	C	0.187	-0.034	0.002	-0.004	0.02
rs12151152	19	47563532	A	G	0.409	-0.021	0.002	-0.01	0.019
rs1884389	20	1410582	T	C	0.439	-0.011	0.002	0.025	0.017
rs615568	20	3008775	T	G	0.52	-0.01	0.002	-0.012	0.017
rs1884897	20	6612832	A	G	0.375	-0.018	0.002	0.015	0.017
rs1409818	20	21381121	T	C	0.107	0.02	0.003	-0.033	0.027
rs8122855	20	25192049	A	G	0.338	0.014	0.002	0.014	0.017
rs16989232	20	39291784	A	G	0.386	0.012	0.002	0.01	0.017
rs17201143	20	50402079	T	C	0.305	-0.011	0.002	-0.011	0.018
rs17806224	20	51065854	A	G	0.181	-0.026	0.002	0.022	0.022
rs6512302	20	62691550	C	G	0.739	0.013	0.002	-0.006	0.02
rs2832283	21	30690558	A	G	0.225	0.012	0.002	-0.02	0.022
rs8134638	21	40644170	T	C	0.627	-0.013	0.002	0.002	0.018

© 2021 Kuav V et al. *JAMA Opthalmology.*

rs427943	21	46570896	A	C	0.429	-0.018	0.002	0.007	0.017
rs12628891	22	38317137	T	C	0.319	-0.012	0.002	-0.039	0.018
rs12628051	22	40654276	T	C	0.641	0.016	0.002	-0.02	0.017

[a] The variants together explain 4% of the variation in BMI

© 2021 Kuav V et al. *JAMA Opthalmology.*

eTable 7. Systolic blood pressure exposure SNVs and their association with advanced AMD.[a]
Chr = chromosome; BP = base position (build 37); EA = effect allele; NEA = non-effect allele; EAF = effect allele frequency; SE = standard error.

SNV	Chr	BP	EA	NEA	EAF	Systolic Blood Pressure		Advanced AMD	
						**Beta**	**SE**	**Beta**	**SE**
rs7546498	1	1740255	G	T	0.505	0.257	0.039	0.006	0.017
rs783621	1	42368035	A	G	0.446	0.289	0.038	0.005	0.017
rs2404715	1	57008778	C	T	0.909	0.4	0.066	-0.021	0.03
rs60199046	1	59663341	A	G	0.729	0.24	0.043	0.005	0.018
rs786919	1	89281529	A	G	0.395	0.262	0.039	0.04	0.017
rs13420463	2	37517566	A	G	0.77	0.282	0.044	0.022	0.02
rs13403122	2	43078758	C	T	0.721	0.25	0.043	0.002	0.019
rs3731818	2	86368804	G	A	0.676	0.24	0.041	0.006	0.018
rs13024657	2	175472839	C	T	0.835	-0.296	0.054	0.022	0.023
rs6434404	2	191494411	A	G	0.308	0.311	0.043	-0.018	0.019
rs1250247	2	216299629	C	G	0.263	0.31	0.044	-0.022	0.019
rs1063281	2	218668732	C	T	0.392	0.247	0.039	-0.01	0.017
rs7590201	2	227192443	G	T	0.45	-0.216	0.039	-0.025	0.017
rs12630213	3	14954411	C	T	0.348	0.276	0.04	0.002	0.018
rs12636552	3	70972466	A	G	0.676	0.241	0.041	0.007	0.017
rs6803322	3	84986088	C	A	0.675	0.228	0.042	0.002	0.018
rs4141663	3	124551967	C	T	0.57	0.258	0.038	0.018	0.017
rs9844972	3	150097635	G	C	0.921	-0.441	0.077	-0.003	0.034
rs113161639	3	154615819	G	T	0.902	0.451	0.065	0.081	0.03
rs2178452	3	160370160	G	A	0.671	0.259	0.04	0.021	0.018
rs13104866	4	38402183	G	A	0.481	0.278	0.038	0.002	0.017
rs12504699	4	48934298	G	A	0.637	0.221	0.039	0.005	0.017
rs55940751	4	77365891	C	T	0.567	0.216	0.039	-0.049	0.017
rs13112725	4	106911742	G	C	0.242	-0.297	0.044	0.022	0.02
rs7665304	4	109025379	A	C	0.413	0.22	0.039	0.022	0.017
rs66887589	4	120509279	T	C	0.507	-0.214	0.038	-0.025	0.017
rs893929	4	144187380	G	A	0.547	0.233	0.039	-0.035	0.017
rs303343	5	15312553	C	T	0.571	-0.229	0.039	-0.005	0.017
rs1694068	5	53283630	T	A	0.392	-0.245	0.04	0.021	0.017
rs17286052	5	87430302	A	G	0.845	0.36	0.055	0.058	0.024
rs4475250	5	114375552	G	A	0.556	0.243	0.038	0.009	0.017
rs6595838	5	127868199	G	A	0.71	-0.267	0.042	0.000	0.018
rs2050663	6	79753394	T	C	0.484	-0.229	0.039	0.049	0.017
rs35410524	6	96885405	C	T	0.804	-0.305	0.049	-0.017	0.021
rs12670854	7	1731866	A	G	0.888	0.36	0.063	0.017	0.028
rs17423264	7	108090255	C	T	0.907	0.366	0.067	-0.006	0.034
rs6957161	7	131361319	A	G	0.253	0.256	0.043	-0.03	0.019
rs80073370	8	19833156	A	T	0.917	0.404	0.07	0.012	0.031
rs7008914	8	25880400	T	C	0.748	0.238	0.044	0.013	0.02
rs11993898	8	51936632	T	C	0.822	-0.326	0.049	0.015	0.022
rs112875651	8	126506694	G	A	0.621	0.226	0.04	-0.036	0.017
rs76735299	8	142396481	G	A	0.915	-0.429	0.078	-0.015	0.033
rs62524579	8	144060955	G	A	0.476	0.244	0.039	0.006	0.017
rs7041664	9	8010674	C	A	0.717	-0.247	0.043	0.014	0.019
rs28663144	9	113198891	A	C	0.962	-0.694	0.105	-0.004	0.043
rs10818775	9	125755571	C	T	0.886	0.332	0.056	0.017	0.026
rs1848797	10	64552934	A	G	0.611	0.265	0.039	-0.009	0.017
rs4110517	10	96650328	A	G	0.216	-0.282	0.046	0.022	0.02
rs4551692	10	102556453	G	A	0.111	-0.419	0.062	-0.053	0.032
rs34872471	10	114754071	T	C	0.702	-0.25	0.042	0.041	0.018
rs360158	11	9753601	G	A	0.41	-0.301	0.039	-0.013	0.017

© 2021 Kuav V et al. *JAMA Opthalmology.*

rs2585810	11	28483787	G	A	0.657	-0.22	0.04	0.013	0.017
rs7107356	11	47676170	A	G	0.49	-0.309	0.038	0.01	0.016
rs61448762	11	48923756	G	A	0.879	0.358	0.061	0.01	0.03
rs685149	11	57657413	A	G	0.343	-0.311	0.042	0.022	0.018
rs1938598	11	58413910	T	C	0.755	0.33	0.044	-0.008	0.019
rs7927515	11	76125330	C	A	0.651	-0.235	0.04	0.006	0.017
rs2289125	11	89224453	A	C	0.22	-0.274	0.045	0.055	0.021
rs7951348	11	107081841	C	T	0.522	-0.252	0.038	-0.01	0.017
rs11168244	12	48202941	C	T	0.798	0.318	0.05	-0.02	0.022
rs7977389	12	49981722	T	C	0.892	0.381	0.061	-0.003	0.026
rs10747570	12	50509937	A	G	0.393	0.256	0.04	-0.024	0.017
rs17210898	12	51056511	G	A	0.96	0.556	0.101	-0.066	0.043
rs10784502	12	66343810	C	T	0.479	-0.235	0.038	-0.014	0.016
rs9565436	13	36213631	A	C	0.871	-0.303	0.054	-0.014	0.024
rs3011549	13	113634937	A	C	0.293	0.326	0.048	-0.005	0.018
rs9314907	13	115015163	C	T	0.767	-0.292	0.045	0.016	0.02
rs8904	14	35871217	G	A	0.625	-0.259	0.039	0.000	0.017
rs7161323	14	53366149	C	T	0.394	-0.275	0.042	0.038	0.018
rs937213	15	40322124	T	C	0.589	0.256	0.04	0.028	0.018
rs2759308	15	81016227	G	A	0.523	-0.276	0.039	-0.017	0.017
rs12596053	16	4946794	A	C	0.439	-0.275	0.039	-0.021	0.017
rs9935770	16	21091291	C	T	0.57	0.223	0.038	-0.006	0.017
rs200541	16	24733141	A	G	0.193	-0.281	0.049	0.007	0.021
rs56249585	16	65265702	C	T	0.47	-0.373	0.064	-0.005	0.017
rs35261357	16	75444572	C	T	0.438	-0.265	0.039	-0.054	0.017
rs4295	17	61556298	C	G	0.393	0.24	0.042	0.015	0.017
rs7225219	17	62407559	T	A	0.349	-0.248	0.042	0.023	0.017
rs4788913	17	73950216	G	A	0.355	-0.283	0.04	0.017	0.017
rs8073626	17	76790279	C	T	0.47	0.217	0.038	-0.005	0.017
rs12606620	18	42008097	G	T	0.696	0.292	0.042	0.02	0.019
rs2193635	18	43096236	C	T	0.818	-0.274	0.048	0.006	0.021
rs167479	19	11526765	G	T	0.545	0.41	0.043	0.016	0.017
rs8105753	19	31927547	A	C	0.615	0.249	0.04	0.06	0.019
rs6129880	20	40251829	T	G	0.805	0.264	0.047	0.012	0.021
rs6031435	20	42797358	A	G	0.541	-0.214	0.038	0.001	0.017
rs6090040	20	62692060	A	C	0.44	0.265	0.044	0.01	0.017
rs13050325	21	16343812	A	G	0.755	-0.258	0.044	0.033	0.02
rs34887403	22	29151150	G	A	0.851	-0.304	0.055	-0.013	0.024

[a] The variants together explain 1.1% of the variation in systolic blood pressure

© 2021 Kuav V et al. *JAMA Opthalmology.*

eTable 8. Diastolic blood pressure exposure SNVs and their association with advanced AMD.[a]
Chr = chromosome; BP = base position (build 37); EA = effect allele; NEA = non-effect allele; EAF = effect allele frequency; SE = standard error.

SNV	Chr	BP	EA	NEA	EAF	Diastolic Blood Pressure		Advanced AMD	
						**Beta**	**SE**	**Beta**	**SE**
rs7546498	1	1740255	G	T	0.505	0.147	0.024	0.006	0.017
rs11102916	1	115836746	C	A	0.982	-0.575	0.084	0.074	0.063
rs12405515	1	172357441	G	T	0.422	0.17	0.024	-0.023	0.017
rs17046596	1	217722449	A	C	0.735	-0.151	0.027	-0.004	0.019
rs4653889	1	228112121	A	G	0.477	0.152	0.024	0.01	0.017
rs6428947	1	236326005	C	G	0.836	0.205	0.031	0.025	0.023
rs6429422	1	243472801	T	G	0.675	-0.249	0.025	0.044	0.018
rs10198275	2	25130542	A	C	0.568	0.135	0.023	0.007	0.017
rs13403122	2	43078758	C	T	0.721	0.195	0.027	0.002	0.019
rs1876487	2	73114352	A	C	0.273	-0.162	0.027	-0.034	0.02
rs3923097	2	124020790	T	A	0.936	0.273	0.048	0.04	0.034
rs58117425	2	145681570	G	A	0.774	-0.192	0.028	0.02	0.02
rs4972805	2	177012570	C	T	0.68	0.143	0.025	-0.008	0.018
rs1063281	2	218668732	C	T	0.392	0.17	0.024	-0.01	0.017
rs7590201	2	227192443	G	T	0.45	-0.138	0.024	-0.025	0.017
rs12630213	3	14954411	C	T	0.348	0.153	0.025	0.002	0.018
rs76398786	3	48731450	C	T	0.961	-0.382	0.067	0.02	0.052
rs3749237	3	49770032	G	A	0.689	-0.165	0.025	0.033	0.018
rs2236973	3	50474284	T	C	0.833	-0.182	0.033	0.046	0.024
rs9845655	3	56701328	T	C	0.734	0.161	0.026	-0.053	0.019
rs1053711	3	57743246	G	A	0.691	0.153	0.026	0.008	0.018
rs6795735	3	64705365	C	T	0.563	0.159	0.023	-0.125	0.017
rs9882772	3	122110149	T	C	0.58	-0.133	0.023	-0.027	0.017
rs9864898	3	138111751	C	T	0.843	-0.19	0.032	0.024	0.023
rs113161639	3	154615819	G	T	0.902	0.288	0.04	0.081	0.03
rs4686683	3	185307363	T	G	0.59	-0.172	0.024	-0.017	0.017
rs13112725	4	106911742	G	C	0.242	-0.15	0.027	0.022	0.02
rs66887589	4	120509279	T	C	0.507	-0.206	0.023	-0.025	0.017
rs168643	5	50935900	T	C	0.355	0.139	0.025	-0.044	0.017
rs111304266	5	56589542	C	G	0.968	-0.37	0.067	0.067	0.054
rs258494	5	75038718	C	G	0.379	0.216	0.024	-0.015	0.018
rs17286052	5	87430302	A	G	0.845	0.188	0.034	0.058	0.024
rs17082391	5	91900785	C	G	0.971	0.375	0.066	-0.063	0.052
rs4475250	5	114375552	G	A	0.556	0.136	0.023	0.009	0.017
rs7734334	5	131815004	C	A	0.671	-0.154	0.025	-0.017	0.018
rs72812846	5	173377636	T	A	0.735	0.202	0.027	0.007	0.02
rs169287	6	27854760	C	A	0.841	0.215	0.031	-0.049	0.023
rs210156	6	33517362	A	G	0.352	-0.148	0.025	0.012	0.017
rs1544935	6	39124448	T	G	0.783	0.176	0.029	0.018	0.02
rs13205180	6	51832494	C	T	0.524	-0.143	0.024	0.018	0.017
rs2050663	6	79753394	T	C	0.484	-0.174	0.024	0.049	0.017
rs36061333	6	116311763	C	G	0.772	0.16	0.029	0.041	0.02
rs1761870	6	117264985	G	A	0.176	0.175	0.03	0.027	0.022
rs1630266	6	118612943	G	A	0.914	-0.252	0.042	-0.021	0.029
rs4709746	6	164133001	C	T	0.864	0.19	0.035	0.038	0.025
rs903432	6	166175471	A	G	0.931	0.313	0.045	-0.017	0.033
rs11486794	7	2491918	C	T	0.851	0.219	0.036	0.003	0.024
rs34594435	7	72977249	C	T	0.818	0.183	0.03	-0.022	0.021
rs76627715	7	80387316	T	C	0.879	0.216	0.035	-0.038	0.027
rs1015538	7	99626035	A	G	0.325	0.137	0.025	-0.051	0.018
rs11556924	7	129663496	C	T	0.639	0.181	0.025	0.024	0.017

© 2021 Kuav V et al. *JAMA Opthalmology.*

rs6957161	7	131361319	A	G	0.253	0.157	0.026	-0.03	0.019
rs111630016	7	158048396	C	T	0.952	0.324	0.059	0.041	0.042
rs2280861	8	23404785	A	G	0.754	-0.173	0.027	-0.014	0.019
rs10103353	8	82849452	C	T	0.592	0.147	0.024	0.005	0.017
rs62524579	8	144060955	G	A	0.476	0.147	0.024	0.006	0.017
rs507666	9	136149399	G	A	0.805	0.169	0.03	-0.038	0.02
rs10751962	10	4172711	C	T	0.098	-0.219	0.04	0.051	0.031
rs2246438	10	45273079	G	A	0.723	0.16	0.026	0.024	0.018
rs1848797	10	64552934	A	G	0.611	0.191	0.024	-0.009	0.017
rs4551692	10	102556453	G	A	0.111	-0.255	0.038	-0.053	0.032
rs17617337	10	121426884	C	T	0.788	0.172	0.029	0.015	0.02
rs360158	11	9753601	G	A	0.41	-0.165	0.024	-0.013	0.017
rs7928655	11	13300252	C	G	0.3	-0.143	0.025	0.005	0.018
rs11030119	11	27728102	G	A	0.716	0.18	0.026	-0.015	0.018
rs61879810	11	31821467	A	G	0.153	0.198	0.033	0.019	0.024
rs7107356	11	47676170	A	G	0.49	-0.149	0.023	0.01	0.016
rs72930293	11	69073420	C	T	0.899	0.234	0.039	0.045	0.027
rs11021221	11	95308854	T	A	0.845	0.213	0.032	-0.024	0.023
rs7116797	11	116707338	A	G	0.115	0.214	0.035	0.057	0.025
rs11168244	12	48202941	C	T	0.798	0.165	0.03	-0.02	0.022
rs10747570	12	50509937	A	G	0.393	0.184	0.024	-0.024	0.017
rs17210898	12	51056511	G	A	0.96	0.351	0.061	-0.066	0.043
rs1152958	12	70325669	G	A	0.688	0.138	0.025	0.039	0.018
rs7980687	12	123822711	G	A	0.793	0.196	0.029	-0.063	0.02
rs7989823	13	110959643	A	C	0.421	-0.15	0.026	0.009	0.02
rs3934939	13	114503990	A	G	0.766	0.163	0.028	0.004	0.026
rs36226649	14	24835500	T	C	0.942	-0.297	0.05	0.000	0.036
rs7161323	14	53366149	C	T	0.394	-0.147	0.026	0.038	0.018
rs4923910	15	42086340	G	C	0.633	-0.178	0.024	-0.017	0.017
rs35654783	15	44018656	T	C	0.283	0.166	0.026	-0.022	0.018
rs11631778	15	71606380	G	A	0.337	-0.141	0.025	-0.05	0.018
rs2759308	15	81016227	G	A	0.523	-0.136	0.024	-0.017	0.017
rs2034618	15	83799632	C	T	0.773	0.188	0.028	0.011	0.02
rs12906962	15	95312071	T	C	0.671	-0.16	0.025	0.008	0.018
rs4984497	15	96635899	T	C	0.372	0.148	0.025	-0.016	0.018
rs12596053	16	4946794	A	C	0.439	-0.162	0.024	-0.021	0.017
rs72799341	16	30936743	G	A	0.76	-0.161	0.027	0.045	0.019
rs12928482	16	81513871	G	A	0.718	0.147	0.026	0.018	0.019
rs460105	16	89682006	T	C	0.508	0.178	0.026	0.005	0.017
rs67833703	17	3888437	C	T	0.687	-0.147	0.026	0.04	0.018
rs78378222	17	7571752	T	G	0.986	-0.638	0.111	-0.159	0.08
rs35565381	17	16175025	T	C	0.544	-0.14	0.024	-0.015	0.017
rs4295	17	61556298	C	G	0.393	0.14	0.026	0.015	0.017
rs167479	19	11526765	G	T	0.545	0.251	0.026	0.016	0.017
rs4808569	19	17218970	C	A	0.8	0.162	0.03	-0.017	0.021
rs34331990	19	30321561	T	G	0.586	-0.154	0.024	0.024	0.018
rs8105753	19	31927547	A	C	0.615	0.137	0.024	0.06	0.019
rs6060114	20	30169673	T	C	0.857	0.237	0.033	-0.048	0.022
rs6129880	20	40251829	T	G	0.805	0.174	0.029	0.012	0.021
rs6019378	20	47309716	C	T	0.558	0.156	0.023	-0.006	0.016
rs112204826	21	44721027	C	T	0.965	-0.395	0.071	0.112	0.062
rs8139817	22	18468369	A	C	0.71	-0.143	0.026	-0.011	0.019

[a] The variants together explain 1.3% of the variation in diastolic blood pressure

© 2021 Kuav V et al. *JAMA Opthalmology.*

eTable 9. Type 2 diabetes exposure SNVs and their association with advanced AMD. Chr = chromosome; BP = base position (build 37); EA = effect allele; NEA = non-effect allele; EAF = effect allele frequency; SE = standard error.

SNV	Chr	BP	EA	NEA	EAF	Type 2 diabetes		Advanced AMD	
						**Beta**	**SE**	**Beta**	**SE**
rs2296173	1	39913351	G	A	0.212	0.065	0.009	-0.007	0.02
rs12088739	1	51506886	G	A	0.09	-0.088	0.013	-0.016	0.029
rs1127655	1	117530507	C	T	0.471	0.044	0.008	-0.016	0.016
rs2493394	1	120471224	G	A	0.107	0.073	0.011	0.001	0.027
rs340874	1	214159256	T	C	0.436	-0.063	0.007	-0.009	0.017
rs2820426	1	219660535	A	G	0.39	-0.052	0.007	0.006	0.017
rs348330	1	229672955	G	A	0.367	0.049	0.008	0.007	0.02
rs2867125	2	622827	T	C	0.172	-0.06	0.01	-0.011	0.021
rs780094	2	27741237	T	C	0.387	-0.069	0.007	0.039	0.017
rs17334919	2	43707385	T	C	0.1	-0.14	0.013	0.066	0.027
rs243019	2	60585806	C	T	0.456	0.057	0.007	0.018	0.016
rs1009358	2	65276452	C	T	0.377	-0.055	0.008	0.008	0.017
rs10169613	2	111934977	T	C	0.472	-0.043	0.008	0.053	0.017
rs12617659	2	121309759	T	C	0.147	-0.069	0.01	0.025	0.024
rs7572970	2	161136656	A	G	0.278	-0.059	0.009	-0.036	0.018
rs13389219	2	165528876	T	C	0.394	-0.072	0.007	0.001	0.017
rs2972144	2	227101411	A	G	0.355	-0.091	0.008	-0.011	0.017
rs7561798	2	228973660	G	A	0.482	0.04	0.007	0.01	0.016
rs1899951	3	12394840	T	C	0.123	-0.112	0.011	0.005	0.025
rs1496653	3	23454790	G	A	0.205	-0.077	0.009	0.022	0.021
rs11926707	3	46925539	T	C	0.374	-0.046	0.008	0.036	0.019
rs2292662	3	63897215	T	C	0.151	-0.063	0.011	0.004	0.022
rs6795735	3	64705365	T	C	0.411	-0.056	0.007	0.125	0.017
rs11708067	3	123065778	G	A	0.239	-0.097	0.009	-0.003	0.02
rs9844972	3	150097635	C	G	0.07	0.096	0.015	0.003	0.034
rs4472028	3	152053250	T	C	0.444	0.045	0.007	-0.02	0.017
rs11925227	3	170766618	A	G	0.183	-0.053	0.01	-0.021	0.025
rs7651090	3	185513392	G	A	0.313	0.12	0.008	0.004	0.018
rs3887925	3	186665645	C	T	0.447	-0.047	0.008	0.009	0.017
rs6808574	3	187740523	T	C	0.39	-0.055	0.008	-0.026	0.017
rs1801214	4	6303022	C	T	0.4	-0.09	0.007	0.002	0.017
rs17086692	4	53134293	T	G	0.313	-0.047	0.008	-0.005	0.018
rs993380	4	83584496	A	G	0.334	0.051	0.008	0.007	0.017
rs7674212	4	103988899	T	G	0.409	-0.047	0.008	-0.025	0.017
rs11098676	4	123833154	T	C	0.212	-0.054	0.01	0.023	0.02
rs7685296	4	153254121	T	C	0.279	-0.051	0.008	0.019	0.021
rs735949	4	185716232	C	T	0.141	-0.071	0.011	-0.033	0.025
rs1061813	5	14847331	G	A	0.463	0.043	0.007	0.000	0.017
rs4865796	5	53272664	G	A	0.307	-0.053	0.008	0.01	0.018
rs459193	5	55806751	A	G	0.255	-0.071	0.008	-0.015	0.019
rs3900856	5	55833892	A	G	0.039	0.114	0.019	-0.006	0.043
rs2307111	5	75003678	C	T	0.397	-0.041	0.007	-0.038	0.017
rs6878122	5	76427311	G	A	0.318	0.056	0.008	0.002	0.018
rs7729395	5	102100576	T	C	0.051	0.137	0.016	-0.05	0.043
rs10077431	5	112927686	A	C	0.215	-0.049	0.009	0.051	0.021
rs1050226	6	7281654	G	A	0.407	-0.049	0.007	-0.002	0.017
rs7756992	6	20679709	G	A	0.267	0.13	0.008	0.004	0.018
rs2857605	6	31524851	C	T	0.211	-0.067	0.009	-0.047	0.021
rs1063355	6	32627714	T	G	0.398	-0.071	0.008	-0.152	0.02
rs2071479	6	32781112	T	C	0.027	0.147	0.023	-0.069	0.048
rs9369425	6	43810974	G	A	0.292	0.055	0.009	0.044	0.018

© 2021 Kuav V et al. *JAMA Opthalmology.*

rs72892910	6	50816887	T	G	0.172	0.065	0.01	-0.083	0.021
rs853974	6	127068983	T	C	0.262	0.06	0.009	-0.029	0.019
rs2246012	6	131898208	C	T	0.165	0.053	0.009	0.053	0.023
rs622217	6	160766770	C	T	0.484	-0.049	0.008	0.009	0.017
rs17168486	7	14898282	T	C	0.174	0.074	0.009	0.006	0.022
rs2191348	7	15064255	G	T	0.453	-0.065	0.007	0.002	0.016
rs849135	7	28196413	G	A	0.501	0.1	0.007	0.014	0.016
rs2908282	7	44248828	A	G	0.177	0.055	0.009	-0.043	0.022
rs2299383	7	103418846	T	C	0.423	0.041	0.007	-0.014	0.016
rs13239186	7	117510621	T	C	0.302	0.054	0.009	-0.041	0.018
rs7786095	7	156983847	G	A	0.104	-0.074	0.013	0.023	0.027
rs10100265	8	10633159	A	C	0.39	0.049	0.008	0.031	0.017
rs17411031	8	19852310	G	C	0.262	-0.045	0.008	0.001	0.019
rs10087241	8	30863722	G	A	0.405	0.048	0.008	0.013	0.017
rs12681990	8	36859186	C	T	0.164	0.063	0.01	-0.02	0.022
rs516946	8	41519248	T	C	0.239	-0.082	0.009	0.007	0.019
rs7845219	8	95937502	C	T	0.493	-0.042	0.007	-0.031	0.016
rs3802177	8	118185025	A	G	0.311	-0.122	0.008	0.034	0.018
rs2294120	8	146003567	G	A	0.456	-0.044	0.008	-0.031	0.019
rs10974438	9	4291928	C	A	0.351	0.059	0.008	0.009	0.018
rs1063192	9	22003367	G	A	0.44	-0.063	0.007	0.07	0.017
rs10811661	9	22134094	C	T	0.174	-0.157	0.01	0.031	0.022
rs1758632	9	34025640	C	G	0.377	-0.049	0.008	0.021	0.017
rs17791483	9	81898980	G	A	0.063	-0.102	0.015	0.026	0.036
rs2796441	9	84308948	A	G	0.416	-0.072	0.007	0.004	0.017
rs10114341	9	96919182	C	T	0.441	-0.041	0.007	-0.016	0.017
rs687621	9	136137065	G	A	0.325	0.043	0.008	0.015	0.017
rs11257655	10	12307894	T	C	0.207	0.074	0.009	-0.006	0.02
rs2616132	10	71469514	A	G	0.474	0.046	0.008	0.002	0.016
rs2633310	10	75594050	T	G	0.435	-0.044	0.008	0.011	0.017
rs753270	10	80964975	T	C	0.416	-0.053	0.008	0.037	0.017
rs7923866	10	94482076	T	C	0.379	-0.097	0.007	-0.008	0.017
rs7903146	10	114758349	T	C	0.292	0.306	0.008	-0.041	0.018
rs4918796	10	114880342	C	T	0.223	0.062	0.009	-0.007	0.024
rs2421016	10	124167512	T	C	0.474	-0.046	0.007	0.51	0.017
rs2237892	11	2839751	T	C	0.062	-0.096	0.016	0.029	0.034
rs5215	11	17408630	C	T	0.36	0.068	0.007	0.028	0.017
rs7929543	11	49351026	C	A	0.083	0.083	0.014	-0.054	0.031
rs1552224	11	72433098	C	A	0.154	-0.103	0.01	-0.004	0.023
rs10830963	11	92708710	G	C	0.276	0.091	0.008	0.018	0.018
rs7931302	11	128236058	C	A	0.277	0.046	0.008	0.008	0.019
rs67232546	11	128398938	T	C	0.209	0.06	0.01	-0.021	0.021
rs11048456	12	26463082	C	T	0.244	0.049	0.008	0.04	0.019
rs10842994	12	27965150	T	C	0.197	-0.076	0.009	-0.028	0.021
rs2261181	12	66212318	T	C	0.096	0.099	0.012	-0.03	0.027
rs1480474	12	66326943	G	A	0.416	0.041	0.007	0.016	0.017
rs7138300	12	71439589	C	T	0.443	0.044	0.007	0.031	0.016
rs11107116	12	93978504	T	G	0.22	0.047	0.009	-0.023	0.02
rs61953351	12	121456616	T	G	0.25	-0.07	0.009	0.017	0.019
rs940904	12	123491572	G	A	0.257	-0.05	0.008	0.028	0.018
rs825476	12	124568456	C	T	0.419	-0.052	0.007	-0.024	0.017
rs576674	13	33554302	G	A	0.167	0.065	0.01	-0.043	0.022
rs963740	13	51096095	T	A	0.294	-0.048	0.009	0.029	0.018
rs1359790	13	80717156	A	G	0.287	-0.08	0.008	0.038	0.018
rs7144011	14	79940383	T	G	0.221	0.048	0.009	-0.025	0.02
rs4502156	15	62383155	C	T	0.436	-0.041	0.007	0.002	0.017
rs982077	15	63823301	A	G	0.434	0.045	0.007	-0.013	0.017
rs7177055	15	77832762	G	A	0.282	-0.065	0.008	0.000	0.018

© 2021 Kuav V et al. *JAMA Opthalmology.*

rs4932143	15	90372067	G	C	0.279	0.057	0.009	0.035	0.018
rs12910825	15	91511260	G	A	0.36	0.052	0.007	-0.046	0.018
rs9940149	16	300641	A	G	0.179	-0.058	0.01	-0.005	0.022
rs7185735	16	53822651	G	A	0.397	0.106	0.007	-0.028	0.017
rs244415	16	69666683	A	G	0.413	-0.047	0.008	0.005	0.017
rs77258096	16	75243772	A	C	0.101	-0.117	0.013	-0.162	0.028
rs2925979	16	81534790	T	C	0.299	0.053	0.008	0.03	0.018
rs8068804	17	3985864	A	G	0.325	0.059	0.008	-0.039	0.018
rs12945601	17	17653411	T	C	0.386	0.048	0.008	-0.019	0.017
rs11651755	17	36099840	C	T	0.485	0.074	0.008	-0.006	0.016
rs17405722	17	40542501	A	G	0.074	0.087	0.015	-0.028	0.033
rs9911983	17	45885756	C	T	0.433	-0.04	0.007	-0.003	0.017
rs9894220	17	46989154	G	A	0.434	-0.059	0.008	-0.027	0.016
rs302864	17	56757584	A	G	0.087	0.071	0.013	0.009	0.031
rs17631783	17	61687600	T	C	0.263	-0.049	0.009	-0.01	0.019
rs7240767	18	7070642	C	T	0.384	0.045	0.008	-0.003	0.017
rs12970134	18	57884750	A	G	0.265	0.056	0.008	-0.024	0.019
rs10401969	19	19407718	C	T	0.077	0.092	0.013	0.012	0.03
rs8108269	19	46158513	G	T	0.281	0.064	0.008	-0.028	0.018
rs6515236	20	22435749	C	A	0.249	-0.05	0.009	0.015	0.019
rs6059662	20	32675727	A	G	0.337	-0.045	0.008	0.014	0.018
rs4810426	20	43001721	T	C	0.097	0.073	0.013	-0.06	0.028
rs6066138	20	45594711	A	G	0.278	-0.049	0.008	0.019	0.018
rs16988333	22	30552813	G	A	0.09	-0.075	0.013	0.021	0.03
rs4823182	22	44377442	G	A	0.336	0.048	0.008	-0.006	0.017

© 2021 Kuav V et al. *JAMA Opthalmology.*

eTable 10. HbA1c exposure SNVs and their association with advanced AMD.[a] Chr = chromosome; BP = base position (build 37); EA = effect allele; NEA = non-effect allele; EAF = effect allele frequency; SE = standard error.

SNV	Chr	BP	EA	NEA	EAF	HbA1c		Advanced AMD	
						**Beta**	**SE**	**Beta**	**SE**
rs267738	1	150940625	T	G	0.77	0.011	0.002	0.002	0.02
rs857691	1	158626378	T	C	0.272	0.019	0.002	-0.032	0.019
rs17509001	2	24021231	C	T	0.158	0.018	0.002	0.014	0.023
rs12621844	2	48414735	T	C	0.6	0.01	0.002	-0.028	0.017
rs560887	2	169763148	C	T	0.684	0.028	0.002	-0.014	0.018
rs7616006	3	12267648	A	G	0.574	0.01	0.002	0.021	0.017
rs9818758	3	49382925	A	G	0.203	0.012	0.002	-0.014	0.021
rs11708067	3	123065778	A	G	0.754	0.013	0.002	0.003	0.02
rs8192675	3	170724883	T	C	0.691	0.011	0.002	0.023	0.018
rs13134327	4	144659795	A	G	0.334	0.013	0.002	0.038	0.018
rs7756992	6	20679709	G	A	0.286	0.012	0.002	0.004	0.018
rs1800562	6	26093141	G	A	0.928	0.04	0.004	0.063	0.036
rs198846	6	26107463	G	A	0.829	0.022	0.002	0.023	0.023
rs11964178	6	109562035	A	G	0.567	0.01	0.002	-0.026	0.017
rs592423	6	139840693	A	C	0.457	0.009	0.002	-0.007	0.016
rs4607517	7	44235668	A	G	0.202	0.031	0.002	-0.044	0.022
rs6474359	8	41549194	T	C	0.953	0.044	0.005	-0.071	0.046
rs4737009	8	41630405	A	G	0.253	0.021	0.002	-0.004	0.019
rs6980507	8	42383084	A	G	0.401	0.01	0.002	0.022	0.017
rs11558471	8	118185733	A	G	0.675	0.015	0.002	-0.033	0.018
rs2383208	9	22132076	A	G	0.799	0.014	0.002	-0.03	0.022
rs7040409	9	91503236	C	G	0.895	0.028	0.004	-0.033	0.034
rs579459	9	136154168	C	T	0.239	0.011	0.002	0.023	0.019
rs4745982	10	71089843	T	G	0.873	0.095	0.006	-0.057	0.037
rs17747324	10	114752503	C	T	0.249	0.015	0.002	-0.063	0.021
rs3782123	11	205198	C	A	0.321	0.013	0.002	0.001	0.018
rs11603334	11	72432985	G	A	0.815	0.012	0.002	0.003	0.023
rs10830963	11	92708710	G	C	0.294	0.02	0.002	0.018	0.018
rs10774625	12	111910219	G	A	0.506	0.009	0.002	-0.062	0.016
rs282587	13	113351662	G	A	0.151	0.019	0.003	0.012	0.025
rs9604573	13	114542858	T	C	0.274	0.01	0.002	-0.036	0.02
rs11248914	16	293562	T	C	0.647	0.014	0.002	-0.017	0.017
rs837763	16	88853729	T	C	0.555	0.017	0.002	0.017	0.016
rs9914988	17	27183104	A	G	0.788	0.013	0.002	-0.008	0.02
rs1046896	17	80685533	T	C	0.316	0.028	0.002	0.003	0.017
rs17533903	19	17256523	A	G	0.243	0.015	0.002	0.033	0.022
rs4820268	22	37469591	G	A	0.461	0.016	0.002	0.011	0.016

[a] The variants together explain 1.6% of the variation in HbA1c

© 2021 Kuav V et al. *JAMA Opthalmology.*

eTable 11. Fasting glucose exposure SNVs and their association with advanced AMD.[a] Chr = chromosome; BP = base position (build 37); EA = effect allele; NEA = non-effect allele; EAF = effect allele frequency; SE = standard error.

SNV	Chr	BP	EA	NEA	EAF	Fasting glucose		Advanced AMD	
						**Beta**	**SE**	**Beta**	**SE**
rs340874	1	214159256	C	T	0.438	0.013	0.002	0.009	0.017
rs780094	2	27741237	C	T	0.394	0.027	0.002	-0.039	0.017
rs560887	2	169763148	C	T	0.326	0.071	0.003	-0.014	0.018
rs11715915	3	49455330	C	T	0.274	0.012	0.002	0.017	0.018
rs11708067	3	123065778	A	G	0.226	0.023	0.003	0.003	0.02
rs1280	3	170713290	T	C	0.137	0.026	0.003	0.012	0.024
rs7651090	3	185513392	A	G	0.296	-0.013	0.002	-0.004	0.018
rs7708285	5	76425867	G	A	0.261	0.011	0.003	0.005	0.018
rs4869272	5	95539448	C	T	0.323	-0.018	0.002	-0.002	0.018
rs17762454	6	7213200	C	T	0.217	-0.012	0.002	0.004	0.019
rs9368222	6	20686996	C	A	0.281	-0.014	0.002	-0.003	0.018
rs2191349	7	15064309	G	T	0.467	-0.029	0.002	0.001	0.016
rs730497	7	44223721	G	A	0.195	-0.057	0.003	0.045	0.022
rs6943153	7	50791579	C	T	0.279	-0.015	0.002	0	0.018
rs983309	8	9177732	G	T	0.097	-0.026	0.003	0.002	0.026
rs11558471	8	118185733	A	G	0.252	0.029	0.002	-0.033	0.018
rs10814916	9	4293150	C	A	0.434	0.016	0.002	0.016	0.017
rs10811661	9	22134094	T	C	0.199	0.024	0.003	-0.031	0.022
rs16913693	9	111680359	T	G	0.017	0.043	0.007	-0.014	0.053
rs3829109	9	139256766	G	A	0.345	0.017	0.003	0.004	0.018
rs11195502	10	113039667	C	T	0.075	0.032	0.004	0.055	0.03
rs7903146	10	114758349	C	T	0.279	-0.022	0.002	0.041	0.018
rs11607883	11	45839709	G	A	0.469	0.021	0.002	0.003	0.017
rs11039182	11	47346723	T	C	0.308	0.023	0.002	-0.019	0.018
rs174576	11	61603510	C	A	0.345	0.02	0.002	0.057	0.017
rs11603334	11	72432985	G	A	0.129	0.019	0.003	0.003	0.023
rs2657879	12	56865338	A	G	0.186	-0.012	0.003	-0.039	0.021
rs10747083	12	133041618	A	G	0.25	0.013	0.002	-0.004	0.017
rs11619319	13	28487599	A	G	0.212	-0.02	0.002	-0.009	0.02
rs576674	13	33554302	G	A	0.129	0.017	0.003	-0.043	0.022
rs3783347	14	100839261	G	T	0.219	0.017	0.003	0.018	0.02
rs4502156	15	62383155	T	C	0.42	0.022	0.002	-0.002	0.017
rs6113722	20	22557099	G	A	0.04	0.035	0.005	-0.06	0.039
rs6072275	20	39743905	G	A	0.142	-0.016	0.003	-0.053	0.023

[a] The variants together explain 7% of the variation in fasting glucose

© 2021 Kuav V et al. *JAMA Opthalmology.*

eTable 12. Fasting insulin exposure SNVs and their association with advanced AMD. [a] Chr = chromosome; BP = base position (build 37); EA = effect allele; NEA = non-effect allele; EAF = effect allele frequency; SE = standard error.

SNV	Chr	BP	EA	NEA	EAF	Fasting insulin		Advanced AMD	
						**Beta**	**SE**	**Beta**	**SE**
rs2820436	1	219640680	C	A	0.322	0.015	0.003	-0.002	0.018
rs1530559	2	135755629	A	G	0.403	0.014	0.003	0.003	0.019
rs10195252	2	165513091	C	T	0.442	-0.016	0.003	-0.002	0.017
rs2972143	2	227116365	G	A	0.371	0.014	0.003	0.013	0.017
rs17036328	3	12390484	T	C	0.097	0.015	0.004	-0.003	0.025
rs3822072	4	89741269	G	A	0.455	-0.009	0.002	0.036	0.016
rs9884482	4	106081636	T	C	0.35	-0.016	0.002	0.02	0.017
rs4865796	5	53272664	A	G	0.292	0.015	0.003	-0.01	0.018
rs459193	5	55806751	G	A	0.217	0.014	0.003	0.015	0.019
rs6912327	6	34764922	C	T	0.243	-0.016	0.003	0.04	0.02
rs2745353	6	127452935	T	C	0.45	0.014	0.003	-0.05	0.016
rs1167800	7	75176196	A	G	0.451	0.016	0.003	0.02	0.017
rs1421085	16	53800954	T	C	0.46	-0.02	0.003	0.03	0.017
rs731839	19	33899065	A	G	0.341	-0.014	0.003	0.028	0.017

[a] The variants together explain 1% of the variation in fasting insulin

© 2021 Kuav V et al. *JAMA Opthalmology.*

eTable 13. Mendelian randomization analysis of modifiable risk factors for advanced AMD, geographic atrophy, and neovascular AMD.

Risk Factor	Method	Advanced AMD		Geographic atrophy		Neovascular AMD	
		**OR (95%)**	**P value**	**OR (95%)**	**P value**	**OR (95%)**	**P value**
**Smoking initiation**	IVW	1.26 (1.13, 1.40)	< 0.001	1.24 (1.03, 1.49)	0.02	1.26 (1.11, 1.43)	< 0.001
	Multivariable MR*	1.19 (1.05, 1.36)	0.007	1.19 (0.96, 1.48)	0.12	1.18 (1.02, 1.37)	0.02
	Weighted Median	1.20 (1.02, 1.40)	0.03	1.31 (1.00, 1.72)	0.05	1.29 (1.08, 1.54)	0.005
	MR Egger	1.40 (0.87, 2.25)	0.16	0.84 (0.38, 1.84)	0.66	1.70 (1.00, 2.88)	0.05
	MR Egger intercept	NA	0.65	NA	0.31	NA	0.26
	MR-PRESSO global test	NA	0.10	NA	0.19	NA	0.16
	MR -PRESSO number of outliers	0	NA	0	NA	0	NA
	MR-PRESSO outlier test	NA	NA	NA	NA	NA	NA
	MR-PRESSO distortion test	NA	NA	NA	NA	NA	NA
**Smoking cessation**	IVW	0.66 (0.50, 0.87)	0.003	0.84 (0.54, 1.30)	0.43	0.67 (0.49, 0.92)	0.01
	Multivariable MR*	0.71 (0.57, 0.88)	0.002	0.83 (0.57, 1.20)	0.32	0.72 (0.57, 0.93)	0.01
	Weighted Median	0.83 (0.56, 1.21)	0.32	0.94 (0.51, 1.73)	0.84	0.87 (0.57, 1.33)	0.51
	MR Egger	0.43 (0.21, 0.87)	0.02	0.45 (0.14, 1.41)	0.17	0.42 (0.18, 0.96)	0.04
	MR Egger intercept	NA	0.20	NA	0.25	NA	0.23
	MR-PRESSO global test	NA	0.34	NA	0.70	NA	0.29
	MR -PRESSO number of outliers	0	NA	0	NA	0	NA
	MR-PRESSO outlier test	NA	NA	NA	NA	NA	NA
	MR-PRESSO distortion test	NA	NA	NA	NA	NA	NA
**Lifetime smoking index**	IVW	1.32 (1.09, 1.59)	0.004	1.25 (0.92, 1.69)	0.15	1.41 (1.14, 1.74)	0.001
	Multivariable MR*	1.48 (1.14, 1.94)	0.004	1.51 (0.96, 2.37)	0.08	1.62 (1.19, 2.19)	0.002
	Weighted Median	1.42 (1.11, 1.81)	0.005	1.21 (0.80, 1.81)	0.36	1.42 (1.08, 1.88)	0.01
	MR Egger	1.87 (0.88, 3.99)	0.11	2.04 (0.60, 6.89)	0.25	1.75 (0.75, 4.07)	0.19
	MR Egger intercept	NA	0.35	NA	0.41	NA	0.60
	MR-PRESSO global test	NA	0.002	NA	0.03	NA	0.006
	MR -PRESSO number of outliers	0	NA	1	NA	0	NA
	MR-PRESSO outlier test	NA (NA, NA)	NA	1.18 (0.89, 1.57)	0.24	NA (NA, NA)	NA
	MR-PRESSO distortion test	NA	NA	NA	0.627	NA	NA
**Age of smoking initiation**	IVW	0.89 (0.39, 2.01)	0.77	1.18 (0.30, 4.68)	0.82	0.88 (0.35, 2.22)	0.78
	Multivariable MR*	0.94 (0.41, 2.11)	0.87	1.23 (0.31, 4.86)	0.77	0.92 (0.36, 2.32)	0.86
	Weighted Median	0.77 (0.28, 2.17)	0.62	0.94 (0.17, 5.20)	0.95	0.72 (0.22, 2.35)	0.59
	MR Egger	0.16 (0.00, 8.14)	0.36	10.74 (0.02, 7479.70)	0.48	0.11 (0.00, 9.66)	0.34
	MR Egger intercept	NA	0.38	NA	0.50	NA	0.35
	MR-PRESSO global test	NA	0.75	NA	0.91	NA	0.71
	MR -PRESSO number of outliers	0	NA	0	NA	0	NA
	MR-PRESSO outlier test	NA	NA	NA	NA	NA	NA
	MR-PRESSO distortion test	NA	NA	NA	NA	NA	NA

© 2021 Kuav V et al. *JAMA Opthalmology.*

**Alcoholic drinks per week**	IVW	1.57 (1.03, 2.40)	0.04	2.70 (1.48, 4.94)	0.001	1.49 (0.95, 2.34)	0.09
	Multivariable MR**	1.70 (1.27, 2.27)	< 0.001	2.61 (1.58, 4.32)	< 0.001	1.60 (1.15, 2.22)	0.005
	Weighted Median	2.04 (1.23, 3.39)	0.006	2.43 (0.99, 5.96)	0.05	2.11 (1.19, 3.74)	0.01
	MR Egger	2.01 (0.98, 4.10)	0.06	2.25 (0.79, 6.36)	0.13	2.06 (0.97, 4.39)	0.06
	MR Egger intercept	NA	0.40	NA	0.67	NA	0.29
	MR-PRESSO global test	NA	<0.001	NA	0.02	NA	<0.001
	MR -PRESSO number of outliers	3	NA	1	NA	2	NA
	MR-PRESSO outlier test	1.47 (1.02, 2.10)	0.04	2.50 (1.42, 4.42)	0.002	1.48 (0.99, 2.22)	0.06
	MR-PRESSO distortion test	NA	0.67	NA	0.76	NA	0.98
**Body mass index**	IVW	1.03 (0.90, 1.18)	0.70	1.03 (0.83, 1.29)	0.77	1.02 (0.87, 1.19)	0.80
	Weighted Median	0.98 (0.80, 1.20)	0.82	1.05 (0.71, 1.54)	0.81	0.92 (0.73, 1.16)	0.51
	MR Egger	0.76 (0.55, 1.06)	0.11	1.03 (0.60, 1.75)	0.92	0.68 (0.47, 0.99)	0.04
	MR Egger intercept	NA	0.05	NA	0.98	NA	0.02
	MR-PRESSO global test	NA	<0.001	NA	0.03	NA	0.001
	MR -PRESSO number of outliers	1	NA	1	NA	1	NA
	MR-PRESSO outlier test	1.06 (0.93, 1.21)	0.41	1.01 (0.82, 1.25)	0.91	1.06 (0.91, 1.23)	0.45
	MR-PRESSO distortion test	NA	0.90	NA	0.05	NA	0.95
**Systolic blood pressure**	IVW	1.01 (0.85, 1.21)	0.91	1.06 (0.82, 1.38)	0.65	0.97 (0.80, 1.18)	0.76
	Weighted Median	1.09 (0.88, 1.35)	0.44	1.26 (0.88, 1.79)	0.21	0.90 (0.70, 1.15)	0.41
	MR Egger	1.66 (0.65, 4.20)	0.29	3.11 (0.79, 12.27)	0.10	1.16 (0.41, 3.30)	0.78
	MR Egger intercept	NA	0.29	NA	0.12	NA	0.73
	MR-PRESSO global test	NA	<0.001	NA	0.10	NA	0.003
	MR -PRESSO number of outliers	0x	NA	0	NA	0	NA
	MR-PRESSO outlier test	NA	NA	NA	NA	NA	NA
	MR-PRESSO distortion test	NA	NA	NA	NA	NA	NA
**Diastolic blood pressure**	IVW	0.82 (0.58, 1.16)	0.27	0.93 (0.59, 1.48)	0.77	0.82 (0.57, 1.19)	0.29
	Weighted Median	1.13 (0.78, 1.63)	0.51	0.89 (0.51, 1.57)	0.69	0.98 (0.66, 1.47)	0.94
	MR Egger	0.70 (0.15, 3.32)	0.66	0.29 (0.04, 2.15)	0.22	0.93 (0.18, 4.79)	0.93
	MR Egger intercept	NA	0.84	NA	0.24	NA	0.88
	MR-PRESSO global test	NA	<0.001	NA	<0.001	NA	<0.001
	MR -PRESSO number of outliers	3	NA	1	NA	2	NA
	MR-PRESSO outlier test	0.90 (0.66, 1.22)	0.51	0.99 (0.63, 1.56)	0.96	0.87 (0.62, 1.21)	0.41
	MR-PRESSO distortion test	NA	0.26	NA	0.04	NA	0.57
**Type 2 diabetes**	IVW	0.89 (0.77, 1.02)	0.10	0.94 (0.82, 1.07)	0.35	0.88 (0.75, 1.02)	0.09
	Weighted Median	0.89 (0.81, 0.97)	0.007	0.86 (0.75, 0.99)	0.03	0.88 (0.80, 0.98)	0.02
	MR Egger	0.98 (0.71, 1.36)	0.92	0.86 (0.64, 1.16)	0.33	1.01 (0.71, 1.43)	0.96
	MR Egger intercept	NA	0.50	NA	0.53	NA	0.38
	MR-PRESSO global test	NA	<0.001	NA	<0.001	NA	<0.001
	MR -PRESSO number of outliers	6	NA	2	NA	6	NA
	MR-PRESSO outlier test	0.93 (0.88, 0.98)	0.01	0.96 (0.88, 1.05)	0.39	0.91 (0.85, 0.96)	0.002

© 2021 Kuav V et al. *JAMA Opthalmology.*

	MR-PRESSO distortion test	NA	0.04	NA	0.39	NA	0.13
**HbA1c**	IVW	0.72 (0.45, 1.16)	0.18	0.74 (0.36, 1.50)	0.40	0.75 (0.48, 1.15)	0.19
	Weighted Median	0.59 (0.34, 1.03)	0.07	0.57 (0.23, 1.42)	0.22	0.68 (0.37, 1.27)	0.23
	MR Egger	0.72 (0.28, 1.84)	0.49	0.48 (0.12, 1.95)	0.31	0.74 (0.31, 1.75)	0.49
	MR Egger intercept	NA	0.99	NA	0.49	NA	0.98
	MR-PRESSO global test	NA	0.001	NA	0.04	NA	0.16
	MR -PRESSO number of outliers	1	NA	0	NA	0	NA
	MR-PRESSO outlier test	0.77 (0.50, 1.18)	0.24	NA	NA	NA	NA
	MR-PRESSO distortion test	NA	0.71	NA	NA	NA	NA
**Fasting glucose**	IVW	0.82 (0.59, 1.14)	0.24	0.83 (0.53, 1.31)	0.43	0.83 (0.57, 1.20)	0.32
	Weighted Median	0.83 (0.55, 1.26)	0.38	1.07 (0.54, 2.10)	0.85	0.73 (0.46, 1.15)	0.17
	MR Egger	0.48 (0.25, 0.91)	0.02	0.54 (0.22, 1.36)	0.19	0.44 (0.21, 0.90)	0.03
	MR Egger intercept	NA	0.06	NA	0.29	NA	0.05
	MR-PRESSO global test	NA	0.03	NA	0.43	NA	0.03
	MR -PRESSO number of outliers	1	NA	0	NA	1	NA
	MR-PRESSO outlier test	0.76 (0.57, 1.03)	0.09	NA	NA	0.75 (0.55, 1.01)	0.07
	MR-PRESSO distortion test	NA	0.70	NA	NA	NA	0.57
**Fasting insulin**	IVW	0.45 (0.20, 1.01)	0.05	0.38 (0.13, 1.11)	0.08	0.48 (0.18, 1.28)	0.14
	Weighted Median	0.52 (0.20, 1.32)	0.17	0.55 (0.13, 2.33)	0.42	0.45 (0.15, 1.38)	0.16
	MR Egger	2.65 (0.01, 498.16)	0.72	1332.90 (1.63, 1092910.98)	0.04	0.36 (0.00, 266.10)	0.76
	MR Egger intercept	NA	0.50	NA	0.02	NA	0.93
	MR-PRESSO global test	NA	0.08	NA	0.02	NA	0.40
	MR -PRESSO number of outliers	0	NA	0	NA	0	NA
	MR-PRESSO outlier test	NA	NA	NA	NA	NA	NA
	MR-PRESSO distortion test	NA	NA	NA	NA	NA	NA

* adjusted for alcohol intake
**adjusted for smoking behavior

© 2021 Kuav V et al. *JAMA Opthalmology.*

eFigure 1. Scatter plot of SNV-smoking initiation and SNV-AMD association estimates for the smoking initiation instrument.

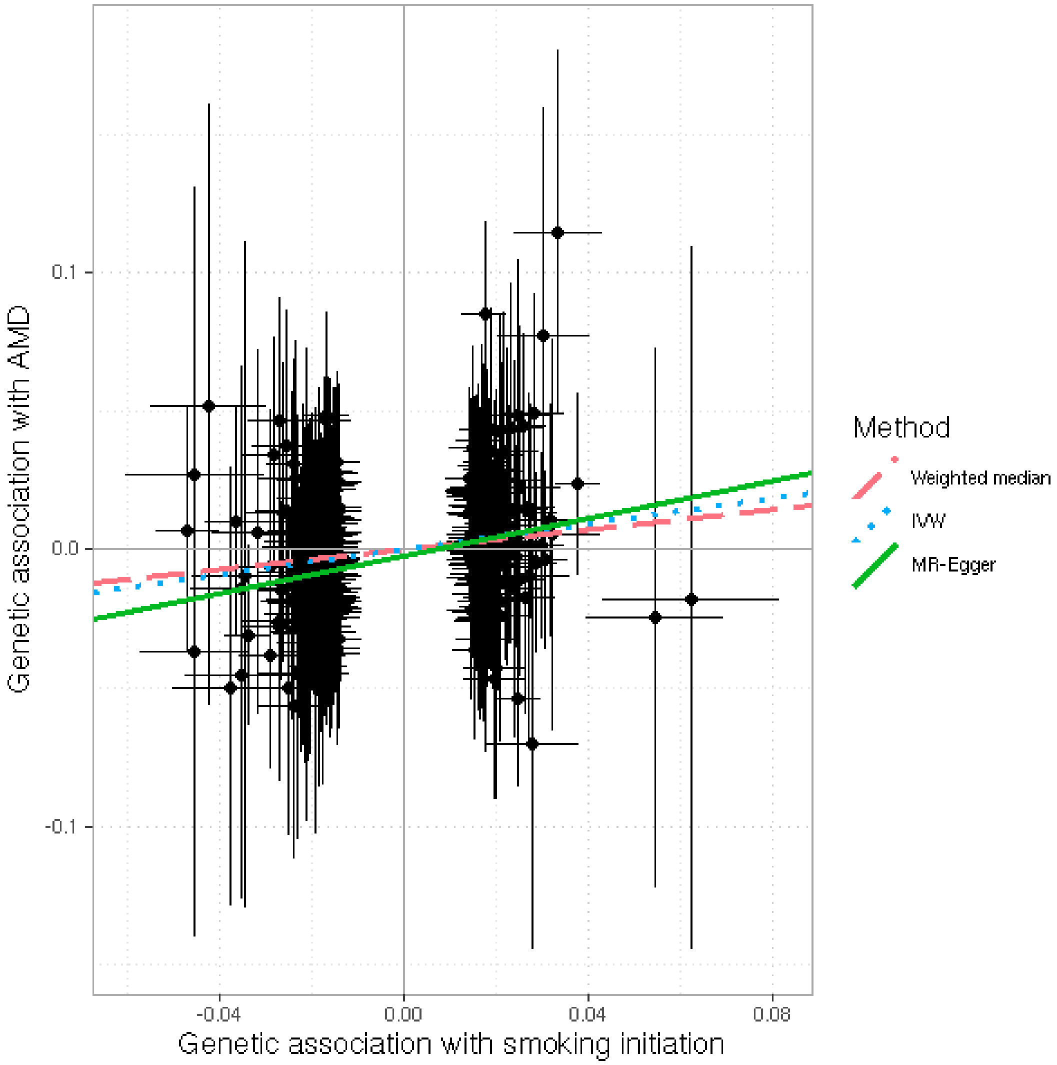

© 2021 Kuav V et al. *JAMA Opthalmology.*

eFigure 2. Scatter plot of SNV-smoking cessation and SNV-AMD association estimates for the smoking cessation instrument.

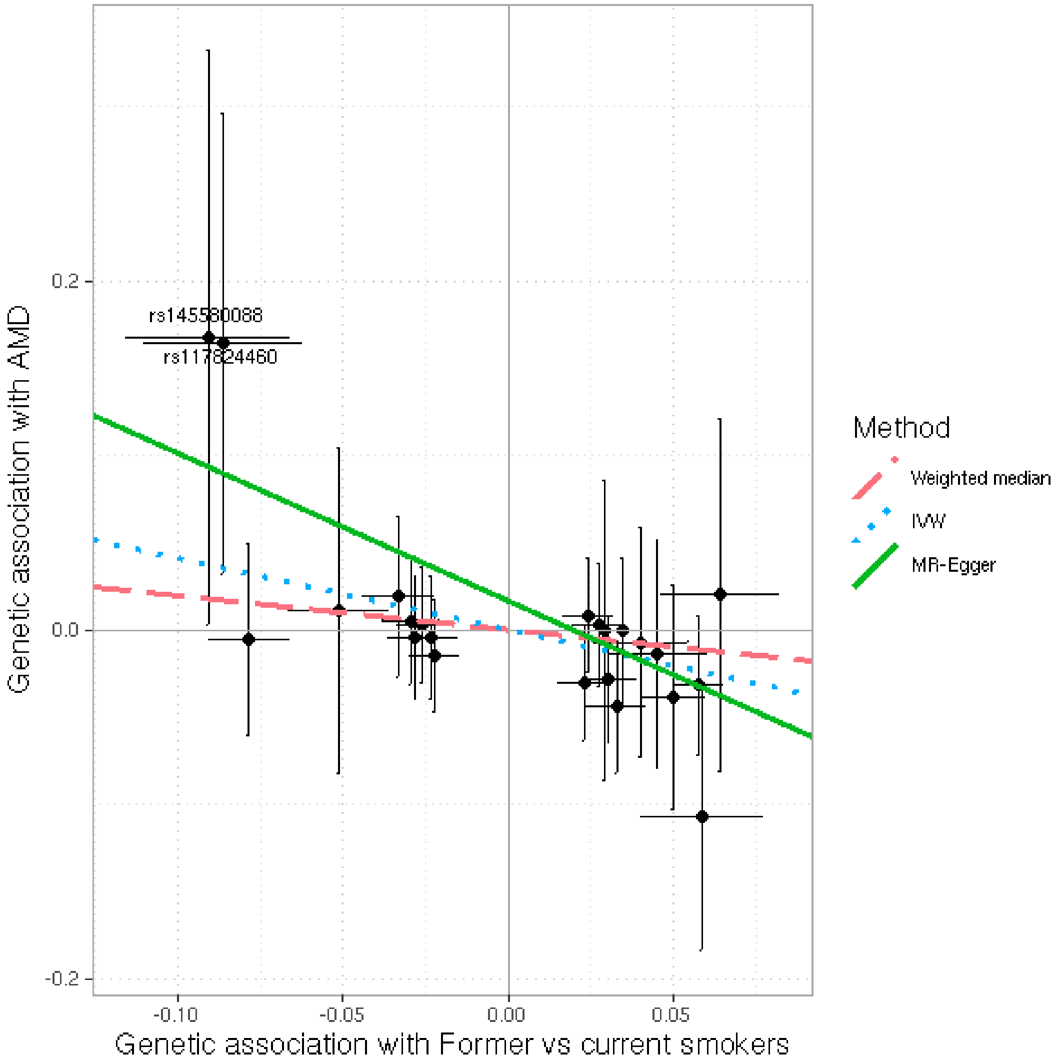

© 2021 Kuav V et al. *JAMA Opthalmology.*

eFigure 3. Scatter plot of SNV-lifetime smoking and SNV-AMD association estimates for the lifetime smoking instrument.

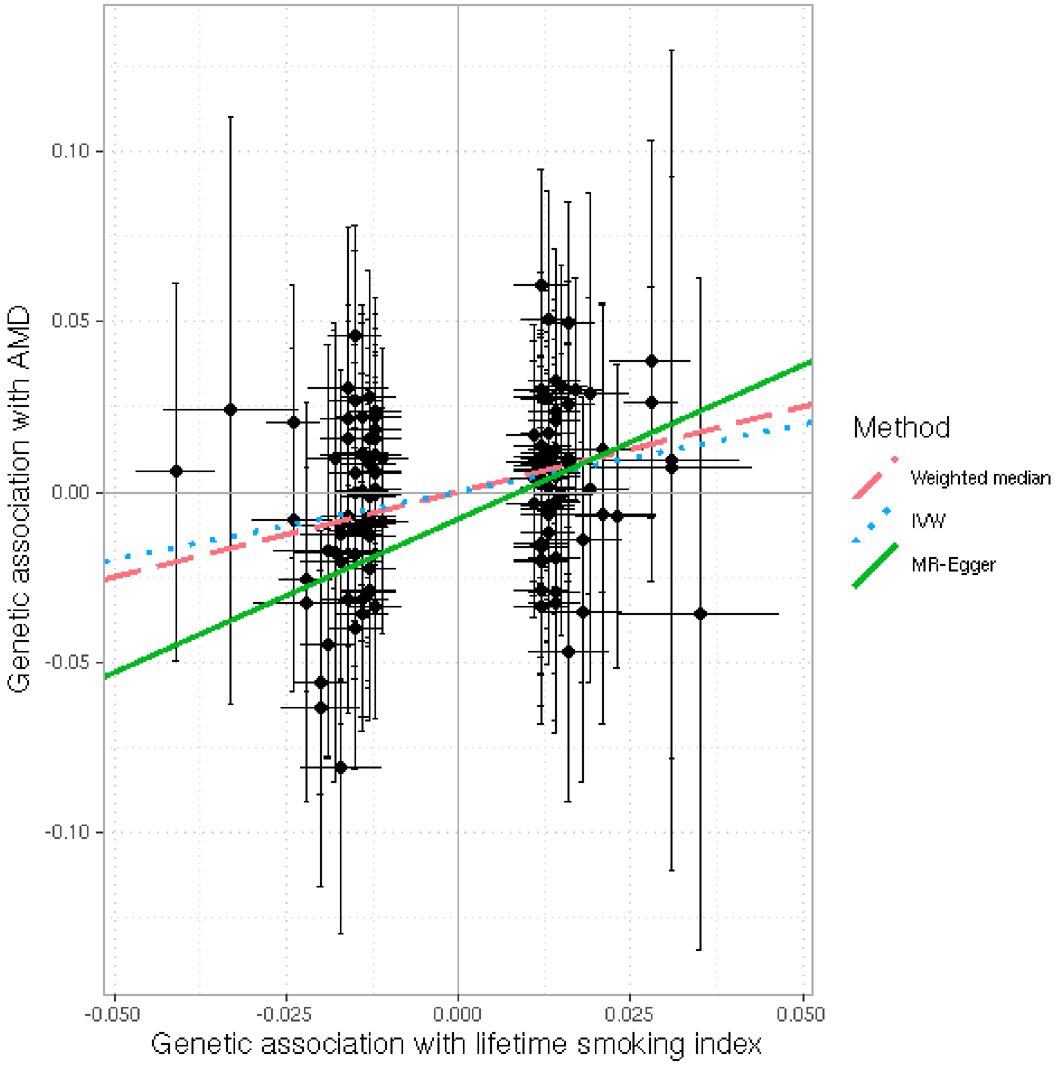

© 2021 Kuav V et al. *JAMA Opthalmology.*

eFigure 4. Scatter plot of SNV-age at smoking initiation and SNV-AMD association estimates for the age at smoking initiation instrument.

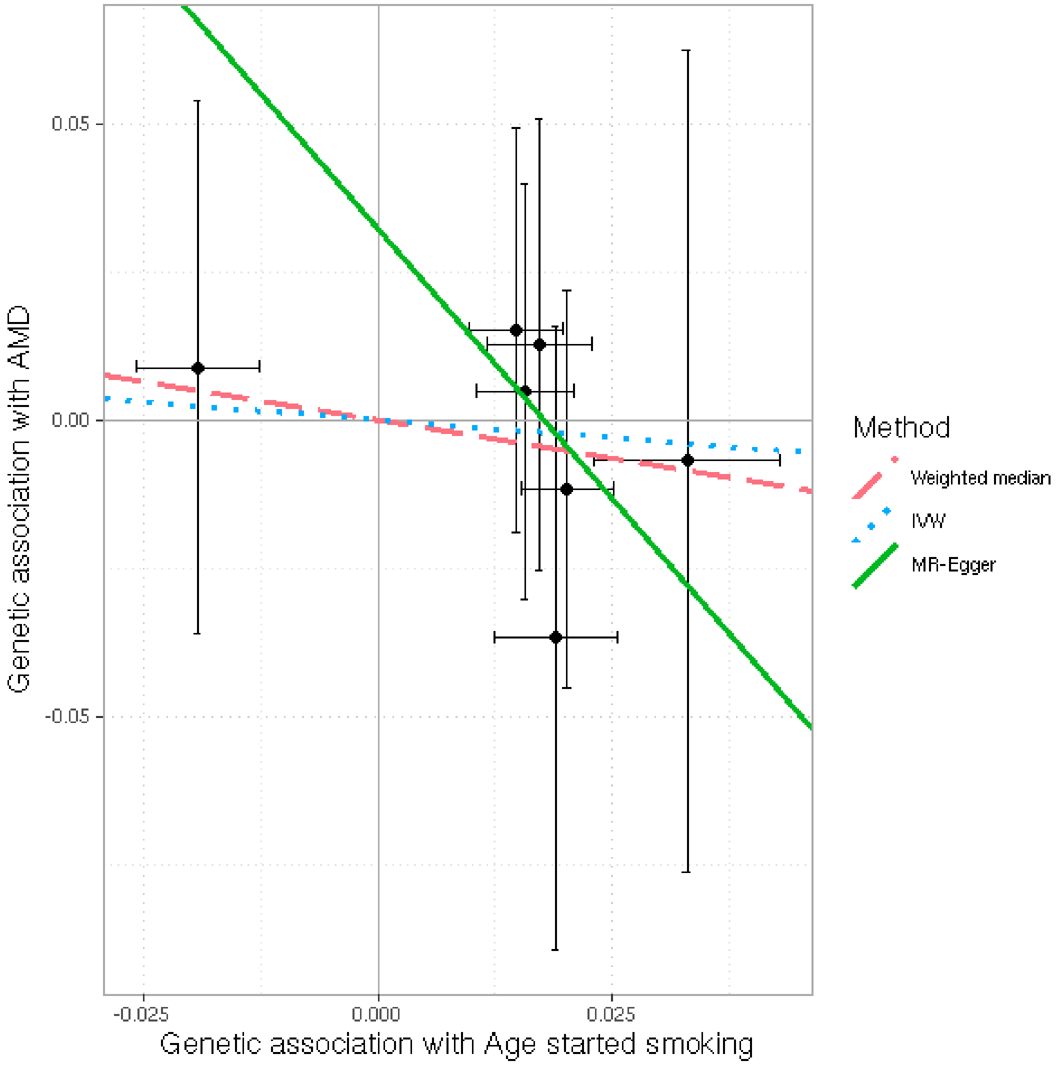

© 2021 Kuav V et al. *JAMA Opthalmology.*

eFigure 5. Scatter plot of SNV-alcoholic drinks per week and SNV-AMD association estimates for the alcohol intake per week instrument.

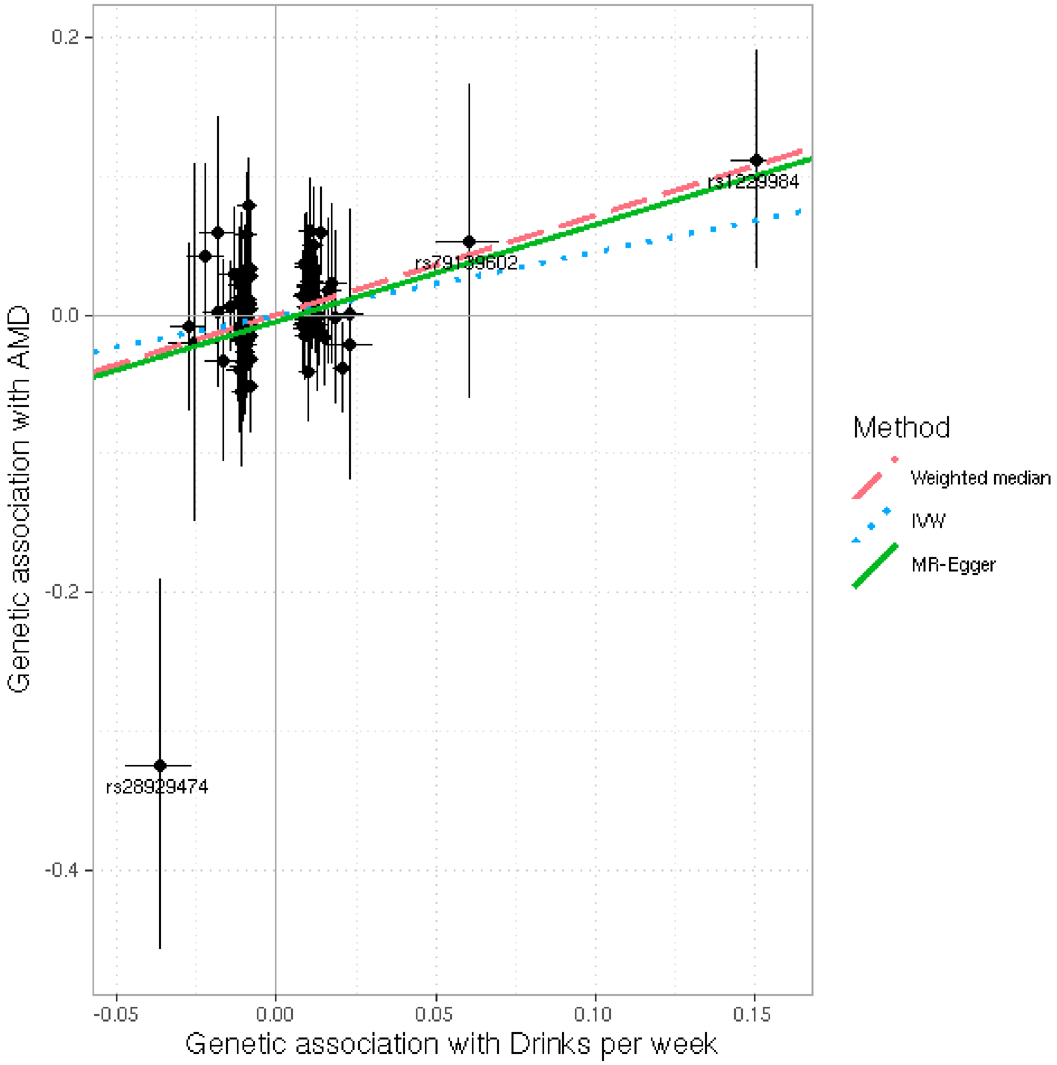

© 2021 Kuav V et al. *JAMA Opthalmology.*

eFigure 6. Scatter plot of SNV-BMI and SNV-AMD association estimates for the body mass index instrument.

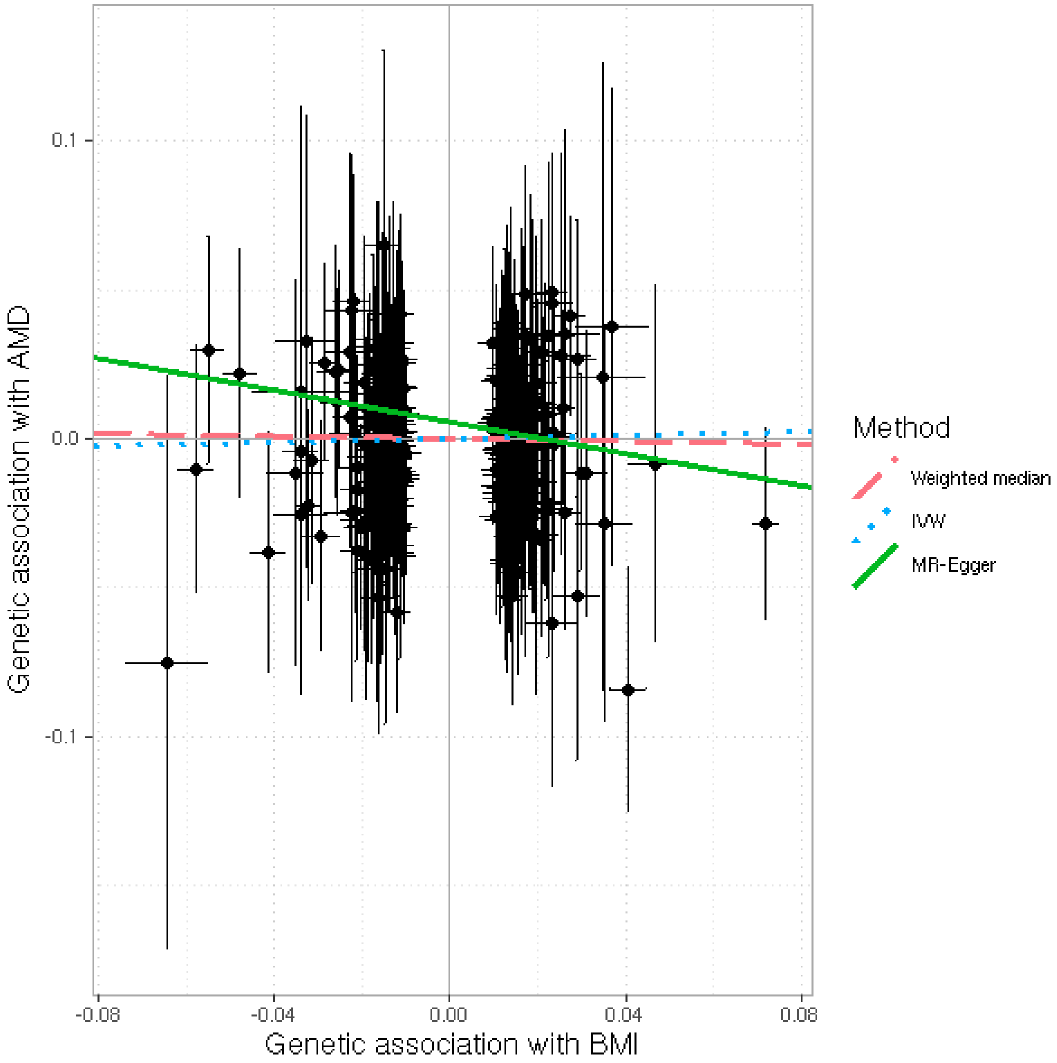

© 2021 Kuav V et al. *JAMA Opthalmology.*

eFigure 7. Scatter plot of SNV-systolic blood pressure and SNV-AMD association estimates for the systolic blood pressure instrument.

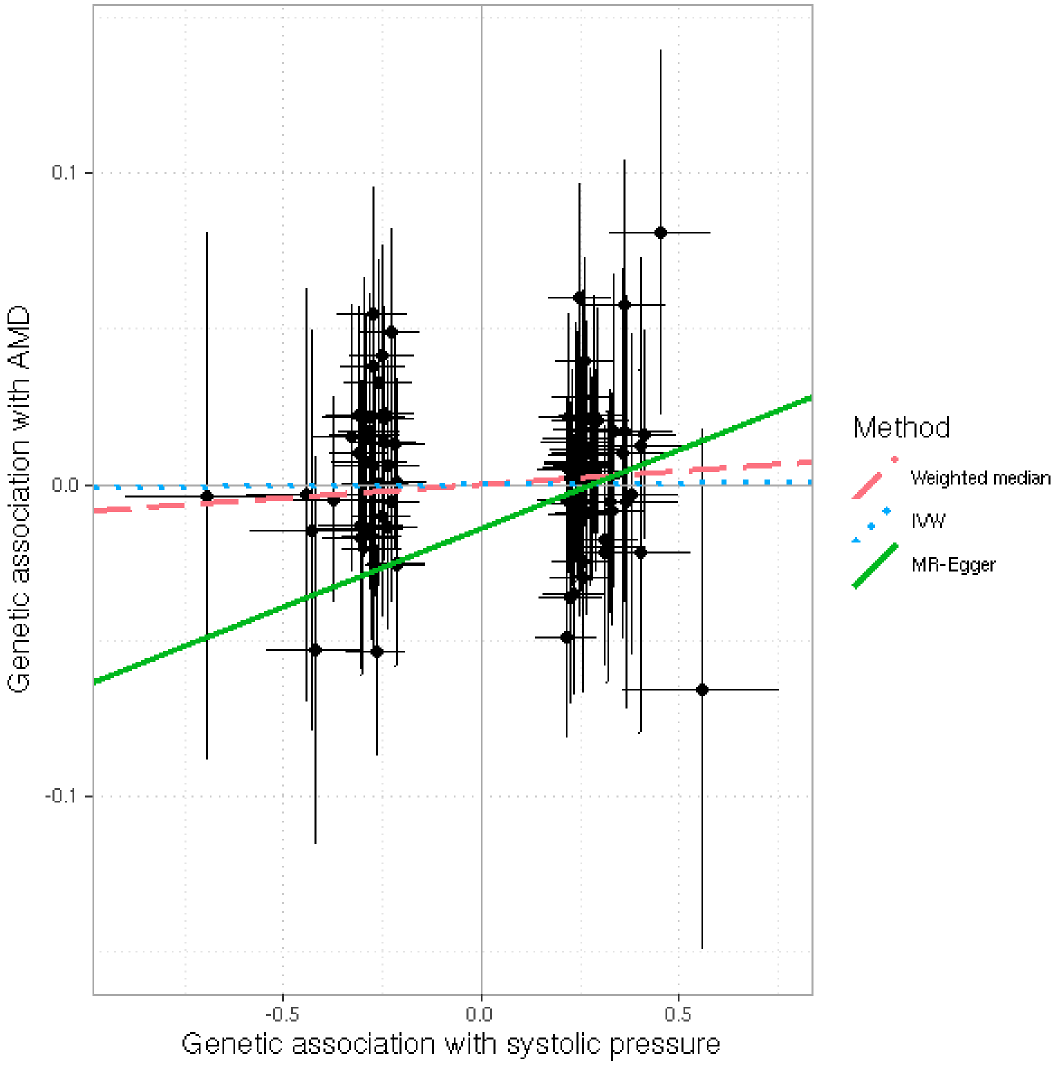

© 2021 Kuav V et al. *JAMA Opthalmology.*

eFigure 8. Scatter plot of SNV-diastolic blood pressure and SNV-AMD association estimates for the diastolic blood pressure instrument.

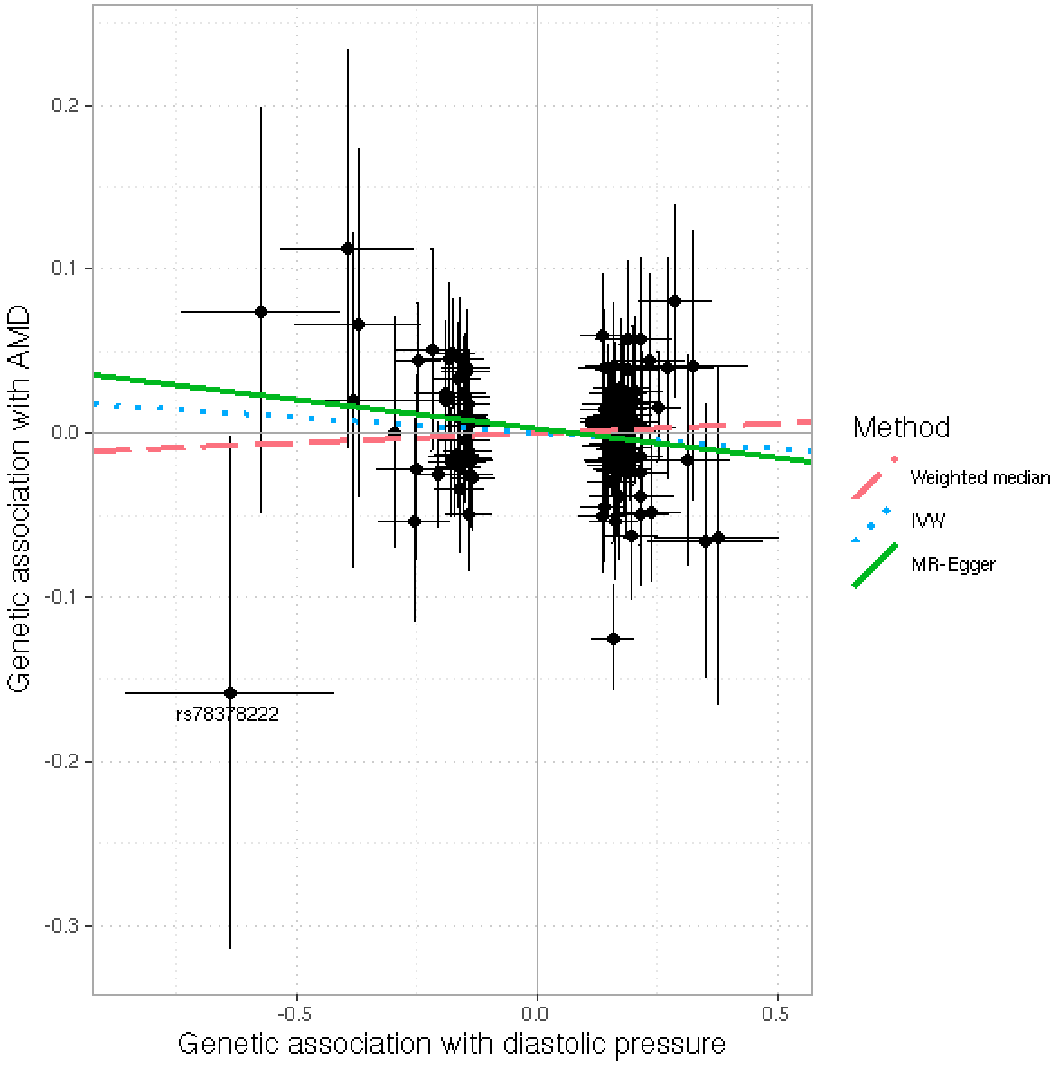

© 2021 Kuav V et al. *JAMA Opthalmology.*

eFigure 9. Scatter plot of SNV–type 2 diabetes and SNV-AMD association estimates for the type 2 diabetes instrument.

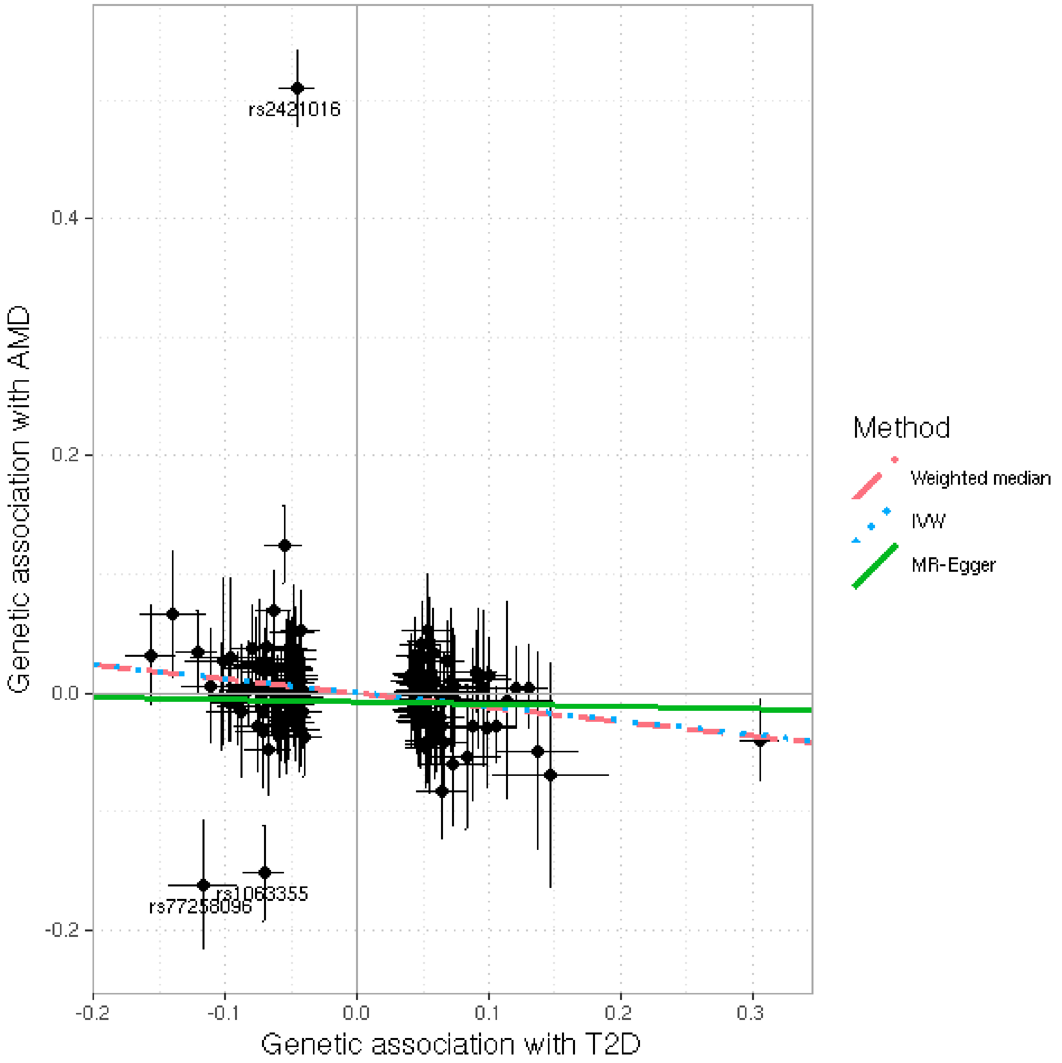

© 2021 Kuav V et al. *JAMA Opthalmology.*

eFigure 10. Scatter plot of SNV-HbA1c and SNV-AMD association estimates for the HbA1c instrument.

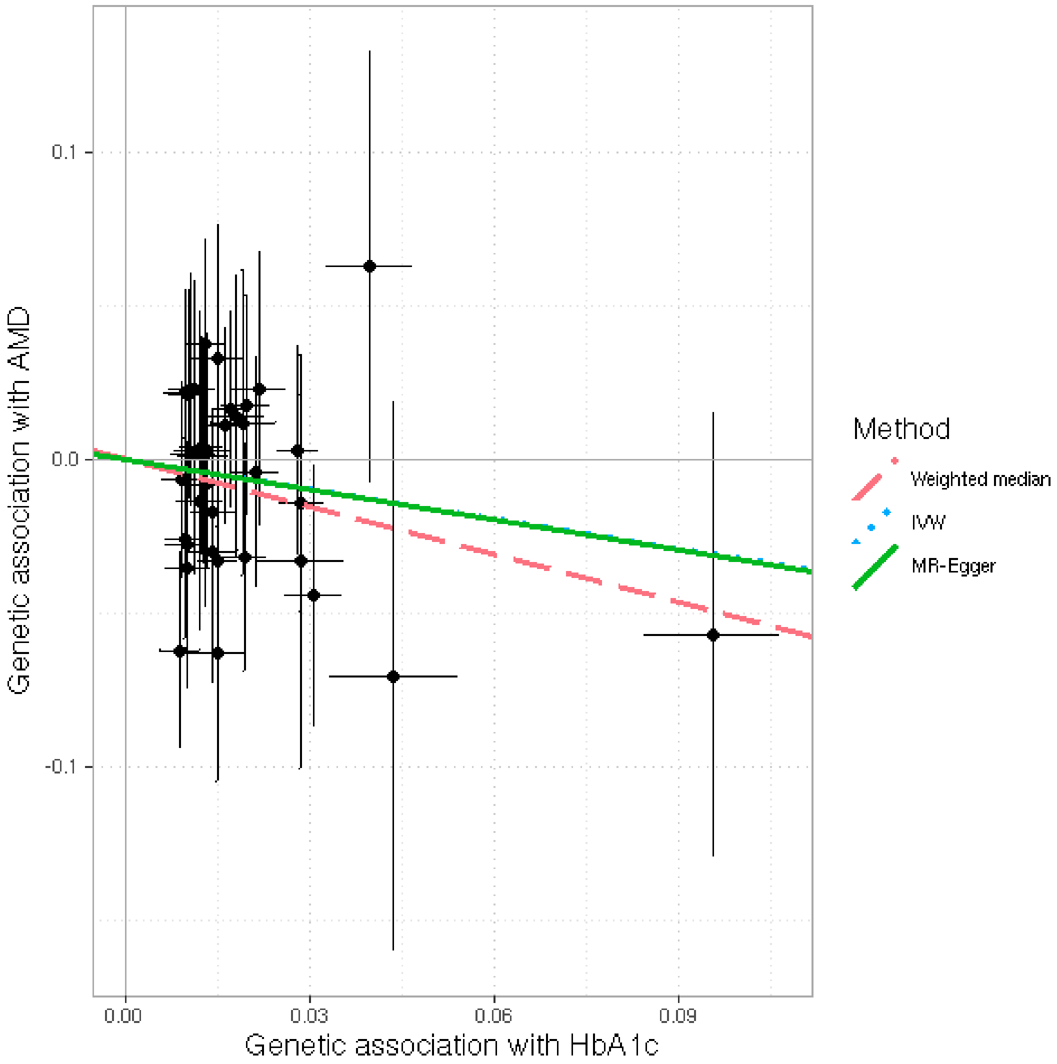

© 2021 Kuav V et al. *JAMA Opthalmology.*

eFigure 11. Scatter plot of SNV-fasting glucose and SNV-AMD association estimates for the fasting glucose instrument.

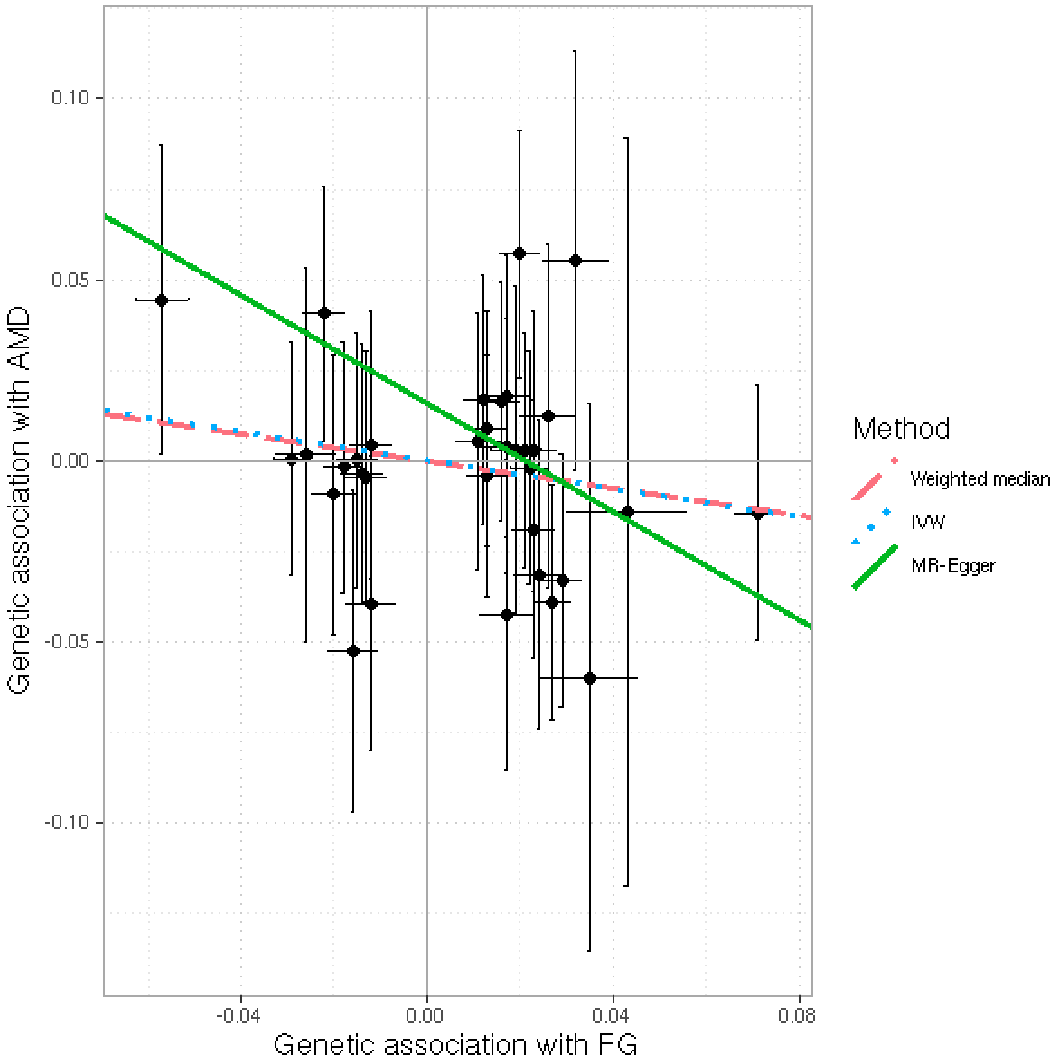

© 2021 Kuav V et al. *JAMA Opthalmology.*

eFigure12. Scatter plot of SNV-fasting insulin and SNV-AMD association estimates for the fasting insulin instrument.

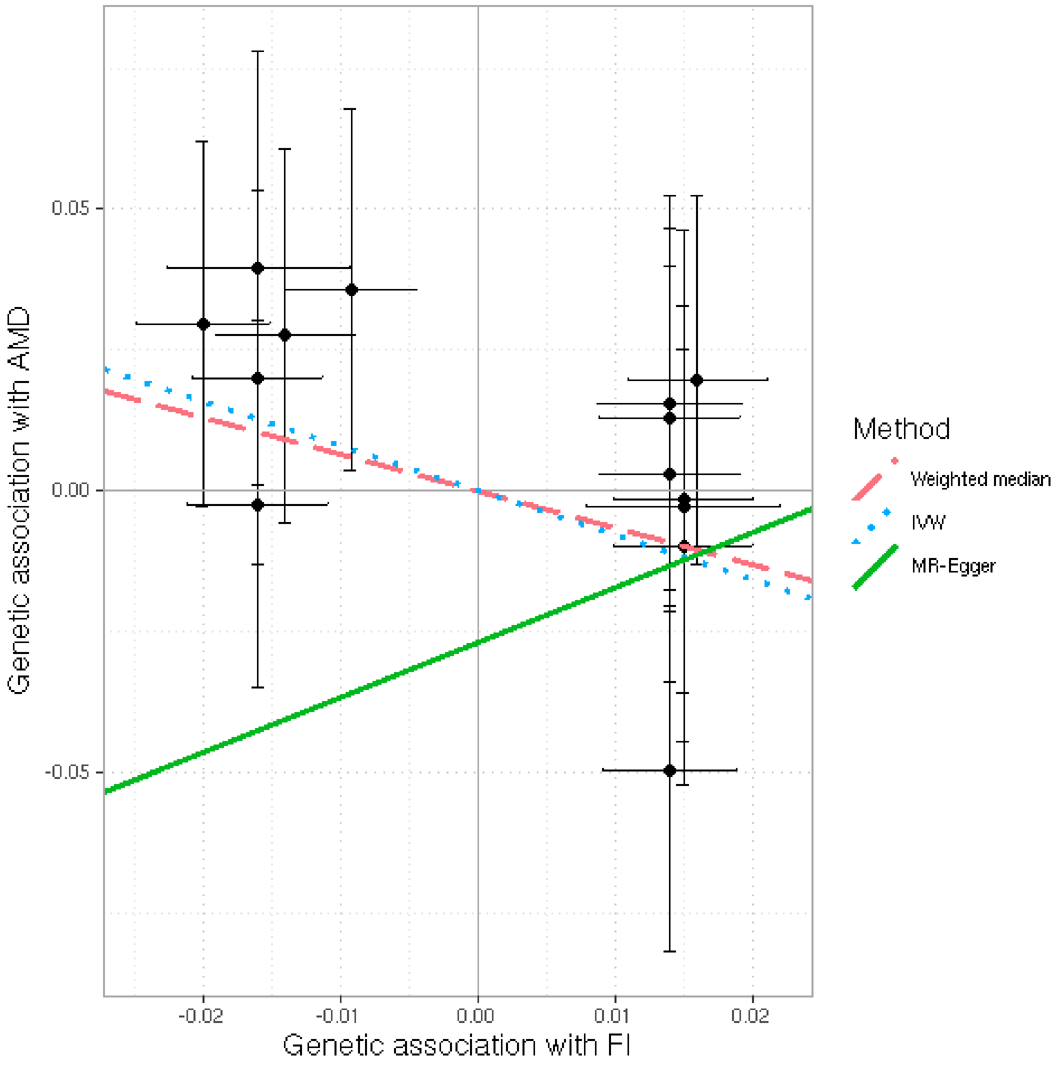

© 2021 Kuav V et al. *JAMA Opthalmology.*

eReferences

[1] Liu M, Jiang Y, Wedow R, et al. Association studies of up to 1.2 million individuals yield new insights into the genetic etiology of tobacco and alcohol use. Nat Genet. 2019;51(2):237-244. doi:10.1038/s41588-018-0307-5

[2] Wootton, R., Richmond, R., Stuijfzand, B., Lawn, R., Sallis, H., Taylor, G., . . . Munafò, M. (2020). Evidence for causal effects of lifetime smoking on risk for depression and schizophrenia: A Mendelian randomisation study. Psychological Medicine, 50(14), 2435-2443. doi:10.1017/S0033291719002678

[3] Sudlow, C. et al. UK biobank: an open access resource for identifying the causes of a wide range of complex diseases of middle and old age. PLoS Med. 12, e1001779 (2015)

[4] Pulit SL, Stoneman C, Morris AP, Wood AR, Glastonbury CA, Tyrrell J, Yengo L, Ferreira T, Marouli E, Ji Y, Yang J, Jones S, Beaumont R, Croteau-Chonka DC, Winkler TW; GIANT Consortium, Hattersley AT, Loos RJF, Hirschhorn JN, Visscher PM, Frayling TM, Yaghootkar H, Lindgren CM. Meta-analysis of genome-wide association studies for body fat distribution in 694 649 individuals of European ancestry. Hum Mol Genet. 2019 Jan 1;28(1):166-174. doi: 10.1093/hmg/ddy327. PMID: 30239722; PMCID: PMC6298238.

[5] Locke AE, Kahali B, Berndt SI, et al. Genetic studies of body mass index yield new insights for obesity biology. Nature. 2015;518:197–206. doi: 10.1038/nature14177.

[6] Hoffmann TJ, Ehret GB, Nandakumar P, et al. Genome-wide association analyses using electronic health records identify new loci influencing blood pressure variation. Nature genetics. 2017;49(1):54-64. doi:10.1038/ng.3715.

[7] Banda Y, Kvale MN, Hoffmann TJ, et al. Characterizing Race/Ethnicity and Genetic Ancestry for 100,000 Subjects in the Genetic Epidemiology Research on Adult Health and Aging (GERA) Cohort. Genetics. 2015;200(4):1285-1295. doi:10.1534/genetics.115.178616

[8] Kvale MN, Hesselson S, Hoffmann TJ, et al. Genotyping Informatics and Quality Control for 100,000 Subjects in the Genetic Epidemiology Research on Adult Health and Aging (GERA) Cohort. Genetics. 2015;200(4):1051-1060. doi:10.1534/genetics.115.178905

[9] International Consortium for Blood Pressure Genome-Wide Association Studies, Ehret GB, Munroe PB, et al. Genetic variants in novel pathways influence blood pressure and cardiovascular disease risk. Nature. 2011;478(7367):103-109. Published 2011 Sep 11. doi:10.1038/nature10405

[10] Xue, A., Wu, Y., Zhu, Z. et al. Genome-wide association analyses identify 143 risk variants and putative regulatory mechanisms for type 2 diabetes. Nat Commun 9, 2941 (2018). https://doi.org/10.1038/s41467-018-04951-w

[11] Morris AP, Voight BF, Teslovich TM, et al. Large-scale association analysis provides insights into the genetic architecture and pathophysiology of type 2 diabetes. Nat Genet. 2012;44:981–990. doi: 10.1038/ng.2383

[12] Wheeler E, Leong A, Liu C-T, Hivert M-F, Strawbridge RJ, Podmore C, et al. (2017) Impact of common genetic determinants of Hemoglobin A1c on type 2 diabetes risk and diagnosis in ancestrally diverse populations: A transethnic genome-wide meta-analysis. PLoS Med 14(9): e1002383. https://doi.org/10.1371/journal.pmed.1002383

[13] Scott RA, Lagou V, Welch RP, et al. Large-scale association analyses identify new loci influencing glycemic traits and provide insight into the underlying biological pathways. Nat Genet. 2012;44(9):991-1005. doi:10.1038/ng.2385

[14] Fritsche, L., Igl, W., Bailey, J. et al. A large genome-wide association study of age-related macular degeneration highlights contributions of rare and common variants. Nat Genet 48, 134–143 (2016).

[15] Bowden J, Holmes MV. Meta-analysis and Mendelian randomization: A review. Res Synth Methods. 2019 Dec;10(4):486-496. doi: 10.1002/jrsm.1346. Epub 2019 Apr 23. PMID: 30861319; PMCID: PMC6973275.

[16] Greco M FD, Minelli C, Sheehan NA, Thompson JR. Detecting pleiotropy in Mendelian randomisation studies with summary data and a continuous outcome. Stat Med. 2015 Sep 20;34(21):2926-40. doi: 10.1002/sim.6522. Epub 2015 May 7. PMID: 25950993.

[17] Hemani G, Bowden J, Davey Smith G. Evaluating the potential role of pleiotropy in Mendelian randomization studies. Hum Mol Genet. 2018;27(R2):R195-R208. doi:10.1093/hmg/ddy163

[18] Burgess S, Davey Smith G, Davies NM et al. Guidelines for performing Mendelian randomization investigations [version 2; peer review: 2 approved]. Wellcome Open Res 2020, 4:186 (https://doi.org/10.12688/wellcomeopenres.15555.2)

[19] Bowden J, Davey Smith G, Haycock PC, Burgess S. Consistent estimation in Mendelian randomization with some invalid instruments using a weighted median estimator. Genet Epidemiol. 2016;40:304–314.

[20] Bowden J, Davey Smith G, Burgess S. Mendelian randomization with invalid instruments: effect estimation and bias detection through Egger regression. Int J Epidemiol. 2015;44:512–525.

© 2021 Kuav V et al. *JAMA Opthalmology.*

[21] Verbanck M, Chen CY, Neale B, Do R. Detection of widespread horizontal pleiotropy in causal relationships inferred from Mendelian randomization between complex traits and diseases. Nat Genet. 2018 May;50(5):693-698. doi: 10.1038/s41588-018-0099-7. Epub 2018 Apr 23.

[22] Burgess S, Thompson SG. Multivariable Mendelian randomization: the use of pleiotropic genetic variants to estimate causal effects. Am J Epidemiol. 2015 Feb 15;181(4):251-60. doi: 10.1093/aje/kwu283. Epub 2015 Jan 27. PMID: 25632051; PMCID: PMC4325677.

© 2021 Kuav V et al. *JAMA Opthalmology.*
