# Supplementary material for: Association of Smoking, Alcohol Consumption, Blood Pressure, Body Mass Index, and Glycemic Risk Factors With Age-Related Macular Degeneration: A Mendelian Randomization Study
Source: JAMA Ophthalmol. 2021 Nov 4;139(12):1299–306. doi: 10.1001/jamaophthalmol.2021.4601 (PMC8569599; doi:10.1001/jamaophthalmol.2021.4601)
Supplement: Supplement 2. — Nonauthor collaborators [file jamaophthalmol-e214601-s002.pdf]

*Indicates required information. Only first name, last name, and suffix will appear in PubMed.

*Group Name(s): International AMD Genomics Consortium (IAMDGC)							
*First Name and Middle Initial(s)	*Last Name	*Suffix (eg, Jr, III)	Academic Degrees	Institution	Location (city, state/province, country)	Role or Contribution, eg, chair, principal investigator	Group (if more than 1 Group listed in the byline) and/or Subgroup (eg, Steering Committee)
Lars G	Fritsche			Center for Statistical Genetics, Department of Biostatistics, University of Michigan	Ann Arbor, Michigan, USA		International AMD Genomics Consortium
Wilmar	Igl			Department of Genetic Epidemiology, University of Regensburg	Regensburg, Germany		International AMD Genomics Consortium
Jessica N	Cooke Bailey			Department of Epidemiology and Biostatistics, Case Western Reserve University School of Medicine	Cleveland, Ohio, USA		International AMD Genomics Consortium
Felix	Grassmann			Institute of Human Genetics, University of Regensburg	Regensburg, Germany		International AMD Genomics Consortium
Sebanti	Sengupta			Center for Statistical Genetics, Department of Biostatistics, University of Michigan	Ann Arbor, Michigan, USA		International AMD Genomics Consortium
Jennifer L	Bragg-Gresham			Center for Statistical Genetics, Department of Biostatistics, University of Michigan	Ann Arbor, Michigan, USA		International AMD Genomics Consortium
				Kidney Epidemiology and Cost Center, Department of Internal Medicine–Nephrology, University of Michigan	Ann Arbor, Michigan, USA		International AMD Genomics Consortium
Kathryn P	Burdon			School of Medicine, Menzies Research Institute Tasmania, University of Tasmania	Hobart, Tasmania, Australia		International AMD Genomics Consortium
Scott J	Hebbring			Center for Human Genetics, Marshfield Clinic Research Foundation	Marshfield, Wisconsin, USA		International AMD Genomics Consortium
Cindy	Wen			Department of Ophthalmology, University of California, San Diego and Veterans Affairs San Diego Health System	La Jolla, California, USA		International AMD Genomics Consortium

*Indicates required information. Only first name, last name, and suffix will appear in PubMed.

*First Name and Middle Initial(s)	*Last Name	*Suffix (eg, Jr, III)	Academic Degrees	Institution	Location (city, state/province, country)	Role or Contribution, eg, chair, principal investigator	Group (if more than 1 Group listed in the byline) and/or Subgroup (eg, Steering Committee)
Mathias	Gorski			Department of Genetic Epidemiology, University of Regensburg	Regensburg, Germany		International AMD Genomics Consortium
Ivana K	Kim			Retina Service, Massachusetts Eye and Ear, Department of Ophthalmology, Harvard Medical School	Boston, Massachusetts, USA		International AMD Genomics Consortium
David	Cho			Department of Ophthalmology, Perelman School of Medicine, University of Pennsylvania	Philadelphia, Pennsylvania, USA		International AMD Genomics Consortium
Donald	Zack			Department of Ophthalmology, Wilmer Eye Institute, Johns Hopkins University School of Medicine	Baltimore, Maryland, USA		International AMD Genomics Consortium
				Department of Molecular Biology and Genetics, Johns Hopkins University School of Medicine	Baltimore, Maryland, USA		International AMD Genomics Consortium
				Department of Neuroscience, Johns Hopkins University School of Medicine	Baltimore, Maryland, USA		International AMD Genomics Consortium
				Institute of Genetic Medicine, Johns Hopkins University School of Medicine	Baltimore, Maryland, USA		International AMD Genomics Consortium
				Institue de la Vision, Université Pierre et Marie Curie	Paris, France		International AMD Genomics Consortium
Eric	Souied			Hôpital Intercommunal de Créteil, Hôpital Henri Mondor, Université Paris Est Créteil	Créteil, France		International AMD Genomics Consortium
Hendrik P N	Scholl			Department of Ophthalmology, Wilmer Eye Institute, Johns Hopkins University School of Medicine	Baltimore, Maryland, USA		International AMD Genomics Consortium
				Department of Ophthalmology, University of Bonn	Bonn, Germany		International AMD Genomics Consortium

*Indicates required information. Only first name, last name, and suffix will appear in PubMed.

*First Name and Middle Initial(s)	*Last Name	*Suffix (eg, Jr, III)	Academic Degrees	Institution	Location (city, state/province, country)	Role or Contribution, eg, chair, principal investigator	Group (if more than 1 Group listed in the byline) and/or Subgroup (eg, Steering Committee)
Elisa	Bala			Louis Stokes Cleveland Veterans Affairs Medical Center	Cleveland, Ohio, USA		International AMD Genomics Consortium
Kristine E	Lee			Department of Ophthalmology and Visual Sciences, University of Wisconsin	Madison, Wisconsin, USA		International AMD Genomics Consortium
David J	Hunter			Department of Epidemiology, Harvard School of Public Health	Boston, Massachusetts, USA		International AMD Genomics Consortium
				Department of Nutrition, Harvard School of Public Health	Boston, Massachusetts, USA		International AMD Genomics Consortium
Rebecca J	Sardell			John P Hussman Institute for Human Genomics, Miller School of Medicine, University of Miami	Miami, Florida, USA		International AMD Genomics Consortium
Paul	Mitchell			Centre for Vision Research, Department of Ophthalmology and Westmead Millennium Institute for Medical Research, University of Sydney	Sydney, New South Wales, Australia		International AMD Genomics Consortium
Joanna E	Merriam			Department of Ophthalmology, Columbia University	New York, New York, USA		International AMD Genomics Consortium
Joshua D	Hoffman			Center for Human Genetics Research, Vanderbilt University Medical Center	Nashville, Tennessee, USA		International AMD Genomics Consortium
Tina	Schick			Department of Ophthalmology, University Hospital of Cologne	Cologne, Germany		International AMD Genomics Consortium
Yara T E	Lechanteur			Department of Ophthalmology, Radboud University Medical Centre	Nijmegen, the Netherlands		International AMD Genomics Consortium
Robyn H	Guymer			Centre for Eye Research Australia, University of Melbourne, Royal Victorian Eye and Ear Hospital	East Melbourne, Victoria, Australia		International AMD Genomics Consortium
Matthew P	Johnson			South Texas Diabetes and Obesity Institute, School of Medicine, University of Texas Rio Grande Valley	Brownsville, Texas, USA		International AMD Genomics Consortium

*Indicates required information. Only first name, last name, and suffix will appear in PubMed.

*First Name and Middle Initial(s)	*Last Name	*Suffix (eg, Jr, III)	Academic Degrees	Institution	Location (city, state/province, country)	Role or Contribution, eg, chair, principal investigator	Group (if more than 1 Group listed in the byline) and/or Subgroup (eg, Steering Committee)
Yingda	Jiang			Department of Biostatistics, Graduate School of Public Health, University of Pittsburgh	Pittsburgh, Pennsylvania, USA		International AMD Genomics Consortium
Chloe M	Stanton			Medical Research Council (MRC) Human Genetics Unit, Institute of Genetics and Molecular Medicine, University of Edinburgh	Edinburgh, UK		International AMD Genomics Consortium
Gabriëlle H S	Buitendijk			Department of Ophthalmology, Erasmus Medical Center	Rotterdam, the Netherlands		International AMD Genomics Consortium
				Department of Epidemiology, Erasmus Medical Center	Rotterdam, the Netherlands		International AMD Genomics Consortium
Xiaowei	Zhan			Center for Statistical Genetics, Department of Biostatistics, University of Michigan	Ann Arbor, Michigan, USA		International AMD Genomics Consortium
				Quantitative Biomedical Research Center, Department of Clinical Science, University of Texas Southwestern Medical Center	Dallas, Texas, USA		International AMD Genomics Consortium
				Center for the Genetics of Host Defense, University of Texas Southwestern Medical Center	Dallas, Texas, USA		International AMD Genomics Consortium
Alan M	Kwong			Center for Statistical Genetics, Department of Biostatistics, University of Michigan	Ann Arbor, Michigan, USA		International AMD Genomics Consortium
Alexis	Boleda			Neurobiology, Neurodegeneration and Repair Laboratory (N-NRL), National Eye Institute, US National Institutes of Health	Bethesda, Maryland, USA		International AMD Genomics Consortium
Matthew	Brooks			Neurobiology, Neurodegeneration and Repair Laboratory (N-NRL), National Eye Institute, US National Institutes of Health	Bethesda, Maryland, USA		International AMD Genomics Consortium

*Indicates required information. Only first name, last name, and suffix will appear in PubMed.

*First Name and Middle Initial(s)	*Last Name	*Suffix (eg, Jr, III)	Academic Degrees	Institution	Location (city, state/province, country)	Role or Contribution, eg, chair, principal investigator	Group (if more than 1 Group listed in the byline) and/or Subgroup (eg, Steering Committee)
Linn	Gieser			Neurobiology, Neurodegeneration and Repair Laboratory (N-NRL), National Eye Institute, US National Institutes of Health	Bethesda, Maryland, USA		International AMD Genomics Consortium
Rinki	Ratnapriya			Neurobiology, Neurodegeneration and Repair Laboratory (N-NRL), National Eye Institute, US National Institutes of Health	Bethesda, Maryland, USA		International AMD Genomics Consortium
Kari E	Branham			Department of Ophthalmology and Visual Sciences, University of Michigan, Kellogg Eye Center	Ann Arbor, Michigan, USA		International AMD Genomics Consortium
Johanna R	Foerster			Center for Statistical Genetics, Department of Biostatistics, University of Michigan	Ann Arbor, Michigan, USA		International AMD Genomics Consortium
John R	Heckenlively			Department of Ophthalmology and Visual Sciences, University of Michigan, Kellogg Eye Center	Ann Arbor, Michigan, USA		International AMD Genomics Consortium
Mohammad I	Othman			Department of Ophthalmology and Visual Sciences, University of Michigan, Kellogg Eye Center	Ann Arbor, Michigan, USA		International AMD Genomics Consortium
Brendan J	Vote			School of Medicine, Menzies Research Institute Tasmania, University of Tasmania	Hobart, Tasmania, Australia		International AMD Genomics Consortium
Helena Hai	Liang			Centre for Eye Research Australia, University of Melbourne, Royal Victorian Eye and Ear Hospital	East Melbourne, Victoria, Australia		International AMD Genomics Consortium
Emmanuelle	Souzeau			Department of Ophthalmology, Flinders Medical Centre, Flinders University	Adelaide, South Australia, Australia		International AMD Genomics Consortium
Ian L	McAllister			Centre for Ophthalmology and Visual Science, Lions Eye Institute, University of Western Australia	Perth, Western Australia, Australia		International AMD Genomics Consortium
Timothy	Isaacs			Centre for Ophthalmology and Visual Science, Lions Eye Institute, University of Western Australia	Perth, Western Australia, Australia		International AMD Genomics Consortium

*Indicates required information. Only first name, last name, and suffix will appear in PubMed.

***First Name and Middle Initial(s)**	***Last Name**	***Suffix (eg, Jr, III)**	Academic Degrees	Institution	Location (city, state/province, country)	Role or Contribution, eg, chair, principal investigator	Group (if more than 1 Group listed in the byline) and/or Subgroup (eg, Steering Committee)
Janette	Hall			Department of Ophthalmology, Flinders Medical Centre, Flinders University	Adelaide, South Australia, Australia		International AMD Genomics Consortium
Stewart	Lake			Department of Ophthalmology, Flinders Medical Centre, Flinders University	Adelaide, South Australia, Australia		International AMD Genomics Consortium
David A	Mackey			School of Medicine, Menzies Research Institute Tasmania, University of Tasmania	Hobart, Tasmania, Australia		International AMD Genomics Consortium
				Centre for Eye Research Australia, University of Melbourne, Royal Victorian Eye and Ear Hospital	East Melbourne, Victoria, Australia		International AMD Genomics Consortium
				Centre for Ophthalmology and Visual Science, Lions Eye Institute, University of Western Australia	Perth, Western Australia, Australia		International AMD Genomics Consortium
Ian J	Constable			Centre for Ophthalmology and Visual Science, Lions Eye Institute, University of Western Australia	Perth, Western Australia, Australia		International AMD Genomics Consortium
Jamie E	Craig			Department of Ophthalmology, Flinders Medical Centre, Flinders University	Adelaide, South Australia, Australia		International AMD Genomics Consortium
Terrie E	Kitchner			Center for Human Genetics, Marshfield Clinic Research Foundation	Marshfield, Wisconsin, USA		International AMD Genomics Consortium
Zhenglin	Yang			Sichuan Provincial Key Laboratory for Human Disease Gene Study, Hospital of the University of Electronic Science and Technology of China and Sichuan Provincial People's Hospital	Chengdu, China		International AMD Genomics Consortium
				Sichuan Translational Medicine Hospital, Chinese Academy of Sciences	Chengdu, China		International AMD Genomics Consortium

*Indicates required information. Only first name, last name, and suffix will appear in PubMed.

*First Name and Middle Initial(s)	*Last Name	*Suffix (eg, Jr, III)	Academic Degrees	Institution	Location (city, state/province, country)	Role or Contribution, eg, chair, principal investigator	Group (if more than 1 Group listed in the byline) and/or Subgroup (eg, Steering Committee)
Zhiguang	Su			Molecular Medicine Research Center, State Key Laboratory of Biotherapy, West China Hospital, Sichuan University	Chengdu, China		International AMD Genomics Consortium
Hongrong	Luo			Department of Ophthalmology, University of California, San Diego and Veterans Affairs San Diego Health System	La Jolla, California, USA		International AMD Genomics Consortium
Daniel	Chen			Department of Ophthalmology, University of California, San Diego and Veterans Affairs San Diego Health System	La Jolla, California, USA		International AMD Genomics Consortium
Hong	Ouyang			Department of Ophthalmology, University of California, San Diego and Veterans Affairs San Diego Health System	La Jolla, California, USA		International AMD Genomics Consortium
Ken	Flagg			Department of Ophthalmology, University of California, San Diego and Veterans Affairs San Diego Health System	La Jolla, California, USA		International AMD Genomics Consortium
Danni	Lin			Department of Ophthalmology, University of California, San Diego and Veterans Affairs San Diego Health System	La Jolla, California, USA		International AMD Genomics Consortium
Guanping	Mao			Department of Ophthalmology, University of California, San Diego and Veterans Affairs San Diego Health System	La Jolla, California, USA		International AMD Genomics Consortium
Henry	Ferreyra			Department of Ophthalmology, University of California, San Diego and Veterans Affairs San Diego Health System	La Jolla, California, USA		International AMD Genomics Consortium
Klaus	Stark			Department of Genetic Epidemiology, University of Regensburg	Regensburg, Germany		International AMD Genomics Consortium

Supplemental Online Content: Nonauthor Collaborators

*Indicates required information. Only first name, last name, and suffix will appear in PubMed.

***First Name and Middle Initial(s)**	***Last Name**	***Suffix (eg, Jr, III)**	Academic Degrees	Institution	Location (city, state/province, country)	Role or Contribution, eg, chair, principal investigator	Group (if more than 1 Group listed in the byline) and/or Subgroup (eg, Steering Committee)
Claudia N von	Strachwitz			EyeCentre Southwest	Stuttgart, Germany		International AMD Genomics Consortium
Armin	Wolf			University Eye Clinic, Ludwig Maximilians University	Munich, Germany		International AMD Genomics Consortium
Caroline	Brandl			Department of Genetic Epidemiology, University of Regensburg	Regensburg, Germany		International AMD Genomics Consortium
				Institute of Human Genetics, University of Regensburg	Regensburg, Germany		International AMD Genomics Consortium
				Department of Ophthalmology, University Hospital Regensburg	Regensburg, Germany		International AMD Genomics Consortium
Guenther	Rudolph			University Eye Clinic, Ludwig Maximilians University	Munich, Germany		International AMD Genomics Consortium
Matthias	Olden			Department of Genetic Epidemiology, University of Regensburg	Regensburg, Germany		International AMD Genomics Consortium
Margaux A	Morrison			Department of Ophthalmology and Visual Sciences, University of Utah	Salt Lake City, Utah, USA		International AMD Genomics Consortium
Denise J	Morgan			Department of Ophthalmology and Visual Sciences, University of Utah	Salt Lake City, Utah, USA		International AMD Genomics Consortium
Matthew	Schu			Department of Medicine (Biomedical Genetics), Boston University Schools of Medicine and Public Health	Boston, Massachusetts, USA		International AMD Genomics Consortium
				Department of Ophthalmology, Boston University Schools of Medicine and Public Health	Boston, Massachusetts, USA		International AMD Genomics Consortium
				Department of Neurology, Boston University Schools of Medicine and Public Health	Boston, Massachusetts, USA		International AMD Genomics Consortium
				Department of Epidemiology, Boston University Schools of Medicine and Public Health	Boston, Massachusetts, USA		International AMD Genomics Consortium

*Indicates required information. Only first name, last name, and suffix will appear in PubMed.

*First Name and Middle Initial(s)	*Last Name	*Suffix (eg, Jr, III)	Academic Degrees	Institution	Location (city, state/province, country)	Role or Contribution, eg, chair, principal investigator	Group (if more than 1 Group listed in the byline) and/or Subgroup (eg, Steering Committee)
				Department of Biostatistics, Boston University Schools of Medicine and Public Health	Boston, Massachusetts, USA		International AMD Genomics Consortium
Jeeyun	Ahn			Department of Ophthalmology, Seoul Metropolitan Government Seoul National University Boramae Medical Center	Seoul, Republic of Korea		International AMD Genomics Consortium
Giuliana	Silvestri			Centre for Experimental Medicine, Queen's University	Belfast, UK		International AMD Genomics Consortium
Evangelia E	Tsironi			Department of Ophthalmology, University of Thessaly, School of Medicine	Larissa, Greece		International AMD Genomics Consortium
Kyu Hyung	Park			Department of Ophthalmology, Seoul National University Bundang Hospital	Seongnam, Republic of Korea		International AMD Genomics Consortium
Lindsay A	Farrer			Department of Medicine (Biomedical Genetics), Boston University Schools of Medicine and Public Health	Boston, Massachusetts, USA		International AMD Genomics Consortium
				Department of Ophthalmology, Boston University Schools of Medicine and Public Health	Boston, Massachusetts, USA		International AMD Genomics Consortium
				Department of Neurology, Boston University Schools of Medicine and Public Health	Boston, Massachusetts, USA		International AMD Genomics Consortium
				Department of Epidemiology, Boston University Schools of Medicine and Public Health	Boston, Massachusetts, USA		International AMD Genomics Consortium
				Department of Biostatistics, Boston University Schools of Medicine and Public Health	Boston, Massachusetts, USA		International AMD Genomics Consortium
Anton	Orlin			Department of Ophthalmology, Weill Cornell Medical College	New York, New York, USA		International AMD Genomics Consortium

*Indicates required information. Only first name, last name, and suffix will appear in PubMed.

*First Name and Middle Initial(s)	*Last Name	*Suffix (eg, Jr, III)	Academic Degrees	Institution	Location (city, state/province, country)	Role or Contribution, eg, chair, principal investigator	Group (if more than 1 Group listed in the byline) and/or Subgroup (eg, Steering Committee)
Alexander	Brucker			Scheie Eye Institute, Department of Ophthalmology, University of Pennsylvania Perelman School of Medicine	Philadelphia, Pennsylvania, USA		International AMD Genomics Consortium
Mingyao	Li			Department of Biostatistics and Epidemiology, University of Pennsylvania Perelman School of Medicine	Philadelphia, Pennsylvania, USA		International AMD Genomics Consortium
Christine A	Curcio			Department of Ophthalmology, University of Alabama at Birmingham	Birmingham, Alabama, USA		International AMD Genomics Consortium
Saddek	Mohand-Saïd			INSERM	Paris, France		International AMD Genomics Consortium
				Institut de la Vision, Department of Genetics	Paris, France		International AMD Genomics Consortium
				Centre National de la Recherche Scientifique (CNRS)	Paris, France		International AMD Genomics Consortium
				Centre Hospitalier National d'Ophtalmologie des Quinze-Vingts	Paris, France		International AMD Genomics Consortium
José-Alain	Sahel			University College London Institute of Ophthalmology, University College London	London, UK		International AMD Genomics Consortium
				INSERM	Paris, France		International AMD Genomics Consortium
				Institut de la Vision, Department of Genetics	Paris, France		International AMD Genomics Consortium
				Centre National de la Recherche Scientifique (CNRS)	Paris, France		International AMD Genomics Consortium
				Centre Hospitalier National d'Ophtalmologie des Quinze-Vingts	Paris, France		International AMD Genomics Consortium
				Fondation Ophtalmologique Adolphe de Rothschild	Paris, France		International AMD Genomics Consortium
				Académie des Sciences–Institut de France	Paris, France		International AMD Genomics Consortium

*Indicates required information. Only first name, last name, and suffix will appear in PubMed.

*First Name and Middle Initial(s)	*Last Name	*Suffix (eg, Jr, III)	Academic Degrees	Institution	Location (city, state/province, country)	Role or Contribution, eg, chair, principal investigator	Group (if more than 1 Group listed in the byline) and/or Subgroup (eg, Steering Committee)
Isabelle	Audo			Department of Molecular Genetics, Institute of Ophthalmology	London, UK		International AMD Genomics Consortium
				INSERM	Paris, France		International AMD Genomics Consortium
				Institut de la Vision, Department of Genetics	Paris, France		International AMD Genomics Consortium
				Centre National de la Recherche Scientifique (CNRS)	Paris, France		International AMD Genomics Consortium
Mustapha	Benchaboune			Centre Hospitalier National d'Ophtalmologie des Quinze-Vingts	Paris, France		International AMD Genomics Consortium
Angela J	Cree			Clinical and Experimental Sciences, Faculty of Medicine, University of Southampton	Southampton, UK		International AMD Genomics Consortium
Christina A	Rennie			University Hospital Southampton	Southampton, UK		International AMD Genomics Consortium
Srinivas V	Goverdhan			Clinical and Experimental Sciences, Faculty of Medicine, University of Southampton	Southampton, UK		International AMD Genomics Consortium
Michelle	Grunin			Department of Ophthalmology, Hadassah Hebrew University Medical Center	Jerusalem, Israel		International AMD Genomics Consortium
Shira	Hagbi-Levi			Department of Ophthalmology, Hadassah Hebrew University Medical Center	Jerusalem, Israel		International AMD Genomics Consortium
Peter	Campochiaro			Department of Ophthalmology, Wilmer Eye Institute, Johns Hopkins University School of Medicine	Baltimore, Maryland, USA		International AMD Genomics Consortium
				Department of Neuroscience, Johns Hopkins University School of Medicine	Baltimore, Maryland, USA		International AMD Genomics Consortium
Nicholas	Katsanis			Center for Human Disease Modeling, Duke University, Durham	North Carolina, USA		International AMD Genomics Consortium

*Indicates required information. Only first name, last name, and suffix will appear in PubMed.

*First Name and Middle Initial(s)	*Last Name	*Suffix (eg, Jr, III)	Academic Degrees	Institution	Location (city, state/province, country)	Role or Contribution, eg, chair, principal investigator	Group (if more than 1 Group listed in the byline) and/or Subgroup (eg, Steering Committee)
				Department of Cell Biology, Duke University, Durham	North Carolina, USA		International AMD Genomics Consortium
				Department of Pediatrics, Duke University, Durham	North Carolina, USA		International AMD Genomics Consortium
Frank G	Holz			Department of Ophthalmology, University of Bonn	Bonn, Germany		International AMD Genomics Consortium
Frédéric	Blond			INSERM	Paris, France		International AMD Genomics Consortium
				Institut de la Vision, Department of Genetics	Paris, France		International AMD Genomics Consortium
				Centre National de la Recherche Scientifique (CNRS)	Paris, France		International AMD Genomics Consortium
Hélène	Blanché			Centre d'Etude du Polymorphisme Humain (CEPH) Fondation Jean Dausset	Paris, France		International AMD Genomics Consortium
Jean-François	Deleuze			Centre d'Etude du Polymorphisme Humain (CEPH) Fondation Jean Dausset	Paris, France		International AMD Genomics Consortium
				Commissariat à l'Energie Atomique et aux Energies Alternatives (CEA), Institut de Génomique, Centre National de Génotypage	Evry, France		International AMD Genomics Consortium
Robert P	Igo	Jr		Department of Epidemiology and Biostatistics, Case Western Reserve University School of Medicine	Cleveland, Ohio, USA		International AMD Genomics Consortium
Barbara	Truitt			Department of Epidemiology and Biostatistics, Case Western Reserve University School of Medicine	Cleveland, Ohio, USA		International AMD Genomics Consortium
Neal S	Peachey			Louis Stokes Cleveland Veterans Affairs Medical Center	Cleveland, Ohio, USA		International AMD Genomics Consortium
				Cole Eye Institute, Cleveland Clinic	Cleveland, Ohio, USA		International AMD Genomics Consortium

Supplemental Online Content: Nonauthor Collaborators

*Indicates required information. Only first name, last name, and suffix will appear in PubMed.

*First Name and Middle Initial(s)	*Last Name	*Suffix (eg, Jr, III)	Academic Degrees	Institution	Location (city, state/province, country)	Role or Contribution, eg, chair, principal investigator	Group (if more than 1 Group listed in the byline) and/or Subgroup (eg, Steering Committee)
Stacy M	Meuer			Department of Ophthalmology and Visual Sciences, University of Wisconsin	Madison, Wisconsin, USA		International AMD Genomics Consortium
Chelsea E	Myers			Department of Ophthalmology and Visual Sciences, University of Wisconsin	Madison, Wisconsin, USA		International AMD Genomics Consortium
Emily L	Moore			Department of Ophthalmology and Visual Sciences, University of Wisconsin	Madison, Wisconsin, USA		International AMD Genomics Consortium
Ronald	Klein			Department of Ophthalmology and Visual Sciences, University of Wisconsin	Madison, Wisconsin, USA		International AMD Genomics Consortium
Michael A	Hauser			Department of Ophthalmology, Duke University Medical Center	Durham, North Carolina, USA		International AMD Genomics Consortium
				Department of Medicine, Duke University Medical Center	Durham, North Carolina, USA		International AMD Genomics Consortium
				Duke Molecular Physiology Institute, Duke University Medical Center	Durham, North Carolina, USA		International AMD Genomics Consortium
Eric A	Postel			Department of Ophthalmology, Duke University Medical Center	Durham, North Carolina, USA		International AMD Genomics Consortium
Monique D	Courtenay			John P Hussman Institute for Human Genomics, Miller School of Medicine, University of Miami	Miami, Florida, USA		International AMD Genomics Consortium
Stephen G	Schwartz			Bascom Palmer Eye Institute, University of Miami Miller School of Medicine	Naples, Florida, USA		International AMD Genomics Consortium
Jaclyn L	Kovach			Bascom Palmer Eye Institute, University of Miami Miller School of Medicine	Naples, Florida, USA		International AMD Genomics Consortium
William K	Scott			John P Hussman Institute for Human Genomics, Miller School of Medicine, University of Miami	Miami, Florida, USA		International AMD Genomics Consortium

*Indicates required information. Only first name, last name, and suffix will appear in PubMed.

*First Name and Middle Initial(s)	*Last Name	*Suffix (eg, Jr, III)	Academic Degrees	Institution	Location (city, state/province, country)	Role or Contribution, eg, chair, principal investigator	Group (if more than 1 Group listed in the byline) and/or Subgroup (eg, Steering Committee)
Gerald	Liew			Centre for Vision Research, Department of Ophthalmology and Westmead Millennium Institute for Medical Research, University of Sydney	Sydney, New South Wales, Australia		International AMD Genomics Consortium
Ava G	Tan			Centre for Vision Research, Department of Ophthalmology and Westmead Millennium Institute for Medical Research, University of Sydney	Sydney, New South Wales, Australia		International AMD Genomics Consortium
Bamini	Gopinath			Centre for Vision Research, Department of Ophthalmology and Westmead Millennium Institute for Medical Research, University of Sydney	Sydney, New South Wales, Australia		International AMD Genomics Consortium
John C	Merriam			Department of Ophthalmology, Columbia University	New York, New York, USA		International AMD Genomics Consortium
R Theodore	Smith			Department of Ophthalmology, Columbia University	New York, New York, USA		International AMD Genomics Consortium
				Department of Ophthalmology, New York University School of Medicine	New York, New York, USA		International AMD Genomics Consortium
Jane C	Khan			Centre for Ophthalmology and Visual Science, Lions Eye Institute, University of Western Australia	Perth, Western Australia, Australia		International AMD Genomics Consortium
				Department of Ophthalmology, Royal Perth Hospital	Perth, Western Australia, Australia		International AMD Genomics Consortium
				Department of Medical Genetics, Cambridge Institute for Medical Research, University of Cambridge	Cambridge, UK		International AMD Genomics Consortium
Humma	Shahid			Department of Medical Genetics, Cambridge Institute for Medical Research, University of Cambridge	Cambridge, UK		International AMD Genomics Consortium

Supplemental Online Content: Nonauthor Collaborators

*Indicates required information. Only first name, last name, and suffix will appear in PubMed.

*First Name and Middle Initial(s)	*Last Name	*Suffix (eg, Jr, III)	Academic Degrees	Institution	Location (city, state/province, country)	Role or Contribution, eg, chair, principal investigator	Group (if more than 1 Group listed in the byline) and/or Subgroup (eg, Steering Committee)
				Department of Ophthalmology, Cambridge University Hospitals National Health Service (NHS) Foundation Trust	Cambridge, UK		International AMD Genomics Consortium
Anthony T	Moore			University College London Institute of Ophthalmology, University College London	London, UK		International AMD Genomics Consortium
				Moorfields Eye Hospital	London, UK		International AMD Genomics Consortium
				Department of Ophthalmology, University of California San Francisco Medical School	San Francisco, California, USA		International AMD Genomics Consortium
J Allie	McGrath			Center for Human Genetics Research, Vanderbilt University Medical Center	Nashville, Tennessee, USA		International AMD Genomics Consortium
Reneé	Laux			Department of Epidemiology and Biostatistics, Case Western Reserve University School of Medicine	Cleveland, Ohio, USA		International AMD Genomics Consortium
Milam A	Brantley	Jr		Department of Ophthalmology and Visual Sciences, Vanderbilt University	Nashville, Tennessee, USA		International AMD Genomics Consortium
Anita	Agarwal			Department of Ophthalmology and Visual Sciences, Vanderbilt University	Nashville, Tennessee, USA		International AMD Genomics Consortium
Lebriz	Ersoy			Department of Ophthalmology, University Hospital of Cologne	Cologne, Germany		International AMD Genomics Consortium
Albert	Caramoy			Department of Ophthalmology, University Hospital of Cologne	Cologne, Germany		International AMD Genomics Consortium
Thomas	Langmann			Department of Ophthalmology, University Hospital of Cologne	Cologne, Germany		International AMD Genomics Consortium
Nicole T M	Saksens			Department of Ophthalmology, Radboud University Medical Centre	Nijmegen, the Netherlands		International AMD Genomics Consortium

*Indicates required information. Only first name, last name, and suffix will appear in PubMed.

*First Name and Middle Initial(s)	*Last Name	*Suffix (eg, Jr, III)	Academic Degrees	Institution	Location (city, state/province, country)	Role or Contribution, eg, chair, principal investigator	Group (if more than 1 Group listed in the byline) and/or Subgroup (eg, Steering Committee)
Eiko K de	Jong			Department of Ophthalmology, Radboud University Medical Centre	Nijmegen, the Netherlands		International AMD Genomics Consortium
Carel B	Hoyng			Department of Ophthalmology, Radboud University Medical Centre	Nijmegen, the Netherlands		International AMD Genomics Consortium
Melinda S	Cain			Centre for Eye Research Australia, University of Melbourne, Royal Victorian Eye and Ear Hospital	East Melbourne, Victoria, Australia		International AMD Genomics Consortium
Andrea J	Richardson			Centre for Eye Research Australia, University of Melbourne, Royal Victorian Eye and Ear Hospital	East Melbourne, Victoria, Australia		International AMD Genomics Consortium
Tammy M	Martin			Casey Eye Institute, Oregon Health and Science University	Portland, Oregon, USA		International AMD Genomics Consortium
John	Blangero			South Texas Diabetes and Obesity Institute, School of Medicine, University of Texas Rio Grande Valley	Brownsville, Texas, USA		International AMD Genomics Consortium
Daniel E	Weeks			Department of Biostatistics, Graduate School of Public Health, University of Pittsburgh	Pittsburgh, Pennsylvania, USA		International AMD Genomics Consortium
				Department of Human Genetics, Graduate School of Public Health, University of Pittsburgh	Pittsburgh, Pennsylvania, USA		International AMD Genomics Consortium
Bal	Dhillon			School of Clinical Sciences, University of Edinburgh	Edinburgh, UK		International AMD Genomics Consortium
Cornelia M	Van Duijn			Department of Epidemiology, Erasmus Medical Center	Rotterdam, the Netherlands		International AMD Genomics Consortium
Kimberly F	Doheny			Center for Inherited Disease Research (CIDR) Institute of Genetic Medicine, Johns Hopkins University School of Medicine	Baltimore, Maryland, USA		International AMD Genomics Consortium

*Indicates required information. Only first name, last name, and suffix will appear in PubMed.

*First Name and Middle Initial(s)	*Last Name	*Suffix (eg, Jr, III)	Academic Degrees	Institution	Location (city, state/province, country)	Role or Contribution, eg, chair, principal investigator	Group (if more than 1 Group listed in the byline) and/or Subgroup (eg, Steering Committee)
Jane	Romm			Center for Inherited Disease Research (CIDR) Institute of Genetic Medicine, Johns Hopkins University School of Medicine	Baltimore, Maryland, USA		International AMD Genomics Consortium
Caroline C W	Klaver			Department of Ophthalmology, Erasmus Medical Center	Rotterdam, the Netherlands		International AMD Genomics Consortium
				Department of Epidemiology, Erasmus Medical Center	Rotterdam, the Netherlands		International AMD Genomics Consortium
Caroline	Hayward			Medical Research Council (MRC) Human Genetics Unit, Institute of Genetics and Molecular Medicine, University of Edinburgh	Edinburgh, UK		International AMD Genomics Consortium
Michael B	Gorin			Department of Ophthalmology, David Geffen School of Medicine, Stein Eye Institute, University of California	Los Angeles, Los Angeles, California, USA		International AMD Genomics Consortium
				Department of Human Genetics, David Geffen School of Medicine, University of California	Los Angeles, Los Angeles, California, USA		International AMD Genomics Consortium
Michael L	Klein			Casey Eye Institute, Oregon Health and Science University	Portland, Oregon, USA		International AMD Genomics Consortium
Paul N	Baird			Centre for Eye Research Australia, University of Melbourne, Royal Victorian Eye and Ear Hospital	East Melbourne, Victoria, Australia		International AMD Genomics Consortium
Anneke I den	Hollander			Department of Ophthalmology, Radboud University Medical Centre	Nijmegen, the Netherlands		International AMD Genomics Consortium
				Department of Human Genetics, Radboud University Medical Centre	Nijmegen, the Netherlands		International AMD Genomics Consortium
Sascha	Fauser			Department of Ophthalmology, University Hospital of Cologne	Cologne, Germany		International AMD Genomics Consortium
John R W	Yates			University College London Institute of Ophthalmology, University College London	London, UK		International AMD Genomics Consortium

Supplemental Online Content: Nonauthor Collaborators

*Indicates required information. Only first name, last name, and suffix will appear in PubMed.

***First Name and Middle Initial(s)**	***Last Name**	***Suffix (eg, Jr, III)**	Academic Degrees	Institution	Location (city, state/province, country)	Role or Contribution, eg, chair, principal investigator	Group (if more than 1 Group listed in the byline) and/or Subgroup (eg, Steering Committee)
				Moorfields Eye Hospital	London, UK		International AMD Genomics Consortium
				Department of Medical Genetics, Cambridge Institute for Medical Research, University of Cambridge	Cambridge, UK		International AMD Genomics Consortium
Rando	Allikmets			Department of Ophthalmology, Columbia University	New York, New York, USA		International AMD Genomics Consortium
				Department of Pathology and Cell Biology, Columbia University	New York, New York, USA		International AMD Genomics Consortium
Jie Jin	Wang			Centre for Vision Research, Department of Ophthalmology and Westmead Millennium Institute for Medical Research, University of Sydney	Sydney, New South Wales, Australia		International AMD Genomics Consortium
Debra A	Schaumberg			Department of Epidemiology, Harvard School of Public Health	Boston, Massachusetts, USA		International AMD Genomics Consortium
				Center for Translational Medicine, Moran Eye Center, University of Utah School of Medicine	Salt Lake City, Utah, USA		International AMD Genomics Consortium
				Division of Preventive Medicine, Brigham and Women’s Hospital, Harvard Medical School	Boston, Massachusetts, USA		International AMD Genomics Consortium
Barbara E K	Klein			Department of Ophthalmology and Visual Sciences, University of Wisconsin	Madison, Wisconsin, USA		International AMD Genomics Consortium
Stephanie A	Hagstrom			Cole Eye Institute, Cleveland Clinic	Cleveland, Ohio, USA		International AMD Genomics Consortium
Itay	Chowers			Department of Ophthalmology, Hadassah Hebrew University Medical Center	Jerusalem, Israel		International AMD Genomics Consortium
Andrew J	Lotery			Clinical and Experimental Sciences, Faculty of Medicine, University of Southampton	Southampton, UK		International AMD Genomics Consortium
Thierry	Léveillard			INSERM	Paris, France		International AMD Genomics Consortium
				Institut de la Vision, Department of Genetics	Paris, France		International AMD Genomics Consortium

*Indicates required information. Only first name, last name, and suffix will appear in PubMed.

*First Name and Middle Initial(s)	*Last Name	*Suffix (eg, Jr, III)	Academic Degrees	Institution	Location (city, state/province, country)	Role or Contribution, eg, chair, principal investigator	Group (if more than 1 Group listed in the byline) and/or Subgroup (eg, Steering Committee)
				Centre National de la Recherche Scientifique (CNRS)	Paris, France		International AMD Genomics Consortium
Kang	Zhang			Department of Ophthalmology, University of California, San Diego and Veterans Affairs San Diego Health System	La Jolla, California, USA		International AMD Genomics Consortium
				Molecular Medicine Research Center, State Key Laboratory of Biotherapy, West China Hospital, Sichuan University	Chengdu, China		International AMD Genomics Consortium
Murray H	Brilliant			Center for Human Genetics, Marshfield Clinic Research Foundation	Marshfield, Wisconsin, USA		International AMD Genomics Consortium
Alex W	Hewitt			School of Medicine, Menzies Research Institute Tasmania, University of Tasmania	Hobart, Tasmania, Australia		International AMD Genomics Consortium
				Centre for Eye Research Australia, University of Melbourne, Royal Victorian Eye and Ear Hospital	East Melbourne, Victoria, Australia		International AMD Genomics Consortium
				Centre for Ophthalmology and Visual Science, Lions Eye Institute, University of Western Australia	Perth, Western Australia, Australia		International AMD Genomics Consortium
Anand	Swaroop			Neurobiology, Neurodegeneration and Repair Laboratory (N-NRL), National Eye Institute, US National Institutes of Health	Bethesda, Maryland, USA		International AMD Genomics Consortium
Emily Y	Chew			Division of Epidemiology and Clinical Applications, Clinical Trials Branch, National Eye Institute, US National Institutes of Health	Bethesda, Maryland, USA		International AMD Genomics Consortium
Margaret A	Pericak-Vance			John P Hussman Institute for Human Genomics, Miller School of Medicine, University of Miami	Miami, Florida, USA		International AMD Genomics Consortium

Supplemental Online Content: Nonauthor Collaborators

*Indicates required information. Only first name, last name, and suffix will appear in PubMed.

*First Name and Middle Initial(s)	*Last Name	*Suffix (eg, Jr, III)	Academic Degrees	Institution	Location (city, state/province, country)	Role or Contribution, eg, chair, principal investigator	Group (if more than 1 Group listed in the byline) and/or Subgroup (eg, Steering Committee)
Margaret	DeAngelis			Department of Ophthalmology and Visual Sciences, University of Utah	Salt Lake City, Utah, USA		International AMD Genomics Consortium
Dwight	Stambolian			Department of Ophthalmology, Perelman School of Medicine, University of Pennsylvania	Philadelphia, Pennsylvania, USA		International AMD Genomics Consortium
Jonathan L	Haines			Department of Epidemiology and Biostatistics, Case Western Reserve University School of Medicine	Cleveland, Ohio, USA		International AMD Genomics Consortium
				Institute for Computational Biology, Case Western Reserve University School of Medicine	Cleveland, Ohio, USA.		International AMD Genomics Consortium
Sudha K	Iyengar			Department of Epidemiology and Biostatistics, Case Western Reserve University School of Medicine	Cleveland, Ohio, USA.		International AMD Genomics Consortium
Bernhard H F	Weber			Institute of Human Genetics, University of Regensburg	Regensburg, Germany		International AMD Genomics Consortium
Gonçalo R	Abecasis			Center for Statistical Genetics, Department of Biostatistics, University of Michigan	Ann Arbor, Michigan, USA		International AMD Genomics Consortium
Iris M	Heid			Department of Genetic Epidemiology, University of Regensburg	Regensburg, Germany		International AMD Genomics Consortium
